# Urea–hydrogen peroxide prompted the selective and controlled oxidation of thioglycosides into sulfoxides and sulfones

**DOI:** 10.3762/bjoc.13.113

**Published:** 2017-06-13

**Authors:** Adesh Kumar Singh, Varsha Tiwari, Kunj Bihari Mishra, Surabhi Gupta, Jeyakumar Kandasamy

**Affiliations:** 1 Department of Chemistry, Indian Institute of Technology, Banaras Hindu University, Varanasi-221005, India

**Keywords:** monosaccharides, oxidation, sulfones, sulfoxides, thioglycosides, urea–hydrogen peroxide

## Abstract

A practical method for the selective and controlled oxidation of thioglycosides to corresponding glycosyl sulfoxides and sulfones is reported using urea–hydrogen peroxide (UHP). A wide range of glycosyl sulfoxides are selectively achieved using 1.5 equiv of UHP at 60 °C while corresponding sulfones are achieved using 2.5 equiv of UHP at 80 °C in acetic acid. Remarkably, oxidation susceptible olefin functional groups were found to be stable during the oxidation of sulfide.

## Introduction

Organosulfur compounds such as sulfides, sulfoxides and sulfones are useful intermediates for the construction of highly functionalized natural products [1–2]. Sulfur moieties are found in several therapeutically important molecules that possess antibacterial, antifungal, anti-ulcer, anti-atherosclerotic, antihypertensive activities, etc. [3–4]. Sulfur compounds also play an important role in carbohydrate synthesis. Thioglycosides, glycosyl sulfoxides and sulfones have been widely used as glycosyl donors in oligosaccharide synthesis which can be activated under mild reaction conditions [5–10]. Glycosyl sulfoxide donors usually provide excellent anomeric selectivity during the synthesis of various glycosyl linkages not only in solution phase but also in solid-phase oligosaccharide synthesis [6–9,11]. Glycosyl sulfones were also used as donors in the preparation of various *C*- and *O*- linked oligosaccharides and functionalized glycols [8–9,12]. In addition, glycosyl sulfones are known to be potential glycosyltransferase inhibitors [13].

Glycosyl sulfoxides and sulfones are prepared from the corresponding sulfides using various oxidizing reagents [5–7,10]. Although a number of oxidation methods were developed for the oxidation of simple organic sulfides to corresponding sulfoxides and sulfones [14–16], there are only limited reports available for the preparation of glycosyl sulfoxides and sulfones from corresponding thioglycosides [5–7,17–23]. Moreover, there is no report available where a given oxidant is suitable for controlled oxidation of thioglycosides to glycosyl sulfoxides and sulfones selectively by altering the reaction conditions. It is also observed that thioglycoside oxidation suffers from low yields, poor selectivity (i.e., sulfoxide vs sulfone), use of inconvenient reaction conditions and expensive oxidants, intolerance of other oxidation susceptible functional groups, etc. Thus, developing a mild and efficient method for the controlled oxidation of sulfides to corresponding glycosyl sulfoxides and sulfones, is of great interest.

The utility of hydrogen peroxide–solid adducts in organic synthesis is well explored [24]. Most of them are found to be stable which can be easily handled and stored. One such solid adduct is urea–hydrogen peroxide (UHP) which is considered to be a safer and efficient alternative to high concentrated aqueous hydrogen peroxide solution [25]. In addition, UHP is also commercially available, inexpensive and nontoxic. The application of UHP as oxidant is well explored in various solution- as well as solid-phase organic syntheses [25–28]. In fact, we have recently reported the oxidation of arylboronic acids into corresponding phenols by using UHP as a selective oxidizing agent [29]. In continuation to our effort in developing green methodologies [29–33], here we disclose an efficient and practical method for the conversion of glycosyl sulfides into sulfoxides and sulfones in a selective and controlled manner using urea–hydrogen peroxide in acetic acid.

## Results and Discussion

Initially, phenyl-2,3,4,6-tetra-*O*-acetyl-1-thio-β-D-glucopyranoside (**1**) was chosen as a substrate for the optimization study and oxidation was performed in various solvents at different temperatures in the presence of urea–hydrogen peroxide (UHP) (Table 1). Polar aprotic solvents such as dichloromethane and acetonitrile gave a negligible amount of corresponding sulfoxide (**1a**) while no sulfone (**1b**) was detected at room temperature even after 6 hours (Table 1, entries 1 and 2). However, protic solvents such as methanol, ethanol, *tert*-butanol and acetic acid were found to be relatively efficient media for the oxidation when compared with dichloromethane and acetonitrile (Table 1, entries 3–6). Among them, acetic acid gave 37% of the glycosyl sulfoxide (**1a**) after 6 h at room temperature with one equiv of UHP (Table 1, entry 6) while alcoholic solvents gave a low yield. When we increased the amount of UHP to 1.5 equiv, the reaction provides only 64% of the desired sulfoxide at room temperature (Table 1, entry 7). Therefore, the reaction was further investigated at elevated temperatures using 1.5 equiv of UHP (Table 1, entries 8 and 9) in acetic acid. Interestingly, the reaction was driven to completion with the desired sulfoxide (**1b**) in 92% yield within 2 h at 60 °C (Table 1, entry 9). It is also worth noting that less than 5% of the corresponding sulfone was detected in the crude product by ^1^H NMR under these conditions.

**Table 1:** Optimization of reaction conditions.^a^

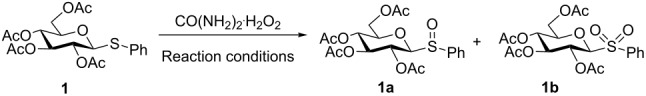						

Entry	UHP (equiv)	Solvent	Temperature	Time	Yield (%)^b^	
					**1a**^c^	**1b**
						
1	1.0	DCM	rt	6 h	<5	n.d.
2	1.0	CH_3_CN	rt	6 h	<10	n.d.
3	1.0	MeOH	rt	6 h	12	n.d.
4	1.0	EtOH	rt	6 h	15	n.d.
5	1.0	*t*-BuOH	rt	6 h	13	n.d.
6	1.0	AcOH	rt	6 h	37	n.d.
7	1.5	AcOH	rt	6 h	64	n.d.
8	1.5	AcOH	40 °C	6 h	90	n.d.
9	1.5	AcOH	60 °C	2 h	92	<5
10	1.5	AcOH	80 °C	2 h	87	7
11	2.0	AcOH	80 °C	3 h	65	31
12	2.5	AcOH	80 °C	10 h	<5	93

^a^Reaction conditions: Thioglycoside (0.25 mmol), solvent (2.5 mL) and urea–hydrogen peroxide (UHP) together stirred for appropriate time at different temperature. ^b^Isolated Yield. ^c^Obtained as *R* and *S* mixture.

Considering the importance of glycosyl sulfones, we further investigated the suitable conditions for the direct oxidation of sulfide to sulfone using UHP in acetic acid. For this, we have tried the reactions with an increased amount of UHP and elevated temperature (Table 1, entries 10–12). It was observed that with 1.5 to 2.0 equiv of UHP at 80 °C, the reaction yields a mixture of sulfoxide **1a** and sulfone **1b** (Table 1, entries 10–11) in different ratio. However, by increasing the amount of UHP to 2.5 equiv, sulfide **1** is fully converted to the corresponding sulfone **1b** in an excellent yield, i.e., 93% in 10 h at 80 °C (Table 1, entry 12).

With optimized conditions in hand (Table 1, entries 9 and 12), a controlled oxidation of various glycosyl sulfides to corresponding sulfoxides and sulfones was studied with urea-hydrogen peroxide in acetic acid (Table 2). For this study, a series of α- and β-thioglycosides, **1**–**19** were initially prepared by using literature procedures (see Supporting Information File 1). In addition, structurally diverse aglycone moieties were selected in order to study the breadth and scope of the current methodology. Initially, the oxidation of *O*-acetylated and benzoylated phenyl and *p*-tolyl thioglucopyranosides was examined (Table 2, entries 1–4). These aryl sulfides underwent oxidation very efficiently to provide the corresponding sulfoxides **1a–4a** in excellent yields, i.e., 85–93% under optimized conditions. Similarly, corresponding glycosyl sulfones **1b**–**4b** were also achieved in 91–94% yield by simply altering the reaction conditions as described in the optimization study.

**Table 2:** Controlled oxidation of various thioglycosides to corresponding sulfoxides and sulfones using urea–hydrogen peroxide (UHP).^a,b^.

Entry	Substrate	Sulfoxide (**a**)^c^		Sulfone (**b**)	
		Time	Yield (%)^d^	Time	Yield (%)^d^

1	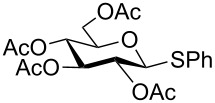 **1**	2 h	92	10 h	93
2	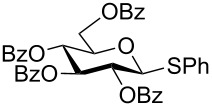 **2**	2 h	85	10 h	91
3	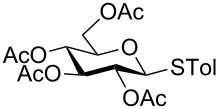 **3**	2 h	93	10 h	94
4	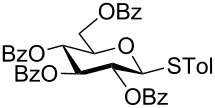 **4**	2 h	89	10 h	92
5	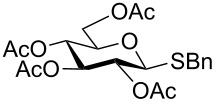 **5**	1.5 h	90	8 h	94
6	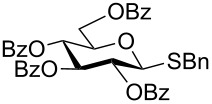 **6**	1.5 h	87	8 h	89
7	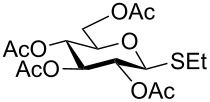 **7**	1.5 h	92	8 h	94
8	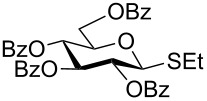 **8**	1.5 h	92	8 h	93
9	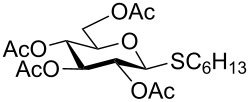 **9**	2.0 h	87	10 h	93
10	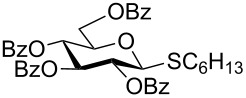 **10**	2.0 h	83	10 h	91
11	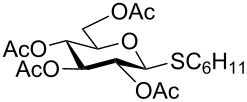 **11**	2 h	90	10 h	92
12	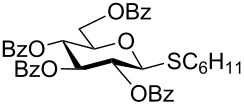 **12**	2 h	87	10 h	90
13	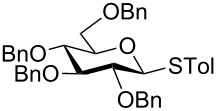 **13**	1.5 h	82	6 h	64
14	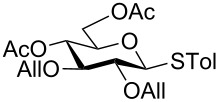 **14**	2 h	89	8 h	91
15	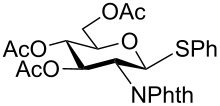 **15**	2.5 h	91	8 h	82
16	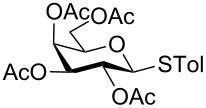 **16**	2.5 h	80	10 h	89
17	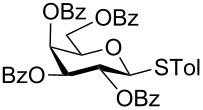 **17**	2.5 h	77	11 h	87
18	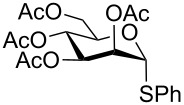 **18**	2 h	85	10 h	92
19	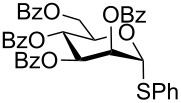 **19**	2 h	86	11 h	89

^a^Reaction Conditions: Thioglycoside (0.25 mmol), acetic acid (2.5 mL) and UHP (1.5 equiv) stirred at 60 °C. ^b^Reaction conditions: Thioglycoside (0.25 mmol), acetic acid (2.5 mL) and UHP (2.5 equiv) stirred at 80 °C. ^c^Obtained as *R* and *S* mixture. ^d^Isolated Yield.

We further examined the oxidation of *O*-acetyl- (Ac) and benzoyl- (Bz) protected benzyl thioglucopyranosides, which showed a good selectivity during the controlled oxidation with UHP and provided >87% and >89% of the desired sulfoxides (**5a** and **6a**) and sulfones (**5b** and **6b**), respectively (Table 2, entries 5 and 6). Similar to phenyl and benzyl sulfides, alkyl sulfides such as ethyl, *n*-hexyl and cyclohexyl glycosyl sulfides were also successfully oxidized in a controlled and selective manner with equal efficiency (Table 2, entries 7–12).

Having studied the oxidation of electron deficient thioglucopyranosides, we further investigated the oxidation of *O*-benzyl protected 4-methylphenyl thioglycoside **13** under optimized conditions (Table 2, entry 13). This substrate was found to be more reactive than *O*-acetylated and benzoylated thioglycosides and gave the sulfoxide in a good yield within 1.5 h. However, corresponding sulfone was obtained in a moderated yield due to instability which undergoes partial amount of decomposition.

In general, olefins functional groups are known to undergo epoxidation or dihydroxylation with different oxidizing agents (e.g. *m*-CPBA, *t*-BuOOH, oxone, etc.) [34]. Therefore, the scope of this methodology was further investigated with oxidation of allyl group protected thioglycoside **14** (Table 2, entry 14). Remarkably, allyl groups were found to be very stable during the oxidation while sulfide underwent selective oxidation to corresponding sulfoxide and sulfone in 89% and 91%, respectively. Further, we have studied the oxidation of protected glucosamine thioglycoside (Table 2, entry 15) which provided 91% of sulfoxide and 82% of sulfone.

The scope of the oxidation reaction was subsequently investigated with other monosaccharides such as galacto and mannothioglycosides under optimized conditions (Table 2). Similar to glucopyranosides, galacto and mannothioglycosides **16**–**19** were successfully oxidized to corresponding sulfoxides and sulfones in good to excellent yields (Table 2, entries 16–19). Overall, sulfoxides were achieved within the time period of 1.5–2.5 h while sulfones were obtained in 6–11 h.

## Conclusion

In conclusion, we have developed a practical method for the selective and controlled oxidation of thioglycosides to corresponding glycosyl sulfoxides and sulfones using the stable, inexpensive and commercially available oxidant urea–hydrogen peroxide (UHP). Glycosyl sulfoxides were achieved using 1.5 equiv of UHP at 60 °C while sulfones were achieved using 2.5 equiv of UHP at 80 °C. Remarkably, oxidation susceptible olefin functional groups were found to be stable during the sulfide oxidation.

## Supporting Information

File 1 Experimental part and NMR spectra.
