# Supplementary material for: Urea–hydrogen peroxide prompted the selective and controlled oxidation of thioglycosides into sulfoxides and sulfones
Source: Beilstein J Org Chem. 2017 Jun 13;13:1139–44. doi: 10.3762/bjoc.13.113 (PMC5480342; doi:10.3762/bjoc.13.113)

Supporting Information

for

Urea–hydrogen peroxide prompted the selective and controlled oxidation of thioglycosides into sulfoxides and sulfones

Adesh Kumar Singh, Varsha Tiwari, Kunj Bihari Mishra, Surabhi Gupta and Jeyakumar Kandasamy*,§

Address: Department of Chemistry, Indian Institute of Technology, Banaras Hindu University, Varanasi-221005, India

E-mail: Jeyakumar Kandasamy - jeyakumar.chy@iitbhu.ac.in

*Corresponding author
§Tel.: +91-0542-6702879; fax: +91- 0542-6702876;

Experimental part and NMR spectra

LIST OF CONTENTS:

1) General information …………………………………………………………………….. 01

2) Experimental procedure for the preparation of thioglycosides…………………………..…. 02

3) Experimental procedure for the oxidation of thioglycosides……………………………..… 10

4) Analytical data for the glycosyl sulfoxides………………………………………………….. 11

5) Analytical data for the glycosyl sulfones…………………………………………….…… 20

6) References………………………………………………………………………………….. 28

7) ^{1}H and ^{13}C spectra of the products ………………………………………………………… 30

1. General information

Starting materials were prepared using literature procedures or modified procedures as stated below. All the reactions were performed in round bottom flask as described below. Solvents and chemicals were purchased from commercial sources and used without further purification. The oxidant, urea–hydrogen peroxide (97%) was purchased

Sigma-Aldrich. Thin layer chromatography was performed using pre-coated plates contained from E. Merck (TLC silica gel 60 F254). TLC plates were visualized by exposure to ultraviolet light (UV), then further analyzed by charring in stain solution (5% H_2SO_4 in MeOH). The column chromatography was performed on silica gel (100–200 mesh) using a mixture of ethyl acetate and hexane as an eluent. The NMR spectra were recorded on Bruker Avance 500 MHz NMR spectrometer and Mass spectra were measured on water's Quattro Micro V 4.1. The ^{1}H NMR and ^{13}C NMRs of the known glycosyl sulfide, glycosyl sulfoxide and glycosyl sulfone were compared with literature reports.

2. Experimental Procedure for the synthesis of thiogycosides

2.1 Preparation of alkyl or aryl 2,3,4,6-tetra-O-acetyl-1-thio-β-D-glucopyranoside [1]

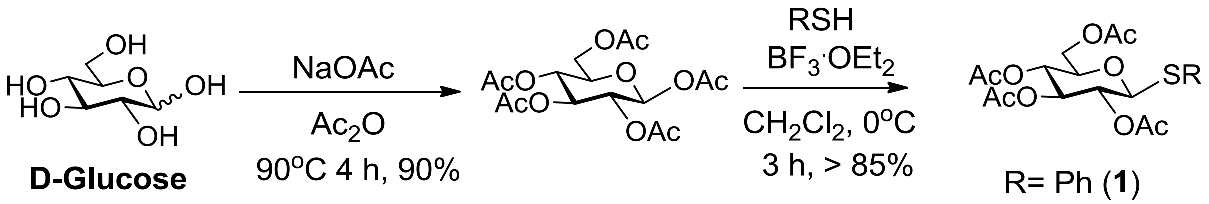

R= Ph (**1**)
R= Tol (**3**)
R= CH_2Ph (**5**)
R= Et (**7**)
R= n-Hexyl(**9**)
R = Cyclohexyl(**11**)

Sodium acetate (13.66 g, 166.66 mmol) and acetic anhydride (86 mL, 916 mmol) was refluxed for 20 minutes at 90 °C to which D-glucose (10.0 g, 55.51 mmol) was added in three portions over a period of 10 min. The reaction mixture was further refluxed for 4 h at 90 °C. After completion (checked by TLC), the reaction mixture was cooled to room temperature and then poured into the beaker containing crushed ice (250 mL) under stirring conditions. The penta-acetate was precipitated. The precipitate was filtered and washed with ice-cold water until the odor of the acetic acid was removed. The crude product was purified by recrystallization from MeOH to afford the β-D-per-acetylated glucose in 90% (19.5 g) as a white crystalline solid.

1,2,3,4,6-Penta-O-acetyl-β-D-glucopyranoside (5 g)and alkyl or aryl thiol (1.2 equiv) was stirred in dry CH_2Cl_2 at 0 °C under N_2 atmosphere to which $BF_3 \cdot OEt_2$ (2.3 equiv) added drop-wise. After completion (approx. 3 hours), the reaction mixture was neutralized with TEA. Further, the reaction mixture was diluted with DCM and washed with $NaHCO_3$followed by brine solution. The organic extract was evaporated in vacuum and the crude residue was purified by column chromatography (hexane:EtOAc) to obtain the corresponding product in >85% yield.

<u>**2.2** Preparation of alkyl or aryl 2,3,4,6-tetra-O-benzoyl-1-thio-β-D-glucopyranoside </u>[2]

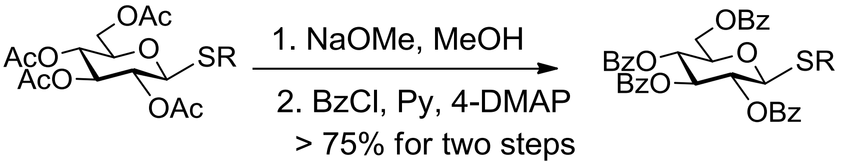

R= Ph (**1**)
R= Tol (**3**)
R= CH_2Ph (**5**)
R= Et (**7**)
R= n-Hexyl (**9**)
R = Cyclohexyl(**11**)

R= Ph (**2**)
R= Tol (**4**)
R= CH_2Ph (**6**)
R= Et (**8**)
R= n-Hexyl (**10**)
R =Cyclohexyl(**12**)

To a stirred solution of tetra-acetylthioglycoside (2.5 g) in dry MeOH, a catalytic amount of NaOMe (31.33 mg) was added and stirred overnight. After completion, the reaction mixture was neutralized with AMBERLITE® resin IR-120 (H^+ form) and filtered. The solvent was evaporated and dried in high vacuum. The crude tetraol was stirred in pyridine at 0 °C to which BzCl was added drop-wise followed by a catalytic amount of DMAP. The reaction mixture was stirred overnight and concentrated in rota-evaporator. Further, the crude residue was diluted with H_2O and extracted with EtOAc (3 × 20 mL). The combined organic layers were washed with 0.1 N HCl solution followed by brine and dried over anhydrous Na_2SO_4. Finally, the organic layer was concentrated and purified by column chromatography (hexane:EtOAc) to obtain the title compounds in >75% yield over two steps.

2.3 Preparation of 4-methyl phenyl 2,3,4,6-tetra-O-benzyl-1-thio-*β*-D-glucopyranoside [3]

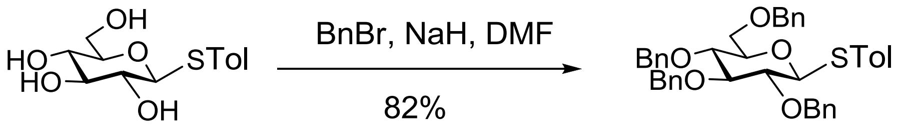

13

The tetraol (2.5 g, 9.18 mmol) was stirred in dry DMF (20 mL) to which NaH (1.32 g, 55.08 mmol) was added portion wise at 0 °C under nitrogen atmosphere. After 15 min, BnBr (5.5 ml, 45.9mmol) was added drop wise and allowed to stir the reaction mixture for additional 15 h at room temperature. The reaction mixture was carefully quenched with cooled water and concentrated in vacuo. The crude residue was diluted with H_2O and extracted with EtOAc (3 × 20 mL). The combined organic layer was washed with brine, dried over Na_2SO_4 and concentrated in vacuo. The residue was purified by flash chromatography (hexane:EtOAc) to obtain 4-methylphenyl 2,3,4,6-tetra-*O*-benzyl 1-thio-β-D-glucopyranoside (**13**) in (4.8 g, 82%).

2.4 Preparation of 4-methylphenyl 2,3-di-O-allyl-4,6-di-*O*-acetyl-1-thio-β-D-glucopyranoside. [3]

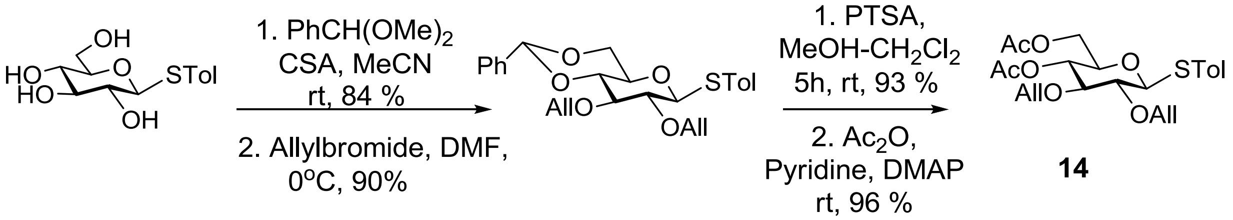

14

To a suspension of tetraol (2.0 g, 6.98 mmol) and camphorsulfonic acid (pH = 2) (0.48 g, 2.10 mmol) in acetonitrile (30 ml), benzaldehyde dimethylacetal (1.53 mL, 10.48 mmol) was added in drop-wise at room temperature and stirred overnight. After completion, the reaction mixture was neutralized with TEA and concentrated in vacuo. The crude residue was purified by flash chromatography (hexane:AcOEt) to obtain 4-methylphenyl 4,6-O-benzylidene-1-thio-β-D-glucopyranosideas a product (2.20 g, 84%)

To a solution of 4-methylphenyl 4,6-di-*O*-benzylidene-1-thio-β-D-glucopyranoside (1.0 g, 2.67 mmol) in dry DMF (20 mL), NaH (256.35 mg, 10.68 mmol) was added portion wise

at 0 °C under nitrogen atmosphere. After 15 min, allyl bromide (0.5 ml, 5.88 mmol) was added drop wise and allowed to stir the reaction mixture for additional 15 h at room temperature. The reaction mixture was carefully quenched with cooled water and concentrated in vacuo. The crude residue was diluted with H_2O and extracted with EtOAc (3 × 20 mL). The combined organic layer was washed with brine, dried over Na_2SO_4 and concentrated in vacuo. The residue was purified by flash chromatography (hexane:EtOAc) to obtain 4-methylphenyl 2,3-di-O-allyl-4,6-O-benzylidene-1-thio-β-D-glucopyranosideas a viscous oil (1.10 g, 90.61%).

The above product (1.0 g, 2.2 mmol) and *p*-TsOH (189.41 mg, 1.10 mmol) was stirred in a mixture of MeOH (15.0 mL) and CH_2Cl_2 (3.0 mL) for 6 h at 25 °C. After completion, the reaction mixture was quenched with Et_3N and evaporated in vacuo then diluted with dichloromethane. Further, the organic layer was washed with water, dried over anhydrous Na_2SO_4 and concentrated under reduced pressure. The crude product was purified by flash column chromatography (silica gel, EtOAc:hexanes) to give 4-methylphenyl 2,3-di-O-allyl-1-thio-β-D-glucopyranoside as a white solid (0.750 g, 93% yield). This diol compound was stirred in pyridine (10 mL) at 0 °C to which Ac_2O (0.67 ml, 7.13 mmol) added drop-wise followed by a catalytic amount of DMAP. The reaction mixture was allowed to stir at room temperature for 6 h. After completion, the reaction mixture was concentrated in vacuo and diluted with EtOAc. The organic layer was washed with washed with, 0.1 N HCl solution, brine and dried over Na_2SO_4. The organic layer was concentrated and purified by column chromatography (hexane:EtOAc) to obtain the title compound (**14**) as a viscous oil (0.60 g, 96%). ^{1}H NMR (500 MHz, $CDCl_3$): δ 7.45-7.43 (m, 2H), 7.10-7.09 (m, 2H), 6.01-5.93 (m, 1H), 5.87-5.79 (m, 1H), 5.31-5.12(m, 4H), 4.94-4.90 (m, 1H), 4.50-4.48 (m, 1H), 4.35- 4.17 (m, 4H), 4.11-4.08 (m, 2H), 3.55-3.52 (m, 1H), 3.48-3.44 (m, 1H), 3.33-3.29 (m, 1H), 2.33 (s, 3H), 2.11 – 2.06 (s, 6H). ^{13}C NMR (125 MHz, $CDCl_3$): δ 170.4, 169.3, 137.8, 134.4, 134.4, 132.7, 129.4, 129.1, 117.3, 116.8, 87.5, 83.3, 79.8, 75.6, 74.1, 74.0, 69.5, 62.5, 20.9, 20.7, 20.6.

2.5 Preparation of phenyl 3,4,6-tri-*O*-acetyl-2-deoxy-2-phthalimido-1-thio-β-D-glucopyranoside (**15**) [4]

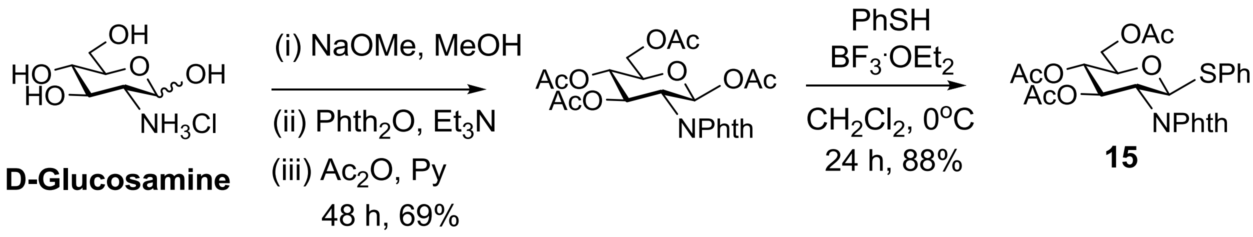

Glucosamine hydrochloride (5 g, 23.30 mmol) was mechanically agitated with 1 M NaOMe in MeOH (25 mL) for 2 h at room temperature, followed by addition of finely powdered phthalic anhydride (1.9 g, 12.8 mmol) and Et_3N (3.5 mL, 2.5 mmol). The mixture was stirred for 45 min, then treated with a second portion of phthalic anhydride (1.9 g, 12.8 mmol) and stirred for 24 h at room temperature. The mixture was cooled to −20 °C for 4 h, filtered, washed with cold MeOH, and dried under reduced pressure to yield a light yellow solid. The solid was suspended in pyridine (50 mL) to which Ac_2O (33.0 mL) was added at 0 °C and stirred for 48 h at room temperature. The crude was concentrated and purified by recrystallization (in EtOH) to afford 1,3,4,6-tetra-*O*-acetyl-2-phthalimido-2-deoxy-β-D-glucopyranoside as an amorphous white solid (7.6 g, 69% over 3 steps).

The above tetraacetate (2.87 g, 6.0 mmol) and thiophenol (0.73 mL, 7.20 mmol) was stirred in CH_2Cl_2 (20 mL) at 0 °C to which $BF_3 \cdot Et_2O$ (2 mL) was added drop wise. The resulted mixture was stirred for 24 h at room temperature and quenched with TEA. Further, the reaction mixture was diluted with CH_2Cl_2 (3 × 20 mL) and washed with saturated $NaHCO_3$ and brine, then dried over Na_2SO_4. The organic layer was concentrated and purified by column chromatography to afford phenyl 3,4,6-tri-O-acetyl-2-deoxy-2-phthalimido-β-D-glucopyranoside (**15**) as an amorphous white solid in 88% yield (2.8 g, 5.31 mmol).

2.6 Preparation of *p*-tolyl2,3,4,6-tetra-O-acetyl-1-thio-β-D-galactopyranose and *p*-tolyl 2,3,4,6-tetra-*O*-benzoyl-1-thio β-D-galactopyranose [5]

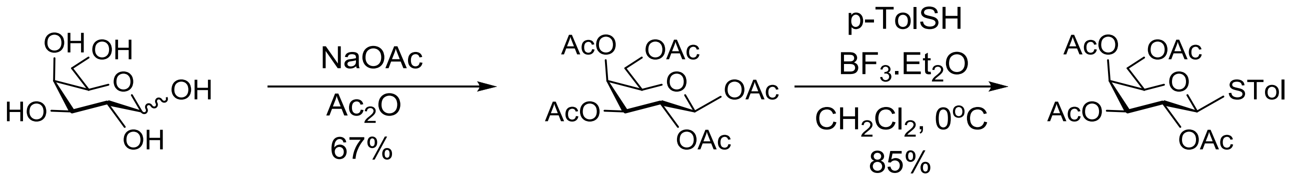

R = *p*-Tol (**16**)

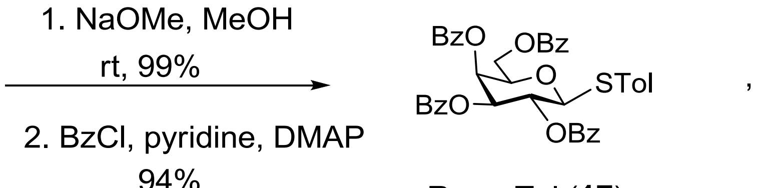

R = *p*-Tol (**17**)

To a stirred solution of D-galactose (10 g, 55.5 mmol) in acetic anhydride (52.5 mL), NaOAc (2.28 g, 27.7 mmol) was added and the solution was heated at 60 °C. After completion (approx. 6 h), the reaction mixture was cooled to room temperature anddilutedwith DCM and washed with saturated $NaHCO_3$. The organic layer was dried over anhydrous Na_2SO_4, concentrated and purified using column chromatography(ethyl acetate/ petroleum ether) to obtain the title compound in 67% yield (14.5 g, 37.1 mmol).

1,2,3,4,6-Penta-*O*-acetyl-β-D-galactopyranose (5.00 g, 12.81 mmol) and 4-methylthiophenol (1.77 mL, 15.27 mmol) was stirred in dry CH_2Cl_2 at 0 °C under N_2 atmosphere to which $BF_3{\cdot}OEt_2$ (2.3 equiv) was added drop-wise. After completion (approx. 3 hours), the reaction mixture was neutralized with TEA. Further, the reaction mixture was diluted with DCM and washed with $NaHCO_3$ followed by brine solution. The organic extract was evaporated in vacuum and the crude residue was purified by flash chromatography (hexane:EtOAc) to obtain *p*-tolyl-2,3,4,6-tetra-*O*-acetyl-1-thio-β-D-galactopyranose (**16**) (4.8 g, 10.90mmol) 85% yield.

To a stirred solution of **16** (5.0 g, 11.1 mmol) in dry MeOH, a catalytic amount of NaOMe (0.059 g, 1.1 mmol) was added and stirred for overnight. After completion, the reaction mixture was neutralized with AMBERLITE® resin IR-120 (H^+ form) and filtered. The solvent was evaporated and dried in high vacuum. The crude tetraol was stirred in pyridine at 0 °C to which BzCl (7.41 mL, 63.7 mmol) was added drop wise followed by

catalytic amount of DMAP. The reaction mixture was stirred for overnight and concentrated in rota-evaporator. Further, the crude residue was diluted with H_2O and extracted with EtOAc (3 × 20 mL). The combined organic layers were washed with 0.1 N HCl solution followed by brine and dried over anhydrous Na_2SO_4. Finally, the organic layer was concentrated and purified by column chromatography (hexane:EtOAc) to obtain *p*-tolyl 2,3,4,6-tetra-*O*-benzoyl-1-thio-β-D-galactopyranose (**17**) (4.9 g, 9.9 mmol) 94% yield.

2.7 Preparation of phenyl 2,3,4,6-tetra-O-acetyl-1-thio-α-D-mannopyranose and phenyl 2,3,4,6-tetra-O-benzoyl-1-thio-α-D-mannopyranose [5]

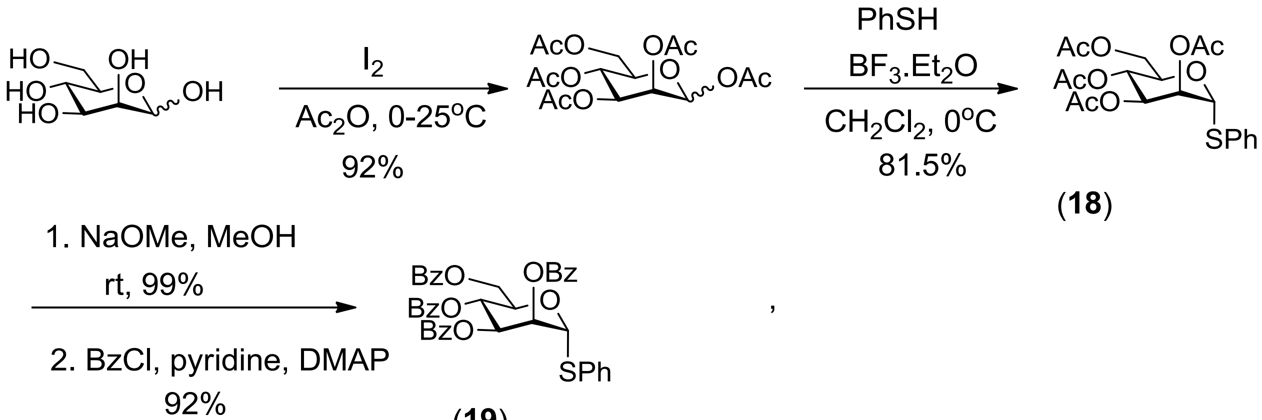

(**18**)

(**19**)

D-Mannose (10.0 g, 55.5 mmol, 1.0 equiv) was slowly added to a solution of iodine (I_2, 0.28 g, 1.1 mmol, 0.02 equiv) in Ac_2O (25 mL) at 0 °C and under nitrogen atmosphere. After stirring 30 min at 0 °C, the reaction was allowed to stir for additional 16 h at room temperature. After completion, the reaction mixture was diluted with 60 mL of CH_2Cl_2 and washed with a cold saturated $Na_2S_2O_3$solution (2 × 60 mL) followed by saturated $NaHCO_3$ solution of (4 × 35 mL). The organic layer was dried over anhydrous Na_2SO_4 and evaporated to dryness. The crude residue was purified by column chromatography to afford the desired peracetylated compound (20.0 g, 92%, mixture of both anomers).

1,2,3,4,6-Penta-*O*-acetyl-D-mannopyranose (5.0 g, 12.8 mmol) and thiophenol (1.57 mL, 15.3 mmol) was stirred in dry CH_2Cl_2 at 0 °C under N_2 atmosphere to which

$BF_3{\cdot}OEt_2$ (2.3 equiv) was added drop wise. After completion (approx. 3 hours), the reaction mixture was neutralized with TEA. Further, the reaction mixture was diluted with DCM and washed with $NaHCO_3$ followed by brine solution. The organic extract was evaporated in vacuum and the crude residue was purified by flash chromatography (hexane:EtOAc) to obtain phenyl 2,3,4,6-tetra-*O*-acetyl-1-thio-α-D-mannopyranose (**18**) 82% yield (4.6 g, 10.44 mmol).

To a stirred solution of **18** (4.0 g, 9.08 mmol) in dry MeOH (30 mL), a catalytic amount of NaOMe (49.06 mg, 0.9 mmol) was added and stirred overnight. After completion, the reaction mixture was neutralized with AMBERLITE® resin IR-120 (H^+ form) and filtered. The solvent was evaporated and dried in high vacuum. The crude tetraol was stirred in pyridine (30 mL) at 0 °C to which BzCl (5.63 mL, 48.47 mmol) was added drop-wise followed by catalytic amount of DMAP. The reaction mixture was stirred overnight and concentrated in a rota-evaporator. Further, the crude residue was diluted with H_2O and extracted with EtOAc (3 × 20 mL). The combined organic layers were washed with 0.1 N HCl solution followed by brine and dried over anhydrous Na_2SO_4. Finally, the organic layer was concentrated and purified by column chromatography (hexane:EtOAc) to obtain phenyl 2,3,4,6-tetra-*O*-benzoyl-1-thio-α-D-mannopyranose (**19**) in 92% yield (5.1 g, 7.48 mmol).

Note: The following glycosyl sulfides are known in the literature: **1**, **3**, **5**, **7**, **11**, **15**, **18** [6], **2** [7], **4**, **13** [2], **6** [8], **8** [9], **9** [10], **10**, **12** [11], **16** [12], **17** [13], **19** [14].

3.0 Experimental procedure for the oxidation of thioglycosides

3.1 General procedure for the oxidation of thioglycoside to corresponding glycosyl sulfoxides with urea–hydrogen peroxide

Glycosyl sulfide (0.25 mmol) and urea hydrogen peroxide (1.5 equiv) was stirred in acetic acid (2.5 mL) at room temperature for 5 minutes and heated at 60 °C for 1.5–2.5 hour (as per Table 2 in the manuscript). After completion, the reaction mixture was cooled to room temperature and diluted with EtOAc (30 ml). The organic layer was washed with saturated $NaHCO_3$ followed by brine solution and dried over anhydrous Na_2SO_4. Further, the organic layer was evaporated and purified in column chromatography to obtain the corresponding glycosyl sulfoxides.

Note: The following glycosyl sulfoxides are known in the literature: **1a**, **2a** [15], **3a**, **4a**, **7a**, **13a,16a, 18a** [16], **8a** [17], **11a** [18], **15a** [19],**17a** [20], **19a** [14].

3.2 General procedure for the oxidation of thioglycosides to corresponding glycosyl sulfones with urea–hydrogen peroxide

Glycosyl sulfide (0.25 mmol) and urea hydrogen peroxide (2.5 equiv) was stirred in acetic acid (2.5 mL) at room temperature for 5 minutes and heated at 80 °C for 6–11 hours (as per Table 2 in manuscript). After completion, the reaction mixture was cooled to room temperature and diluted with EtOAc (30 mL). The organic layer was washed with saturated $NaHCO_3$ followed by brine solution and dried over anhydrous Na_2SO_4. Further, the organic layer was evaporated to dryness and the crude residue was purified by column chromatography to obtain the corresponding glycosyl sulfones.

Note: The following glycosyl sulfones are known in the literature: **1b, 2b, 7b, 15b** [21], **3b, 16b** [22], **5b, 11b** [23], **18b** [24].

4.0 Analytical data for the glycosyl sulfoxides

4.1 1-Deoxy-1-[(*R*/*S*)-(phenyl)sulfinyl]-2,3,4,6-tetra-*O*-acetyl-β-D-glucopyranose (1a) was obtained as a white solid (yield = 104 mg, 92%). ^{1}H NMR (500 MHz, $CDCl_3$) δ 7.62-7.57 (m, 2H), 7.47-7.46 (m, 3H), 5.27-5.16 (m, 2H), 4.95-4.87 (m, 1H), 4.38-4.20 (m, 1H), 4.09-3.94 (m, 2H), 3.65-3.52 (m, 1H), 2.00-1.84 (m, 12H). ^{13}C NMR (125 MHz, $CDCl_3$) δ 170.3, 170.1, 169.3, 169.2, 138.9, 131.6, 128.8, 125.7, 92.2, 89.9, 76.5, 76.3, 73.8, 73.5, 67.7, 67.4, 61.7, 61.3, 20.6, 20.5, 20.5, 20.4. HRMS: Calc. for $C_{20}H_{25}O_{10}S$ $[M+H]^+$: 457.1168, Obser. 457.1177.

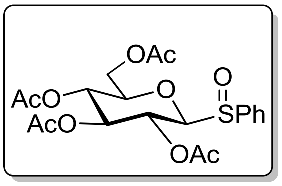

4.2 1-Deoxy-1-[(*R*/*S*)-(phenyl)sulfinyl]-2,3,4,6-tetra-*O*-benzoyl-β-D-glucopyranose (2a) was obtained as a white solid (yield = 149 mg, 85%). ^{1}H NMR (500 MHz, $CDCl_3$) δ 7.90-7.56 (m, 10H), 7.48-7.24 (m, 11H), 7.21-7.16 (m, 4H), 5.94-5.6m, 2H), 5.54-5.43 (m, 1H), 4.78 (d, *J* = 10.0 Hz, 1H), 4.58-4.51 (m, 1H), 4.46-4.30 (m, 1H), 4.14-4.02 (m, 1H). ^{13}C NMR (125 MHz, $CDCl_3$) δ 165.7, 165.7, 165.7, 165.6, 165.0, 164.8, 164.8, 164.6, 138.6, 138.2, 133.4, 133.4, 133.3, 133.1, 133.1, 131.4, 131.4, 129.9, 129.8, 129.7, 129.7, 129.6, 129.3, 129.2, 128.8, 128.7, 128.5, 128.4, 128.4, 128.4, 128.3, 128.3, 128.3, 128.2, 128.2, 125.7, 125.4, 92.9, 90.7, 77.2, 77.0, 73.8, 73.7, 68.9, 68.3, 68.1, 67.9, 62.8, 62.1. HRMS: Calc. for $C_{40}H_{32}O_{10}SNa$ $[M+Na]^+$: 727.1614, Obser. 727.1597.

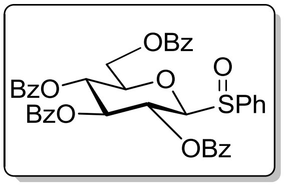

4.3 1-Deoxy-1-[(*R*/*S*)-(4-methylphenyl)sulfinyl]-2,3,4,6-tetra-*O*-acetyl-β-D-glucopyranose (3a) was obtained as a white solid (yield = 109 mg, 93%). ^{1}H NMR (500 MHz, $CDCl_3$) δ 7.48 (dd, *J* = 15.5, 8.5 Hz, 2H), 7.26 (d, *J* = 7.5 Hz, 2H), 5.25-5.11 (m, 2H), 4.95-4.85 (m, 1H), 4.35-4.19 (m, 1H), 4.10-3.96 (m, 2H), 3.64-3.53 (m, 1H), 2.35

(s, 3H), 1.98-1.87 (m, 12H). ^{13}C NMR (125 MHz, $CDCl_3$) δ 170.3, 170.3, 170.1, 169.3, 169.2, 169.1, 168.9, 142.3, 142.2, 135.4, 135.2, 129.6, 129.5, 125.9, 125.8, 92.0, 89.7, 76.4, 76.2, 73.8, 73.5, 67.7, 67.6, 67.4, 61.7, 61.3, 21.4, 21.4, 20.5, 20.5, 20.4. HRMS: Calc. for $C_{21}H_{26}O_{10}SNa$ [M+Na]$^+$: 493.1144, Obser. 493.1153.

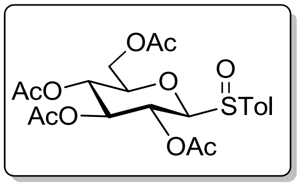

4.4 1-Deoxy-1-[(*R*/*S*)-(4-methylphenyl)sulfinyl]-2,3,4,6-tetra-*O*-benzoyl-β-D-glucopyranose (4a) was obtained as a white solid (yield = 159 mg, 89%). ^1H NMR (500 MHz, $CDCl_3$) δ 7.88-7.70 (m, 8H), 7.52-7.19 (m, 14H), 7.05-7.01 (m, 2H), 5.91-5.83 (m, 1H), 5.81-5.44 (m, 2H), 4.79-4.59 (m, 1H), 4.51-4.46 (m, 1H), 4.41-4.31 (m, 1H), 4.14-4.05 (m, 1H), 2.13-2.08 (m, 3H). ^{13}C NMR (125 MHz, $CDCl_3$) δ 165.8, 165.8, 165.7, 165.6, 165.0, 164.9, 164.8, 164.7, 142.2, 135.5, 134.7, 133.5, 133.4, 133.3, 133.1, 129.9, 129.8, 129.8, 129.7, 129.7, 129.7, 129.6, 129.5, 129.4, 128.5, 128.5, 128.3, 128.3, 128.3, 128.2, 125.9, 125.7, 92.7, 90.6, 73.9, 73.8, 68.9, 68.4, 68.1, 67.8, 62.9, 62.2, 21.3, 21.2. HRMS: Calc. for $C_{41}H_{34}O_{10}SNa$ [M+Na]$^+$: 741.1770, Obser. 741.1782.

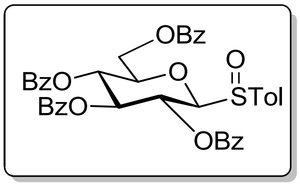

4.5 1-Deoxy-1-[(*R*/*S*)-(benzyl)sulfinyl]-2,3,4,6-tetra-*O*-acetyl-β-D-glucopyranose (5a) was obtained as a white solid (yield = 105 mg, 90%). ^1H NMR (500 MHz, $CDCl_3$) δ 7.41-7.35 (m, 5H), 5.48-5.22 (m, 2H), 5.15-5.10 (m, 1H), 4.44-4.25 (m, 3H), 4.19-4.08 (m, 2H), 3.86-3.77 (m, 1H), 2.18-2.02 (m, 12H). ^{13}C NMR (125 MHz, $CDCl_3$) δ 170.4, 169.9, 169.6, 169.3, 169.1, 168.5, 130.5, 129.7, 129.3, 129.1, 128.8, 128.7, 128.5, 88.7, 84.4, 76.9, 73.7, 73.0, 68.3, 67.9, 67.7, 66.3, 62.4, 61.5, 53.6, 53.3, 20.7, 20.7, 20.5, 20.5, 20.5, 20.4.HRMS: Calc. for $C_{21}H_{27}O_{10}S$ [M+H]$^+$: 471.1325, Obser. 471.1322.

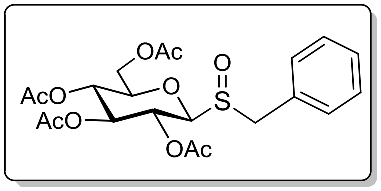

4.6 1-Deoxy-1-[(*R*/*S*)-(benzyl)sulfinyl]-2,3,4,6-tetra-*O*-benzoyl-β-D-glucopyranose (6a) was obtained as a white solid (yield = 156 mg, 87%). ^{1}H NMR (500 MHz, $CDCl_3$) δ 8.08-8.06 (m, 2H), 7.94-7.79 (m, 6H), 7.57-7.27 (m, 17H), 6.01-5.65 (m, 3H), 4.82-4.77 (m, 1H), 4.68-4.48 (m, 2H), 4.34-4.24 (m, 2H), 4.17-4.09 (m, 1H). ^{13}C NMR (125 MHz, $CDCl_3$) δ 166.1, 166.0, 165.7, 165.5, 165.3, 165.1, 165.0, 164.4, 133.6, 133.5, 133.3, 133.3, 130.5, 129.9, 129.8, 129.8, 129.8, 129.7, 129.7, 129.6, 129.2, 128.8, 128.6, 128.4, 128.4, 128.4, 128.3, 128.3, 128.2, 89.7, 84.9, 77.3, 77.2, 73.7, 73.30, 69.2, 69.1, 68.6, 66.9, 63.6, 62.4, 60.3, 53.5, 53.4.HRMS: Calc. for $C_{41}H_{35}O_{10}S$ $[M+H]^+$: 719.1951, Obser. 719.1955.

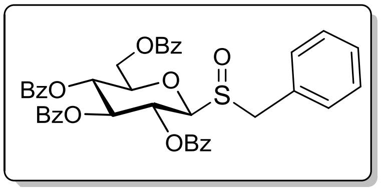

4.7 1-Deoxy-1-[(*R*/*S*)-ethylsulfinyl]-2,3,4,6-*O*-acetyl-β-D-glucopyranose (7a) was obtained as a white solid (yield = 93 mg, 92%). ^{1}H NMR (500 MHz, $CDCl_3$) δ 5.53-5.18 (m, 2H), 5.03-4.93 (m, 1H), 4.74 (d, *J* = 5.0 Hz, 1H), 4.28-4.01 (m, 2H), 3.87-3.73 (m, 1H), 2.87-2.71 (m, 2H), 2.04-1.97 (m, 12H), 1.35-1.30 (m, 3H). ^{13}C NMR (125 MHz, $CDCl_3$) δ 170.3, 170.2, 169.9, 169.8, 169.6, 169.3, 169.2, 89.8, 87.1, 76.8, 73.5, 73.1, 69.8, 68.7, 68.4, 67.9, 67.6, 61.8, 61.4, 42.4, 41.2, 20.5, 20.5, 20.5, 20.5, 20.4, 6.4, 5.6. HRMS: Calc. for $C_{16}H_{25}O_{10}S$ $[M+H]^+$: 409.1168, Obser. 409.1158.

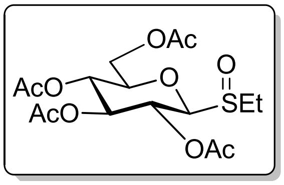

4.8 1-Deoxy-1-[(*R*/*S*)-(ethyl)sulfinyl]-2,3,4,6-tetra-*O*-benzoyl-β-D-glucopyranose (8a) was obtained as a white solid (yield = 151 mg, 92%). ^{1}H NMR (500 MHz, $CDCl_3$) δ 8.04-7.98 (m, 2H), 7.96-7.94 (m, 2H), 7.92-7.89 (m, 2H), 7.84-7.82 (m, 2H), 7.58-7.48 (m, 3H), 7.44-7.26 (m, 9H), 6.11-5.67 (m, 3H), 4.72-4.57 (m, 2H), 4.54-4.49 (m, 1H), 4.34-4.25 (m, 1H), 3.21-2.85 (m, 2H), 1.38-1.25 (m, 3H). ^{13}C NMR (125 MHz, $CDCl_3$) δ 165.9, 165.8, 165.5, 165.3, 165.0, 165.0, 164.5, 133.6, 133.5, 133.4, 133.3, 133.2, 129.9, 129.8, 129.7, 129.7, 129.6, 129.6, 129.3, 128.5, 128.4, 128.3, 128.3, 90.6, 87.1, 73.8, 73.3, 69.3, 69.1, 68.7, 67.5, 63.3, 62.4, 41.3, 41.1, 7.3, 6.5.HRMS: Calc. for $C_{36}H_{32}O_{10}SNa$ $[M+Na]^+$: 679.1614, Obser. 679.1620.

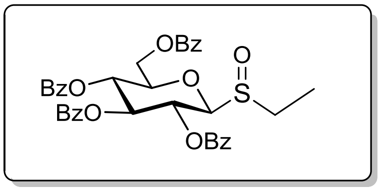

4.9 1-Deoxy-1-[(*R*/*S*)-(n-hexyl)sulfinyl]-2,3,4,6-tetra-O-acetyl-*β*-D-glucopyranose (9a) was obtained as a white solid (yield = 101 mg, 87%). ^{1}H NMR (500 MHz, $CDCl_3$) δ 5.45-5.21 (m, 2H), 5.16-5.08 (m, 1H), 4.36-4.16 (m, 3H), 3.82-3.79 (m, 1H), 3.16-2.91 (m, 1H), 2.83-2.67 (m, 1H), 2.09-2.02 (m, 12H), 1.82-1.78 (m, 1H), 1.70-1.66 (m, 1H), 1.50-1.45 (m, 2H), 1.33-1.32 (m, 4H), 0.91-0.88 (m, 3H). ^{13}C NMR (125 MHz, $CDCl_3$) δ 170.4, 169.9, 169.6, 169.3, 169.1, 168.7, 90.2, 86.8, 76.8, 76.8, 73.7, 73.2, 68.3, 67.8, 67.6, 66.8, 61.9, 61.4, 47.4, 47.2, 31.3, 28.5, 28.4, 22.7, 22.3, 22.0, 20.6, 20.5, 20.5, 20.5, 13.9.HRMS: Calc. for $C_{20}H_{32}O_{10}SNa$ $[M+Na]^+$: 487.1614, Obser. 487.1611.

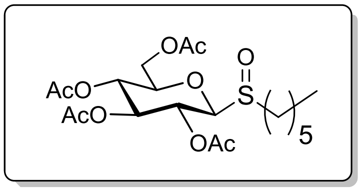

4.10 1-Deoxy-1-[(*R*/*S*)-(n-hexyl)sulfinyl]-2,3,4,6-tetra-*O*-benzoyl-β-D-glucopyranose (10a) was obtained as a white solid (yield = 147 mg, 83%). ^{1}H NMR (500 MHz, $CDCl_3$) δ 8.04-7.82 (m, 8H), 7.58-7.26 (m, 12H), 6.09-5.66 (m, 3H), 4.74-4.46 (m, 3H), 4.35-4.26 (m, 1H), 3.24-2.99 (m, 1H), 2.92-2.72 (m, 1H), 1.82-1.73 (m, 2H), 1.41-1.32 (m, 2H),

1.27-1.22 (m, 4H), 0.87-0.82 (m, 3H). ^{13}C NMR (125 MHz, $CDCl_3$) δ 165.9, 165.8, 165.8, 165.5, 165.3, 165.1, 165.0, 164.5, 133.6, 133.5, 133.4, 133.3, 133.3, 133.2, 129.9, 129.8, 129.7, 129.7, 129.6, 129.6, 129.3, 128.6, 128.5, 128.4, 128.4, 128.3, 128.3, 90.9, 87.4, 77.1, 73.7, 73.3, 69.3, 69.0, 68.7, 67.5, 63.3, 62.3, 47.4, 47.1, 31.2, 28.5, 28.4, 22.7, 22.35, 22.2, 22.1, 13.8, 13.8.HRMS: Calc. for $C_{40}H_{40}O_{10}SNa$ $[M+Na]^+$: 735.2240, Obser. 735.2245.

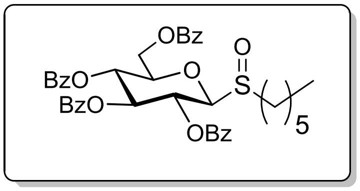

4.11 1-Deoxy-1-[(*R/S*)-(cyclohexyl)sulfinyl]-2,3,4,6-tetra-*O*-acetyl-β-D-glucopyranose (11a) was obtained as a white solid (yield = 104 mg, 90%). ^{1}H NMR (500 MHz, $CDCl_3$) δ 5.48-5.27 (m, 2H), 5.12-5.06 (m, 1H), 4.33-4.30 (m, 1H), 4.22-4.18 (m, 2H), 3.81-3.76 (m, 1H), 3.28-2.9 (m, 1H), 2.08-2.02 (m, 12H), 1.95-1.92 (m, 3H), 1.72-1.55 (m, 3H), 1.39-1.24 (m, 4H). ^{13}C NMR (125 MHz, $CDCl_3$) δ 170.4, 170.3, 170.3, 170.0, 169.4, 169.2, 169.1, 168.6, 88.2, 85.2, 77.0, 73.8, 73.2, 68.7, 67.9, 67.7, 66.7, 62.2, 61.6, 55.9, 55.2, 26.7, 26.5, 25.7, 25.6, 25.4, 25.3, 25.2, 25.1, 25.0, 23.2, 20.6, 20.6, 20.5, 20.4.HRMS: Calc. for $C_{20}H_{31}O_{10}S$ $[M+H]^+$: 463.1638, Obser. 463.1642.

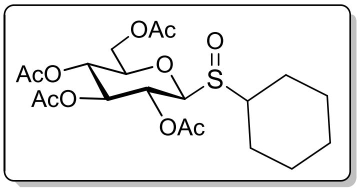

4.12 1-Deoxy-1-[(*R/S*)-(cyclohexyl)sulfinyl]-2,3,4,6-tetra-*O*-benzoyl-β-D-glucopyranose (12a) was obtained as a white solid (yield = 154 mg, 87%). ^{1}H NMR (500 MHz, $CDCl_3$) δ 8.05-8.00 (m, 2H), 7.96-7.90 (m, 4H), 7.85-7.83 (m, 2H), 7.59-7.27 (m, 12H), 6.12-5.93 (m, 2H), 5.70-5.61 (m, 1H), 4.70-4.64 (m, 2H), 4.60-4.49 (m, 1H), 4.36-4.25 (m, 1H), 3.38-2.99 (m, 1H), 1.94-1.92 (m, 1H), 1.81-1.76 (m, 1H), 1.61-1.55 (m, 3H), 1.31-1.14 (m, 5H). ^{13}C NMR (125 MHz, $CDCl_3$) δ 165.8, 165.8, 165.6, 165.1, 165.1, 165.0, 164.4, 133.6, 133.5, 133.4, 133.3, 133.3, 133.2, 129.8, 129.8, 129.7,

129.7, 129.6, 129.6, 129.3, 129.3, 128.7, 128.5, 128.4, 128.3, 128.3, 89.2, 85.5, 77.3, 77.1, 73.9, 73.4, 69.4, 69.3, 68.7, 67.4, 63.5, 62.6, 55.7, 55.1, 26.8, 26.6, 25.8, 25.5, 25.3, 25.2, 24.9, 24.8, 23.3.HRMS: Calc. for $C_{40}H_{38}O_{10}SNa$ $[M+Na]^+$: 733.2083, Obser. 733.2088.

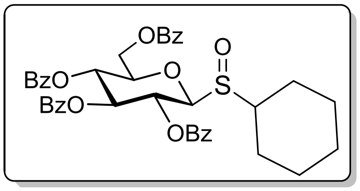

4.13 1-Deoxy-1-[(*R*/*S*)-(4-methylphenyl)sulfinyl]-2,3,4,6-tetra-*O*-benzyl-β-D-glucopyranose (13a) was obtained as a colorless syrup (yield = 124 mg, 82%). ^{1}H NMR (500 MHz, $CDCl_3$) δ 7.47 (d, *J* = 8.0 Hz, 2H), 7.32 (d, *J* = 7.0 Hz, 2H), 7.27-7.19 (m, 15H), 7.15-7.10 (m, 5H), 4.95-4.82 (m, 4H), 4.73 (d, *J* = 11.0 Hz, 1H), 4.50 (d, *J* = 11.0 Hz, 1H), 4.23 (d, *J* = 11.5 Hz, 1H), 4.13 (d, *J* = 12.0 Hz, 1H), 4.02 (t, *J*= 9.5 Hz, 1H), 3.87 (d, *J* = 10.0 Hz, 1H), 3.72 (t, *J* = 9.0 Hz, 1H), 3.51-3.44 (m, 3H), 3.28-3.24 (m, 1H), 2.21 (s, 3H). ^{13}C NMR (125 MHz, $CDCl_3$) δ 141.4, 138.2, 138.1, 137.6, 137.5, 136.1, 129.5, 128.5, 128.4, 128.4, 128.3, 128.2, 128.0, 128.0, 127.8, 127.7, 127.6, 127.5, 125.3, 93.4, 86.5, 80.6, 77.5, 76.8, 75.7, 75.6, 75.0, 73.5, 68.9, 21.3. HRMS: Calc. for $C_{41}H_{42}O_6SNa$ $[M+Na]^+$: 685.2600, Obser. 685.2613.

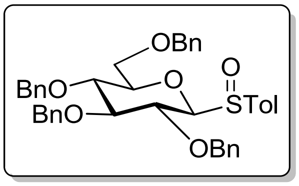

4.14 1-Deoxy-1-[(*R*/*S*)-(4-methylphenyl)sulfinyl]-2,3-di-O-allyl-4,6-di-*O*-acetyl-β-D-glucopyranose (14a) was obtained as a white solid (yield = 103 mg, 89%). ^{1}H NMR (500 MHz, $CDCl_3$) δ 7.56-7.52 (m, 2H), 7.31-7.29 (m, 2H), 5.85-5.79 (m, 2H), 5.39-5.22 (m, 2H), 5.19-5.13 (m, 2H), 4.94-4.83 (m, 1H), 4.48-4.40 (m, 1H), 4.35-4.26 (m, 2H), 4.23-4.12 (m, 2H), 4.09-4.01 (m, 1H), 3.93-3.90 (m, 1H), 3.79-3.35 (m, 3H), 2.41-2.40 (m, 3H), 2.05-1.86 (m, 6H). ^{13}C NMR (125 MHz, $CDCl_3$) δ 170.5, 170.3, 169.3, 169.2,

141.8, 141.4, 136.5, 136.1, 134.3, 134.1, 134.0, 133.9, 129.4, 129.4, 125.7, 125.5, 118.0, 117.2, 117.1, 94.8, 92.9, 83.2, 83.2, 76.6, 76.2, 76.1, 75.9, 74.4, 74.0, 74.0, 73.3, 69.7, 69.0, 62.4, 61.9, 21.4, 21.3, 20.8, 20.7, 20.6, 20.4.HRMS: Calc. for $C_{23}H_{30}O_8SNa$ $[M+Na]^+$: 489.1559, Obser. 489.1561.

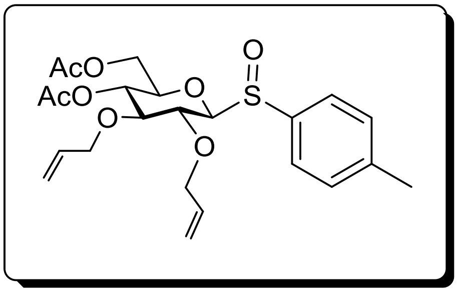

4.15 1,2-Dideoxy-1-[(*R*/*S*)-(phenyl)sulfinyl]-2-*N*-phthalimido-3,4,6-tri-*O*-acetyl-β-D-glucopyranose (15a) was obtained as a colorless syrup (yield = 108 mg, 91%). ^{1}H NMR (500 MHz, $CDCl_3$) δ 7.88-7.47 (m, 7H), 7.22-7.12 (m, 2H), 5.79-5.73 (m, 1H), 5.45-5.40 (m, 1H), 5.17-5.06 (m, 1H), 4.92-4.66 (m, 1H), 4.28-4.12 (m, 2H), 3.93-3.80 (m, 1H), 2.08-1.99 (m, 6H), 1.84 (m, 3H). ^{13}C NMR (125 MHz, $CDCl_3$) δ 171.0, 170.4, 170.3, 170.1, 169.1, 139.2, 138.2, 134.4, 134.0, 131.5, 131.1, 130.5, 128.7, 128.7, 125.7, 124.4, 123.7, 123.3, 89.3, 86.3, 76.6, 76.3, 71.5, 71.4, 68.1, 67.93, 61.6, 61.65, 60.2, 49.6, 48.1, 20.65, 20.4, 20.2.HRMS: Calc. for $C_{26}H_{26}O_{10}S$ $[M+H]^+$: 544.1277, Obser. 544.1269.

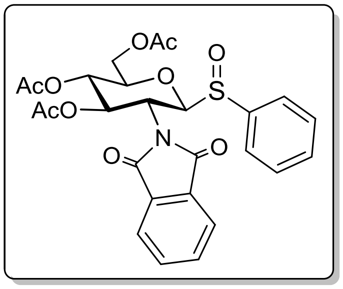

4.16 1-Deoxy-1-[(*R*/*S*)-(4-methylphenyl)sulfinyl]-2,3,4,6-tetra-*O*-acetyl-β-D-Galatopyranose (16a) was obtained as a white solid (yield = 94 mg, 80%). ^{1}H NMR

(500 MHz, $CDCl_3$) δ 7.55-7.48 (m, 2H), 7.26 (d, *J* = 8.0 Hz, 2H), 5.48-5.39 (m, 1H), 5.28-5.27 (m, 1H), 5.03-4.9 (m, 1H), 4.26-4.14 (m, 1H), 3.98-3.95 (m, 1H), 3.90-3.79 (m, 2H), 2.35 (s, 3H), 2.00 – 1.87 (m, 12H). ^{13}C NMR (125 MHz, $CDCl_3$) δ 170.2, 170.1, 169.9, 169.8, 169.6, 168.9, 142.1, 136.1, 135.6, 129.4, 129.3, 125.9, 125.8, 92.2, 90.2, 75.1, 74.8, 71.9, 71.5, 66.8, 66.7, 65.1, 64.5, 61.1, 60.9, 21.4, 20.7, 20.6, 20.5, 20.4, 20.3. HRMS: Calc. for $C_{21}H_{26}O_{10}SNa$ $[M+Na]^+$: 493.1144, Obser. 493.1153.

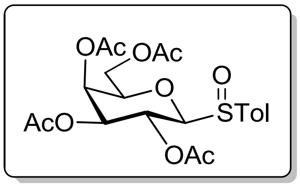

4.17 1-Deoxy-1-[(*R*/*S*)-(4-methylphenyl)sulfinyl]-2,3,4,6-tetra-*O*-benzoyl-β-D-galatopyranose (17a) was obtained as a colorless syrup (yield = 138 mg, 77%). ^{1}H NMR (500 MHz, $CDCl_3$) δ 7.90-7.85 (m, 3H), 7.79 (d, *J* = 7.0 Hz, 1H), 7.67-7.64 (m, 4H), 7.55-7.15 (m, 16H), 6.08-5.78 (m, 2H), 5.59-5.56 (m, 1H), 4.83 (d, *J* = 10.0 Hz, 1H), 4.56-4.50 (m, 1H), 4.35-4.18 (m, 2H), 2.29-2.23 (m, 3H). ^{13}C NMR (125 MHz, $CDCl_3$) δ 165.9, 165.5, 165.4, 165.0, 142.1, 135.8, 134.9, 133.6, 133.5, 133.5, 133.3, 133.2, 130.1, 129.9, 129.8, 129.8, 129.7, 129.7, 129.6, 129.5, 129.3, 129.2, 128.9, 128.7, 128.6, 128.6, 128.5, 128.4, 128.3, 128.3, 128.2, 126.4, 125.9, 92.8, 90.5, 77.2, 75.9, 75.4, 72.8, 72.6, 68.0, 67.7, 65.5, 65.2, 62.2, 61.6, 21.5, 21.5. HRMS: Calc. for $C_{41}H_{34}O_{10}SNa$ $[M+Na]^+$: 741.1770, Obser. 741.1754.

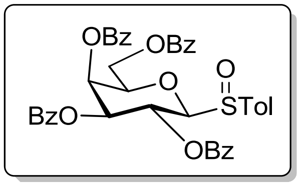

4.18 1-Deoxy-1-[(*R*/*S*)-(phenyl)sulfinyl]-2,3,4,6-tetra-*O*-acetyl-*α*-D-mannopyranose (18a) was obtained as a yellowish solid (yield = 96 mg, 85%). ^{1}H NMR (500 MHz, $CDCl_3$) δ 7.74-7.69 (m, 2H), 7.60-7.54 (m, 3H), 5.75-5.63 (m, 2H), 5.37-5.32 (m, 1H), 4.82-4.50 (m, 2H), 4.30-4.26 (m, 1H), 4.19-4.15 (m, 1H), 2.12-2.00 (m, 12H). ^{13}C NMR (125 MHz, $CDCl_3$) δ 170.3, 169.5, 169.4, 169.3, 139.7, 139.5, 131.8, 131.7, 129.4,

129.4, 124.9, 124.2, 94.5, 93.4, 74.5, 74.4, 69.3, 69.2, 65.8, 65.7, 65.5, 65.3, 62.4, 62.2, 20.6, 20.5, 20.5, 20.4. HRMS: Calc. for $C_{20}H_{24}O_{10}SNa$ $[M+Na]^+$: 479.0988, Obser. 479.0991.

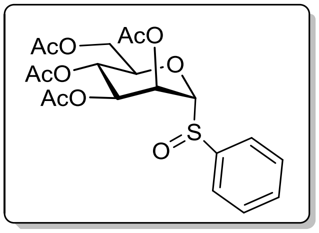

4.19 1-Deoxy-1-[(*R*/*S*)-(phenyl)sulfinyl]-2,3,4,6-tetra-*O*-benzoyl-α-D-mannopyranose (19a) was obtained as a colorless syrup (yield = 151 mg, 86%). ^{1}H NMR (500 MHz, $CDCl_3$) δ 8.11-7.98 (m, 5H), 7.91-7.80 (m, 5H), 7.59-7.26 (m, 15H), 6.36-6.19 (m, 2H), 6.16-6.06 (m, 1H), 5.33-5.12 (m, 1H), 4.90-4.77 (m, 2H), 4.60-4.52 (m, 1H). ^{13}C NMR (125 MHz, $CDCl_3$) δ 166.0, 165.4, 165.1, 164.7, 139.7, 133.5, 133.4, 133.3, 133.1, 133.0, 131.9, 131.8, 130.0, 129.8, 129.7, 129.7, 129.7, 129.6, 129.6, 129.5, 128.8, 128.7, 128.7, 128.6, 128.4, 128.4, 128.3, 128.2, 124.9, 124.3, 94.6, 93.6, 75.0, 74.8, 70.4, 70.3, 69.4, 67.0, 66.2, 66.0, 62.8, 62.7.HRMS: Calc. for $C_{40}H_{32}O_{10}SNa$ $[M+Na]^+$: 727.1614, Obser. 727.1607.

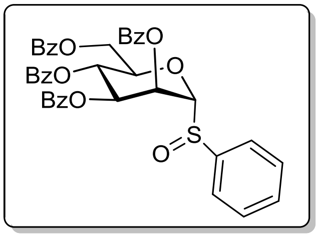

5.0 Analytical data for the glycosyl sulfones

5.1 Phenylsulfonyl 2,3,4,6-tetra-*O*-acetyl-1-deoxy-β-D-glucopyranoside (1b) was obtained as a white solid (yield = 109 mg, 93%).^{1}H NMR (500 MHz, $CDCl_3$) δ 7.85-7.84 (m, 2H), 7.65 (t, *J* = 7.5 Hz, 1H), 7.52 (t, *J* = 8.0 Hz, 2H), 5.16 (t, *J*= 4.0 Hz, 2H), 4.81 (t, *J* = 10.0 Hz, 1H), 4.44 (d, *J* = 9.5 Hz, 1H), 4.06 (d, *J* = 3.5 Hz, 2H), 3.66 (dt, *J* = 10.0, 3.5 Hz, 1H), 2.06 (s, 3H), 1.93-1.92 (m, 6H), 1.90 (s, 3H). ^{13}C NMR (125 MHz, $CDCl_3$) δ 170.1, 169.9, 169.3, 169.1, 134.6, 134.5, 130.5, 128.8, 88.7, 76.1, 73.2, 67.1, 66.9, 61.1, 20.6, 20.5, 20.4, 20.4.HRMS: Calc. for $C_{20}H_{24}O_{11}SNa$ $[M+Na]^+$: 495.0937, Obser. 495.0954.

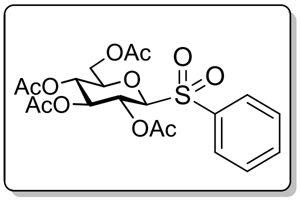

5.2 Phenylsulfonyl 2,3,4,6-tetra-*O*-benzoyl-1-deoxy-β-D-glucopyranoside (2b) was obtained as a white solid (yield = 163 mg, 91%). ^{1}H NMR (500 MHz, $CDCl_3$) δ 7.89 (dd, *J* = 14.5, 7.0 Hz, 4H), 7.81 (dd, *J* = 13.5, 7.0 Hz, 4H), 7.71 (d, *J* = 7.0 Hz, 2H), 7.51-7.40 (m, 5H), 7.36-7.18 (m, 10H), 5.84 (t, *J* = 9.5 Hz, 1H), 5.70 (t, *J* = 9.5 Hz, 1H), 5.42 (t, *J* = 9.5 Hz, 1H), 4.81 (d, *J* = 10.0 Hz, 1H), 4.57 (dd, *J* = 12.5, 3.0 Hz, 1H), 4.30 (dd, *J* = 12.0, 4.5 Hz, 1H), 4.13-4.09 (m, 1H).^{13}C NMR (125 MHz, $CDCl_3$) δ 165.7, 165.6, 165.0, 164.9, 134.6, 134.4, 133.6, 133.3, 133.2, 130.4, 130.1, 129.9, 129.8, 129.7, 129.2, 128.9, 128.8, 128.4, 128.4, 128.3, 128.3, 89.2, 73.5, 68.2, 67.6, 61.9, 60.3.HRMS: Calc. for $C_{40}H_{32}O_{11}SNa$ $[M+Na]^+$: 743.1563, Obser. 743.1583.

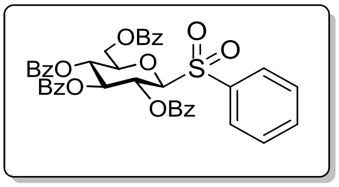

***5*.3 *p*-Methylphenylsulfonyl 2,3,4,6-tetra-*O*-acetyl-1-deoxy-β-D-glucopyranoside (3b)** was obtained as a white solid (yield = 114 mg, 94%). ^{1}H NMR (500 MHz, $CDCl_3$) δ 7.71 (d, *J*

= 7.5 Hz, 2H), 7.30 (d, *J* = 7.5 Hz, 2H), 5.17-5.09 (m, 2H), 4.80 (t, *J* = 9.5 Hz, 1H), 4.41 (d, *J* = 9.5 Hz, 1H), 4.06 (s, 2H), 3.65 (d, *J* = 10.0 Hz, 1H), 2.40 (s, 3H), 2.05 (s, 3H), 1.92-1.91(m, 9H). ^{13}C NMR (125 MHz, $CDCl_3$): δ 170.2, 169.9, 169.3, 169.2, 145.8, 131.3, 130.5, 129.5, 88.7, 76.0, 73.3, 67.2, 67.0, 61.1, 21.7, 20.6, 20.5, 20.4, 20.4. HRMS: Calc. for $C_{21}H_{26}O_{11}SNa$ $[M+Na]^+$: 509.1094, Obser. 509.1125.

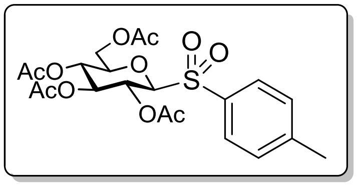

5.4 ***p*****-Methylphenylsulfonyl 2,3,4,6-tetra-*O*-benzoyl-1-deoxy-β-D-glucopyranoside (4b)** was obtained as a white solid (yield = 168 mg, 92%). ^{1}H NMR (500 MHz, $CDCl_3$) δ 7.91 (d, *J* = 8.0 Hz, 2H), 7.86 (d, *J* = 7.5 Hz, 2H), 7.79 (d, *J* = 8.0 Hz, 2H), 7.75-7.70 (m, 4H), 7.52(t, *J* = 7.5 Hz, 1H), 7.46-7.18 (m, 11H), 7.12 (d, *J* = 8.0 Hz, 2H), 5.82 (t, *J* = 9.5 Hz, 1H), 5.65 (t, *J* = 9.5 Hz, 1H), 5.43 (t, *J* = 9.5 Hz, 1H), 4.78 (d, *J* = 10.0 Hz, 1H), 4.62 (dd, *J* = 12.5, 2.5 Hz, 1H), 4.30 (dd, *J* = 12.5, 4.5 Hz, 1H), 4.13-4.10 (m, 1H), 2.23 (s, 3H).^{13}C NMR (125 MHz, $CDCl_3$) δ 165.7, 165.6, 165.0, 164.9, 145.7, 133.6, 133.3, 133.3, 133.2, 131.4, 130.5, 129.9, 129.8, 129.7, 129.5, 129.39, 128.9, 128.4, 128.4, 128.40, 128.3, 128.2, 89.2, 76.6, 73.6, 68.2, 67.7, 61.9, 21.6. HRMS: Calc. for $C_{41}H_{34}O_{11}SNa$ $[M+Na]^+$: 757.1720, Obser. 757.1762.

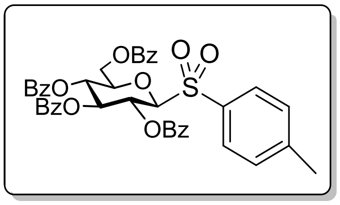

5.5 Benzylsulfonyl 2,3,4,6-tetra-*O*-acetyl-1-deoxy-β-D-glucopyranoside (5b) was obtained as a white solid (yield = 114 mg, 94%). ^{1}H NMR (500 MHz, $CDCl_3$) δ 7.39-7.34 (m, 5H), 5.44 (t, *J* = 9.5 Hz, 1H), 5.15 (t, *J* = 9.5 Hz, 1H), 5.03 (t, *J* = 10.0 Hz, 1H), 4.44 (d, *J* = 14.0 Hz, 1H), 4.27 (dd, *J* = 12.5, 1.5 Hz, 1H), 4.21-4.15 (m, 3H), 3.69-3.65 (m, 1H), 2.09 (s, 3H), 1.97 (s, 3H), 1.94-1.93 (m, 6H).^{13}C NMR (125 MHz, $CDCl_3$) δ 170.3,

170.0, 169.1, 169.0, 130.9, 129.2, 129.0, 126.5, 85.0, 76.5, 73.0, 67.4, 65.8, 61.7, 56.1, 20.7, 20.5, 20.4. HRMS: Calc. for $C_{21}H_{26}O_{11}SNa$ $[M+Na]^+$: 509.1094, Obser. 509.1112.

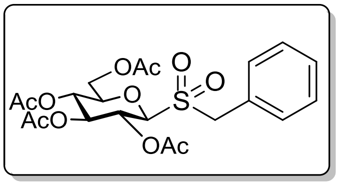

5.6 Benzylsulfonyl 2,3,4,6-tetra-*O*-benzoyl-1-deoxy-β-D-glucopyranoside (6b) was obtained as a white solid (yield = 163 mg, 89%). ^{1}H NMR (500 MHz, $CDCl_3$) δ 8.08 (d, *J*= 7.5 Hz, 2H), 7.91 (d, *J* = 7.5 Hz, 2H), 7.87 (d, *J* = 7.5 Hz, 2H), 7.81 (d, *J* = 8.0 Hz, 2H), 7.57 (t, *J* = 7.5 Hz, 1H), 7.51-7.26 (m, 16H), 6.08 (t, *J* = 9.5 Hz, 1H), 5.91 (t, *J* = 9.5 Hz, 1H), 5.69 (t, *J* = 9.5 Hz, 1H), 4.81 (dd, *J* = 13.0, 3.0 Hz, 1H), 4.64-4.54 (m, 3H), 4.29 (d, *J* = 13.5 Hz, 1H), 4.24-4.21 (m, 1H).^{13}C NMR (125 MHz, $CDCl_3$) δ 166.0, 165.6, 165.0, 164.6, 133.6, 133.4, 133.3, 133.2, 130.9, 129.9, 129.8, 129.7, 129.7, 129.2, 129.1, 128.9, 128.7, 128.5, 128.4, 128.3, 128.3, 128.3, 128.2, 126.5, 85.4, 77.0, 73.2, 68.6, 66.5, 62.8, 56.3.HRMS: Calc. for $C_{41}H_{35}O_{11}S$ $[M+H]^+$: 735.1900, Obser. 735.1910.

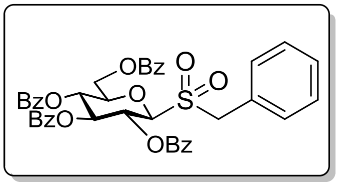

5.7 Ethylsulfonyl 2,3,4,6-tetra-*O*-acetyl-1-deoxy-β-D-glucopyranoside (7b) was obtained as a white solid (yield = 99 mg, 94%). ^{1}H NMR (500 MHz, $CDCl_3$) δ 5.49 (t, *J* = 9.5 Hz, 1H), 5.32 (t, *J* = 9.5 Hz, 1H), 5.12 (t, *J* = 10.0 Hz, 1H), 4.48 (d, *J* = 10.0 Hz, 1H), 4.26-4.22 (m, 2H), 3.86-3.83 (m, 1H), 3.18-3.11 (m, 2H), 2.08-2.02 (m, 12H), 1.40 (t, *J* = 7.5 Hz, 3H).^{13}C NMR (125 MHz, $CDCl_3$) δ 170.3, 169.9, 169.2, 169.1, 87.3, 76.6, 73.0, 67.3, 66.3, 61.3, 43.8, 20.5, 20.5, 20.4, 20.4, 5.3.HRMS: Calc. for $C_{16}H_{24}O_{11}SNa$ $[M+Na]^+$: 447.0937, Obser. 447.0940.

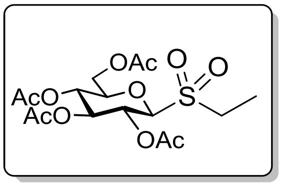

5.8 Ethylsulfonyl 2,3,4,6-tetra-*O*-benzoyl-1-deoxy-β-D-glucopyranoside (8b) was obtained as a white solid (yield = 156 mg, 93%). ^{1}H NMR (500 MHz, $CDCl_3$) δ 8.02 (dd, *J* = 8.0, 1.0 Hz, 2H), 7.94-7.90 (m, 4H), 7.83 (dd, *J* = 8.0, 1.0 Hz, 2H), 7.57 (t, *J* = 7.5 Hz, 1H), 7.44-7.29 (m, 11H), 6.06-5.99 (m, 2H), 5.70 (t, *J* = 9.5 Hz, 1H), 4.85 (d, *J* = 9.5 Hz, 1H), 4.69 (dd, *J* = 12.5, 2.5 Hz, 1H), 4.53 (dd, *J* = 12.5, 6.0 Hz, 1H), 4.31-4.28 (m, 1H), 3.26-3.14 (m, 2H), 1.36 (t, *J* = 7.5 Hz, 3H).^{13}C NMR (125 MHz, $CDCl_3$) δ 165.8, 165.6, 165.0, 164.8, 133.6, 133.4, 133.3, 133.3, 129.9, 129.8, 129.7, 129.6, 129.2, 128.8, 128.4, 128.4, 128.4, 128.3, 128.3, 87.6, 73.3, 68.6, 67.1, 62.5, 44.4, 5.68.HRMS: Calc. for $C_{36}H_{32}O_{11}SNa$ $[M+Na]^+$: 695.1563, Obser. 695.1571.

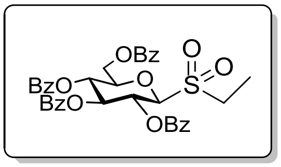

5.9 n-Hexylsulfonyl 2,3,4,6-tetra-*O*-acetyl-1-deoxy-β-D-glucopyranoside (9b) was obtained as a white solid (yield = 111 mg, 93%). ^{1}H NMR (500 MHz, $CDCl_3$) δ 5.41 (t, *J* = 9.5 Hz, 1H), 5.24 (t, *J* = 9.0 Hz, 1H), 5.05 (t, *J* = 9.5 Hz, 1H), 4.36 (d, *J* = 10.0 Hz, 1H), 4.21-4.12 (m, 2H), 3.77-3.75 (m, 1H), 3.03 (td, *J* = 10.0, 6.5 Hz, 2H), 2.02-1.96 (m, 12H), 1.79-1.71 (m, 2H), 1.37 (t, *J* = 6.5, 2H), 1.26-1.25 (m, 4H), 0.83 (t, *J* = 6.0 Hz, 3H).^{13}C NMR (125 MHz, $CDCl_3$) δ 170.3, 170.0, 169.3, 169.1, 87.6, 73.0, 67.3, 66.4, 61.4, 49.1, 31.1, 28.1, 22.2, 20.6, 20.6, 20.5, 20.4, 13.8. HRMS: Calc. for $C_{20}H_{32}O_{11}SNa$ $[M+Na]^+$: 503.1563, Obser. 503.1591.

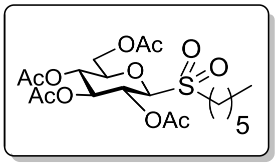

5.10 n-Hexylsulfonyl 2,3,4,6-tetra-*O*-benzoyl-1-deoxy-β-D-glucopyranoside (10b) was obtained as a white solid (yield = 165 mg, 91%). ^{1}H NMR (500 MHz, $CDCl_3$) δ 8.02 (d, *J*

= 7.5 Hz, 2H), 7.92 (dd, J = 12.5, 7.5 Hz, 4H), 7.83 (d, J = 7.0 Hz, 2H), 7.57-7.25 (m, 12H), 6.06-5.99 (m, 2H), 5.70 (t, J = 10.0 Hz, 1H), 4.82 (d, J = 9.0 Hz, 1H), 4.68 (dd, J = 12.0, 2.5 Hz, 1H), 4.54 (dd, J = 12.5, 5.5 Hz, 1H), 4.32-4.29 (m, 1H), 3.17-3.13 (m, 2H), 1.85-1.75 (m, 2H), 1.33-1.18 (m, 6H), 0.83 (t, J = 7.0 Hz, 3H).^{13}C NMR (125 MHz, $CDCl_3$) δ 165.8, 165.6, 165.0, 164.8, 133.6, 133.4, 133.3, 133.3, 129.9, 129.8, 129.7, 129.6, 129.2, 128.8, 128.4, 128.4, 128.3, 128.3, 128.3, 87.9, 77.1, 73.3, 68.6, 67.1, 62.5, 49.7, 31.0, 28.1, 22.2, 20.7, 13.8.HRMS: Calc. for $C_{40}H_{40}O_{11}SNa$ $[M+Na]^+$: 751.2189, Obser. 751.2194.

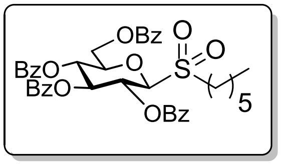

5.11 Cyclohexylsulfonyl 2,3,4,6-tetra-*O*-acetyl-1-deoxy-β-D-glucopyranoside (11b) was obtained as a white solid (yield = 110 mg, 92%). ^{1}H NMR (500 MHz, $CDCl_3$) δ 5.50 (t, J = 9.5 Hz, 1H), 5.26 (t, J = 9.5 Hz, 1H), 5.02 (t, J = 10.0 Hz, 1H), 4.54 (d, J = 10.0 Hz, 1H), 4.15-4.11 (m, 2H), 3.73-3.71 (m, 1H), 3.21-3.16 (m, 1H), 2.11 (d, J = 12.5 Hz, 1H), 2.01 (s, 3H), 1.98-1.96 (m, 9H), 1.90-1.82 (m, 2H), 1.66 (d, J = 12.0 Hz, 1H), 1.58-1.48 (m, 3H), 1.27-1.17 (m, 3H).^{13}C NMR (125 MHz, $CDCl_3$) δ 170.2, 170.1, 169.1, 169.0, 85.6, 76.6, 73.1, 67.5, 66.3, 61.7, 58.9, 25.9, 25.0, 24.9, 24.9, 22.7, 20.6, 20.5, 20.5, 20.4. HRMS: Calc. for $C_{20}H_{30}O_{11}SNa$ $[M+Na]^+$: 501.1407, Obser. 501.1423.

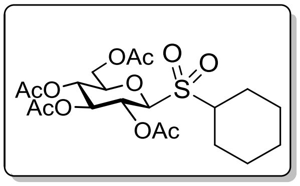

5.12 Cyclohexylsulfonyl 2,3,4,6-tetra-*O*-benzoyl-1-deoxy-β-D-glucopyranoside (12b) was obtained as a white solid (yield = 163 mg, 90%). ^{1}H NMR (500 MHz, $CDCl_3$) δ 8.03 (d, J = 7.0 Hz, 2H), 7.93 (dd, J = 8.5, 7.5 Hz, 4H), 7.84 (d, J = 7.5 Hz, 2H), 7.59-7.28 (m, 12H), 6.12 (t, J = 9.5 Hz, 1H), 6.02 (t, J = 9.5 Hz, 1H), 5.64 (t, J = 10.0 Hz, 1H), 4.97 (d, J = 9.5 Hz, 1H), 4.65 (dd, J = 12.5, 2.5 Hz, 1H), 4.53 (dd, J = 12.5, 7.0 Hz, 1H), 4.32-4.28 (m, 1H), 3.34-3.29 (m, 1H), 2.19 (d, J = 13.0 Hz, 1H), 2.01 (d, J = 12.5 Hz, 1H), 1.86 (d, J = 13.5 Hz, 1H), 1.70 (d, J = 13.0 Hz, 1H), 1.62-1.51 (m, 3H), 1.25-0.99 (m,

3H).^{13}C NMR (125 MHz, $CDCl_3$) δ 165.8, 165.7, 165.0, 164.7, 133.6, 133.4, 133.3, 133.2, 129.9, 129.8, 129.8, 129.6, 129.2, 128.9, 128.4, 128.4, 128.3, 128.3, 128.2, 85.8, 73.4, 68.7, 66.9, 62.9, 58.8, 25.9, 24.9, 24.8, 24.5, 22.6.HRMS: Calc. for $C_{40}H_{39}O_{11}S$ $[M+H]^+$: 727.2213, Obser. 727.2216.

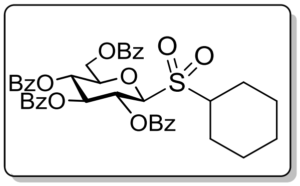

5.13 4-Methylphenylsulfonyl 2,3,4,6-tetra-*O*-benzyl-1-deoxy-β-D-glucopyranoside (13b)was obtained as a white solid (yield = 135 mg, 64%).^{1}H NMR (500 MHz, $CDCl_3$) δ 7.84-7.81 (m, 2H), 7.43 (m, 2H), 7.32-7.18 (m, 20H), 5.12-5.09 (m, 1H), 4.95-4.92 (m, 1H), 4.88-4.77 (m, 3H), 4.59-4.55 (m, 1H), 4.41-4.30 (m, 3H), 4.09-4.04 (m, 1H), 3.77-3.74 (m, 1H), 3.62-3.57 (m, 3H), 3.38-3.36 (m, 1H), 2.33 (s, 3H). ^{13}C NMR (125 MHz, $CDCl_3$) δ 144.9, 138.1, 138.0, 137.6, 137.5, 134.0, 129.6, 129.3, 128.6, 128.4, 128.4, 128.3, 128.3, 127.9, 127.8, 127.7, 127.6, 127.5, 91.0, 86.2, 79.6, 77.6, 76.9, 75.8, 75.3, 75.0, 73.4, 68.6, 21.5.HRMS: Calc. for $C_{41}H_{42}O_7SNa$ $[M+Na]^+$: 701.2549, Obser. 701.2543.

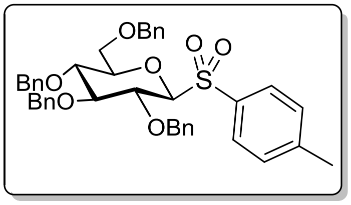

5.14 ***p*****-Methylphenylsulfonyl 2,3-di-*O*-allyl-4,6-di-*O*-acetyl-1-deoxy-β-D-glucopyranoside (14b)** was obtained as a colorless oil (yield = 109 mg, 91%). ^{1}H NMR (500 MHz, $CDCl_3$) δ 7.79 (d, *J* = 8.0 Hz, 2H), 7.34-7.32 (m, 2H), 6.05-5.97 (m, 1H), 5.88-5.80 (m, 1H), 5.35-5.31 (m, 1H), 5.25-5.15 (m, 3H), 4.87 (t, *J* = 9.5 Hz, 1H), 4.50 (dd, *J* = 11.5, 6.0 Hz, 1H), 4.32-4.28 (m, 2H), 4.27-4.25 (m, 1H), 4.12 (dd, *J* = 12.5, 6.0 Hz, 1H), 4.05 (dd, *J* = 12.0, 6.0 Hz, 1H), 3.93 (dd, *J* = 12.5, 2.0 Hz, 1H), 3.88 (t, *J* = 11.0 Hz, 1H), 3.53 (t, *J* = 9.0 Hz, 1H), 3.46-3.42 (m, 1H), 2.44 (s, 3H), 2.05 (m, 3H), 1.89 (m, 3H). ^{13}C NMR (125 MHz, $CDCl_3$) δ 170.2, 169.3, 145.0, 134.2, 134.1, 133.7,

129.8, 129.3, 117.9, 117.2, 90.6, 82.8, 77.16, 76.2, 74.4, 74.4, 69.0, 62.0, 21.6, 20.7, 20.4.HRMS: Calc. for $C_{23}H_{31}O_9S$ [M+H]$^+$: 483.1689, Obser. 483.1681.

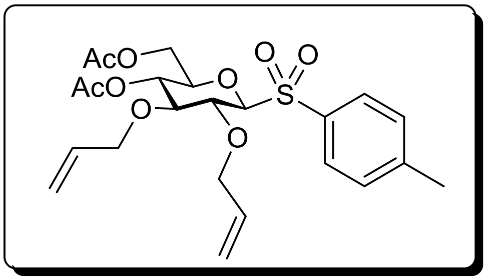

5.15 Phenylsulfonyl 3,4,6-tri-*O*-acetyl-2-*N*-phthalimido-1-deoxy-β-D-glucopyranoside (15b)was obtained as a white solid (yield = 114 mg, 82%).^{1}H NMR (500 MHz, $CDCl_3$) δ 7.90-7.88 (m, 4H), 7.76-7.75 (m, 2H), 7.71-7.68 (m, 1H), 7.57 (t, *J* = 7.5 Hz, 2H), 5.81 (t, *J* = 9.5 Hz, 1H), 5.52 (d, *J* = 10.5 Hz, 1H), 5.03 (t, *J* = 9.5 Hz, 1H), 4.62 (t, *J* = 10.0 Hz, 1H), 4.22-4.09 (m, 2H), 3.90-3.86 (m, 1H), 2.00-1.99 (m, 6H), 1.86 (s, 3H). ^{13}C NMR (125 MHz, $CDCl_3$) δ 170.2, 169.9, 169.2, 168.0, 166.6, 134.6, 134.5, 134.4, 134.3, 130.3, 128.8, 123.8, 85.3, 76.1, 70.9, 67.8, 61.2, 49.3, 20.5, 20.4, 20.2.HRMS: Calc. for $C_{26}H_{26}O_{11}S$ [M+H]$^+$: 560.1227, Obser. 560.1229.

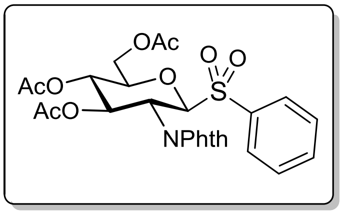

5.16 *p*-Methylphenylsulfonyl 2,3,4,6-tetra-*O*-acetyl-1-deoxy-β-D-galactopyranoside (16b) was obtained as a white solid (yield = 108 mg, 89%). ^{1}H NMR (500 MHz, $CDCl_3$) δ 7.76 (d, *J* = 8.5 Hz, 2H), 7.31 (d, *J* = 8.0 Hz, 2H), 5.36 (t, *J* = 10.0 Hz, 1H), 5.24 (d, *J* = 2.5 Hz, 1H), 4.96 (dd, *J* = 10.0, 3.0 Hz, 1H), 4.41 (d, *J* = 10.0 Hz, 1H), 4.03-3.99 (m, 1H), 3.89-3.85 (m, 2H), 2.39 (s, 3H), 2.06 (s, 3H), 1.91-1.89 (m, 6H), 1.84 (s, 3H).^{13}C NMR (125 MHz, $CDCl_3$): δ 170.1, 169.9, 169.7, 169.4, 145.5, 131.7, 130.6, 129.3, 89.0, 74.5, 71.4, 66.4, 63.9, 60.8, 21.6, 20.8, 20.5, 20.4, 20.2. HRMS: Calc. for $C_{21}H_{26}O_{11}SNa$[M+Na]$^+$: 509.1094, Obser. 509.1122.

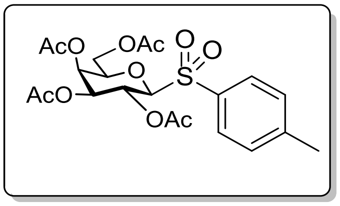

5.17 *p*-Methylphenylsulfonyl 2,3,4,6-tetra-*O*-benzoyl-1-deoxy-β-D-galactopyranoside (17b) was obtained as a colorless syrup (yield = 159 mg, 87%). ^{1}H NMR (500 MHz, $CDCl_3$) δ 7.92-7.85 (m, 6H), 7.68 (d, *J* = 7.0 Hz, 2H), 7.56 (d, *J* = 7.0 Hz, 2H), 7.53-7.27 (m, 12H), 7.17-7.14 (m, 2H), 5.95 (t, *J* = 10.0 Hz, 1H), 5.88 (d, *J* = 3.0 Hz, 1H), 5.51 (dd, *J* = 9.5, 3.0 Hz, 1H), 4.84 (d, *J* = 9.5 Hz, 1H), 4.50 (dd, *J* = 11.5, 6.5 Hz, 1H), 4.35 (t, *J* = 6.0 Hz, 1H), 4.23 (dd, *J* = 11.5, 6.0 Hz, 1H), 2.38 (s, 3H).^{13}C NMR (125 MHz, $CDCl_3$) δ 165.8, 165.3, 165.2, 164.9, 145.4, 133.5, 133.4, 133.3, 133.3, 131.8, 130.8, 129.8, 129.7, 129.7, 129.5, 129.1, 129.0, 128.5, 128.4, 128.3, 128.3, 128.2, 89.4, 75.2, 72.5, 67.6, 64.8, 61.8, 21.8. HRMS: Calc. for $C_{41}H_{34}O_{11}SNa$ $[M+Na]^+$: 757.1720, Obser. 757.1744.

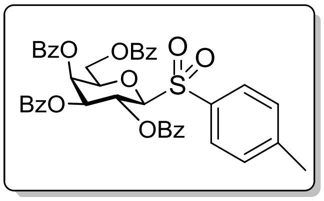

5.18 Phenylsulfonyl 2,3,4,6-tetra-*O*-acetyl-1-deoxy-α-D-mannopyranoside (18b) was obtained as a yellowish solid (yield = 108 mg, 92%). ^{1}H NMR (500 MHz, $CDCl_3$) δ 7.97-7.95 (m, 2H), 7.75-7.72 (m, 1H), 7.63-7.60 (m, 2H), 6.03 (d, *J* = 3.0 Hz, 1H), 5.73-5.69 (m, 1H), 5.29 (m, 1H), 4.82 (d, *J* = 1.5 Hz, 2H), 4.24-4.20 (m, 1H), 4.06 (dd, *J* = 12.5, 2.5 Hz, 1H), 2.12 (s, 3H), 2.08 (s, 3H), 2.05-2.03 (m, 6H).^{13}C NMR (125 MHz, $CDCl_3$) δ 170.2, 169.5, 169.2, 169.2, 136.1, 134.6, 129.3, 129.0, 90.1, 73.2, 68.7, 65.3, 65.1, 62.4, 20.5, 20.5, 20.4.HRMS: Calc. for $C_{20}H_{24}O_{11}SNa$ $[M+Na]^+$: 495.0937, Obser. 495.0941.

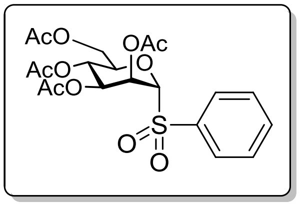

5.19 Phenylsulfonyl 2,3,4,6-tetra-*O*-benzoyl-1-deoxy-α-D-mannopyranoside (19b) was obtained as a colorless oil (yield = 160 mg, 89%). ^{1}H NMR (500 MHz, $CDCl_3$) δ 8.05-8.00 (m, 8H), 7.86 (d, *J* = 7.0 Hz, 2H), 7.58-7.57 (m, 3H), 7.54-7.51 (m, 1H), 7.47-7.37 (m, 9H), 7.27 (t, *J* = 8.0 Hz, 2H), 6.53-6.52 (m, 1H), 6.32 (dd, *J* = 1.0, 3.5 Hz, 1H), 6.16 (t, *J* = 10.0 Hz, 1H), 5.36-5.32 (m, 1H), 5.14 (d, *J* = 1.5 Hz, 1H), 4.62 (dd, *J* = 12.5, 2.5Hz, 1H), 4.55 (dd, *J* = 12.5, 5.0 Hz, 1H).^{13}C NMR (125 MHz, $CDCl_3$) δ 165.8, 165.4, 165.0, 164.8, 136.2, 134.6, 133.7, 133.6, 133.2, 133.1, 129.8, 129.8, 129.7, 129.6, 129.4, 129.0, 128.7, 128.7, 128.6, 128.5, 128.49, 128.4, 128.3, 90.5, 73.7, 69.7, 66.5, 65.8, 62.9.HRMS: Calc. for $C_{40}H_{32}O_{11}SNa$ $[M+Na]^+$: 743.1563, Obser. 743.1571.

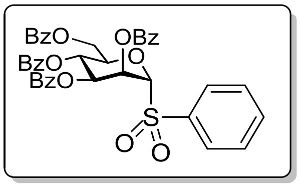

References

(1) Chen, B. L.; Wang, Y. J.; Guo, H.; Zeng, G. Y. *Eur J Med Chem* **2016**, *109*, 199.
(2) France, R. R.; Rees, N. V.; Wadhawan, J. D.; Fairbanks, A. J.; Compton, R. G. *Org Biomol Chem* **2004**, *2*, 2188.
(3) Cancogni, D.; Lay, L. *Synlett* **2014**, *25*, 2873.
(4) Liu, R. H.; Wei, A. *J Carbohyd Chem* **2012**, *31*, 384.
(5) Percec, V.; Leowanawat, P.; Sun, H. J.; Kulikov, O.; Nusbaum, C. D.; Tran, T. M.; Bertin, A.; Wilson, D. A.; Peterca, M.; Zhang, S. D.; Kamat, N. P.; Vargo, K.; Moock, D.; Johnston, E. D.; Hammer, D. A.; Pochan, D. J.; Chen, Y. C.; Chabre, Y. M.; Shiao, T. C.; Bergeron-Brlek, M.; Andre, S.; Roy, R.; Gabius, H. J.; Heiney, P. A. *J Am Chem Soc* **2013**, *135*, 9055.
(6) Weng, S. S.; Lin, Y. D.; Chen, C. T. *Organic letters* **2006**, *8*, 5633.
(7) Yuan, X. L.; Kou, Y. Z.; Yu, L.; Zhang, Z. X.; Xue, W. H. *Org Chem Front* **2015**, *2*, 1604.
(8) Xiao, X.; Zhao, Y. Q.; Shu, P. H.; Zhao, X.; Liu, Y.; Sun, J. C.; Zhang, Q.; Zeng, J.; Wan, Q. *J Am Chem Soc* **2016**, *138*, 13402.
(9) Sail, D.; Kovac, P. *Carbohyd Res* **2012**, *357*, 47.
(10) Saito, S.; Tsuchiya, T. *Chem Pharm Bull* **1985**, *32*, 503.
(11) Lahmann, M.; Oscarson, S. *Can J Chem* **2002**, *80*, 889.
(12) Yan, S. Q.; Ding, N.; Zhang, W.; Wang, P.; Li, Y. X.; Li, M. *J Carbohyd Chem* **2012**, *31*, 571.

(13) Mao, R. Z.; Xiong, D. C.; Guo, F.; Li, Q.; Duan, J. Y.; Ye, X. S. *Org Chem Front* **2016**, *3*, 737.
(14) Lee, D. J.; Kowalczyk, R.; Muir, V. J.; Rendle, P. M.; Brimble, M. A. *Carbohyd Res* **2007**, *342*, 2628.
(15) Skelton, B. W.; Stick, R. V.; Tilbrook, D. M. G.; White, A. H.; Williams, S. J. *Aust J Chem* **2000**, *53*, 389.
(16) Wang, Q.; Wei, X.; Liao, K. J.; Li, H.; Meng, X. B.; Li, Z. J. *Tetrahedron Lett* **2016**, *57*, 2277.
(17) Tsukamoto, H.; Kondo, Y. *Tetrahedron Lett* **2003**, *44*, 5247.
(18) Sanhueza, C. A.; Dorta, R. L.; Vazquez, J. T. *J Org Chem* **2011**, *76*, 7769.
(19) Agnihotri, G.; Misra, A. K. *Tetrahedron Lett* **2005**, *46*, 8113.
(20) Vincent, S. P.; Burkart, M. D.; Tsai, C. Y.; Zhang, Z. Y.; Wong, C. H. *J Org Chem* **1999**, *64*, 5264.
(21) Agnihotri, G.; Misra, A. K. *Carbohyd Res* **2006**, *341*, 275.
(22) Chen, M. Y.; Patkar, L. N.; Lin, C. C. *J Org Chem* **2004**, *69*, 2884.
(23) Morais, G. R.; Humphrey, A. J.; Falconer, R. A. *Tetrahedron* **2008**, *64*, 7426.
(24) Chittela, S.; Reddy, T. R.; Krishna, P. R.; Kashyap, S. *J Org Chem* **2015**, *80*, 7108.

^{1}H and ^{13}C spectra of sulfoxides

1-Deoxy-1-[(*R/S*)-(phenyl)sulfinyl]-2,3,4,6-tetra-*O*-acetyl-β-D-Glucopyranose (1a)

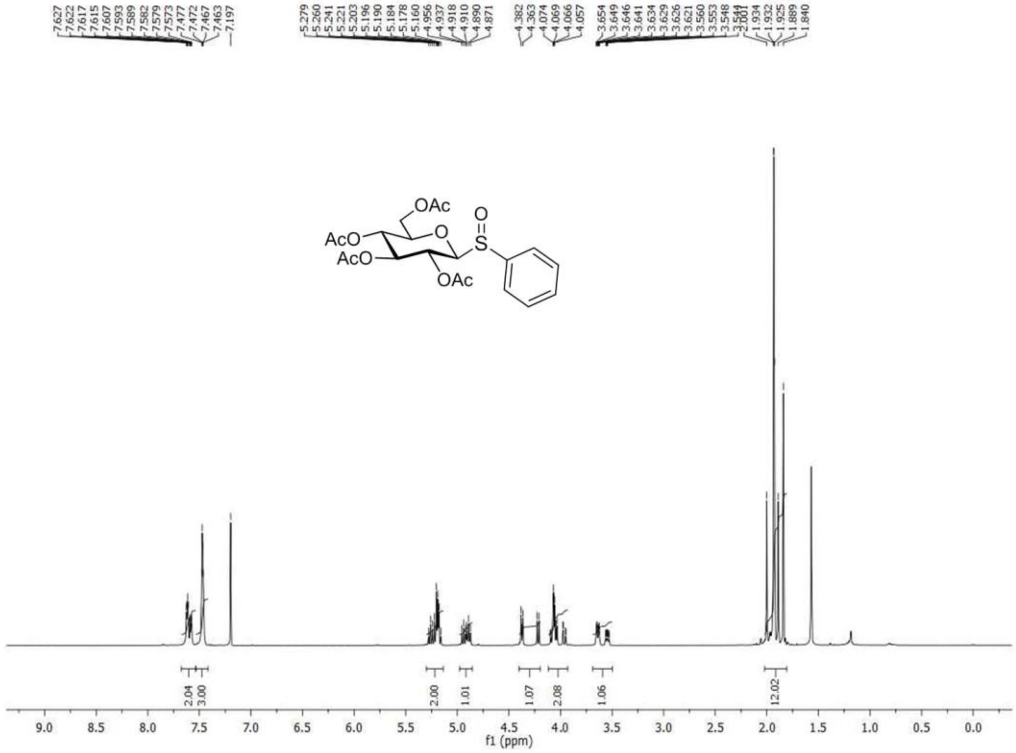

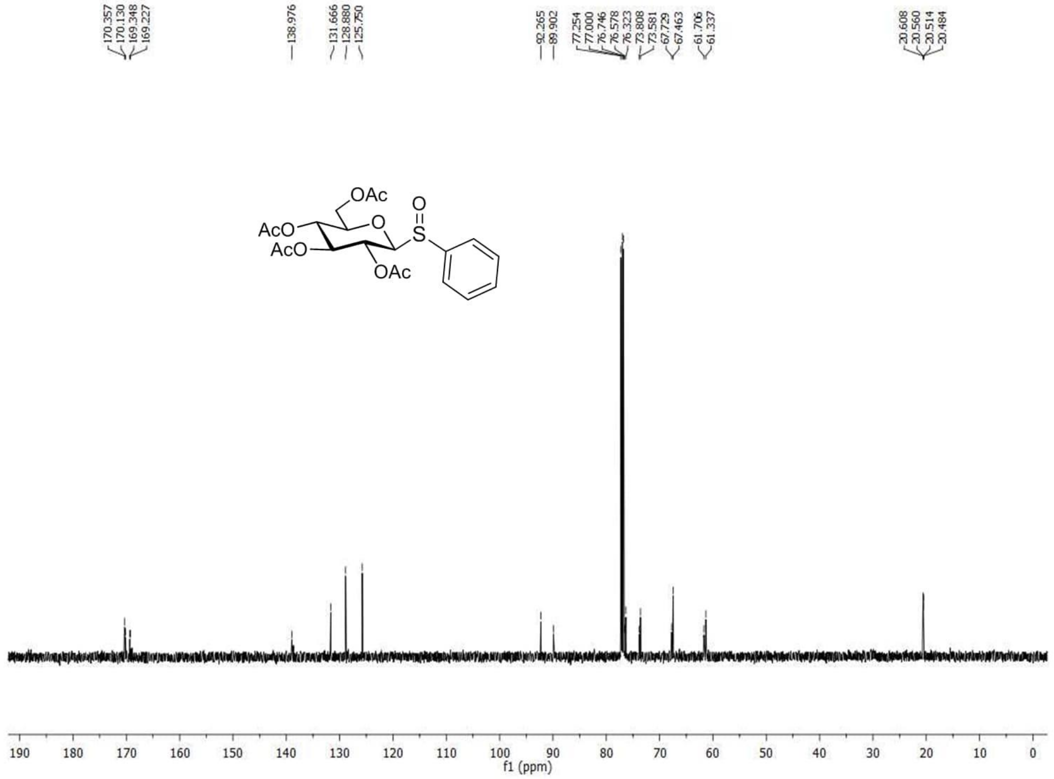

1-Deoxy-1-[(*R/S*)-(phenyl)sulfinyl]-2,3,4,6-tetra-*O*-benzoyl-β-D-Glucopyranose (2a)

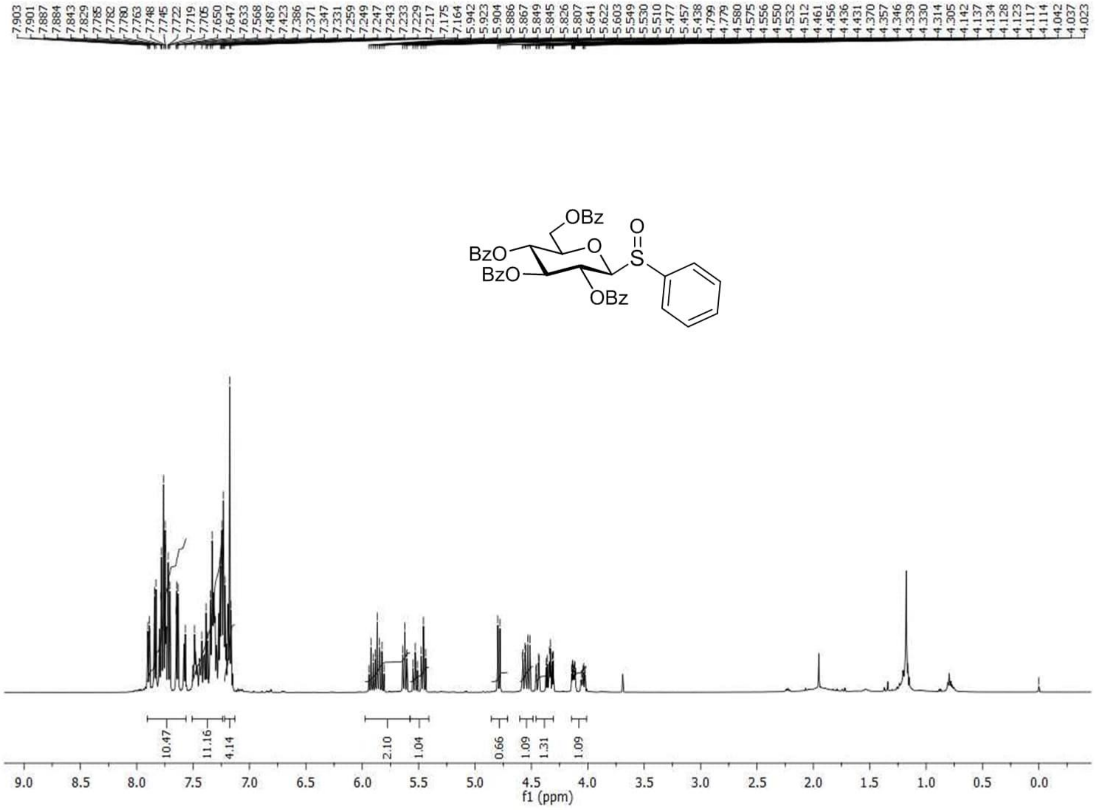

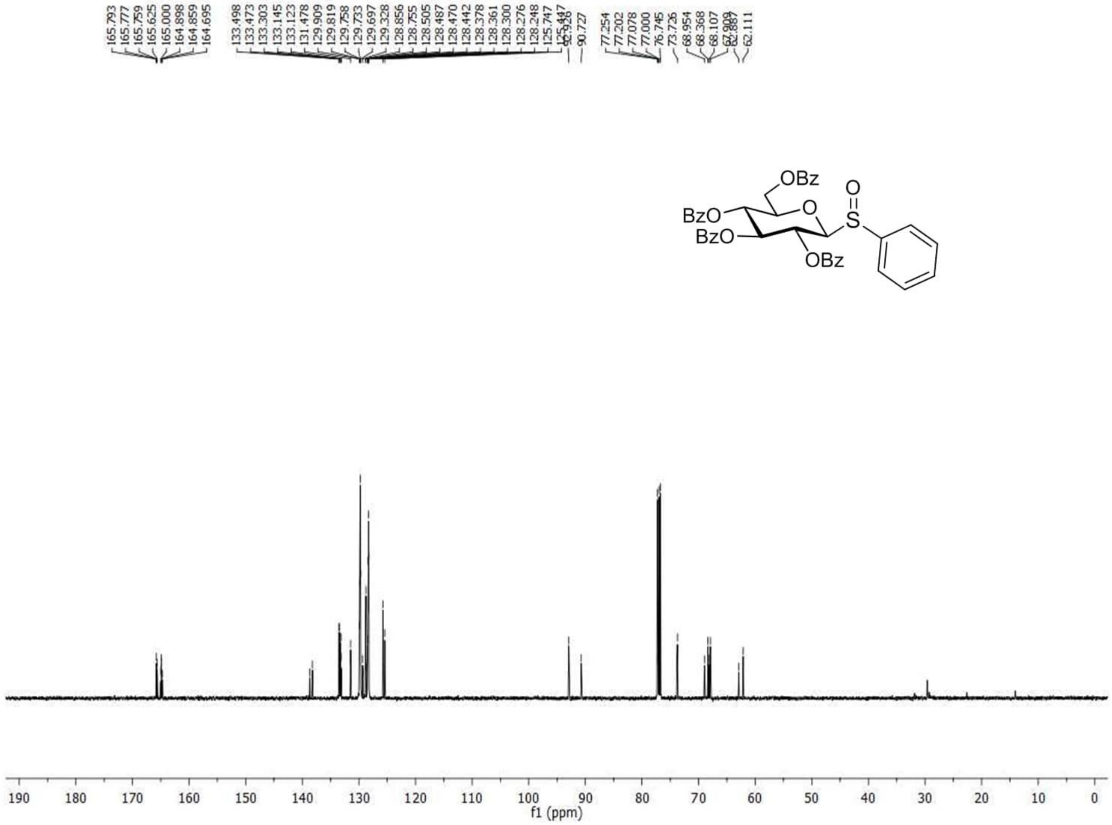

1-Deoxy-1-[(*R*/*S*)-(4-methylphenyl)sulfinyl]-2,3,4,6-tetra-*O*-acetyl-β-D-glucopyranose (3a)

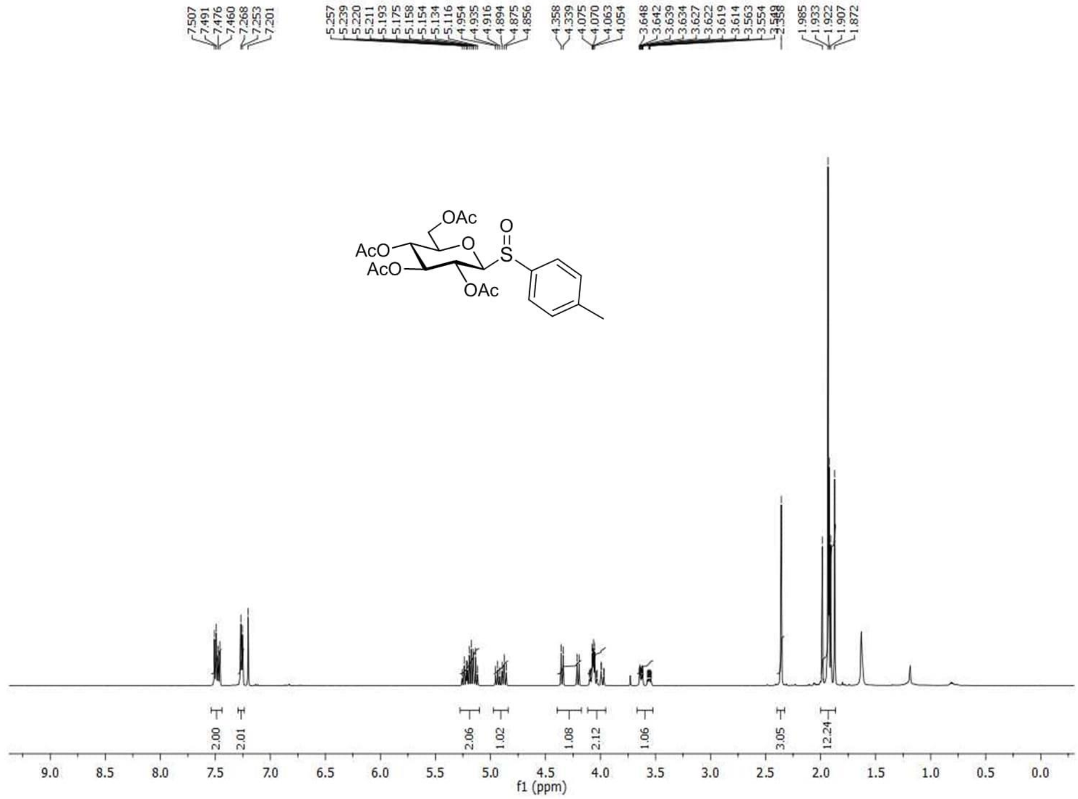

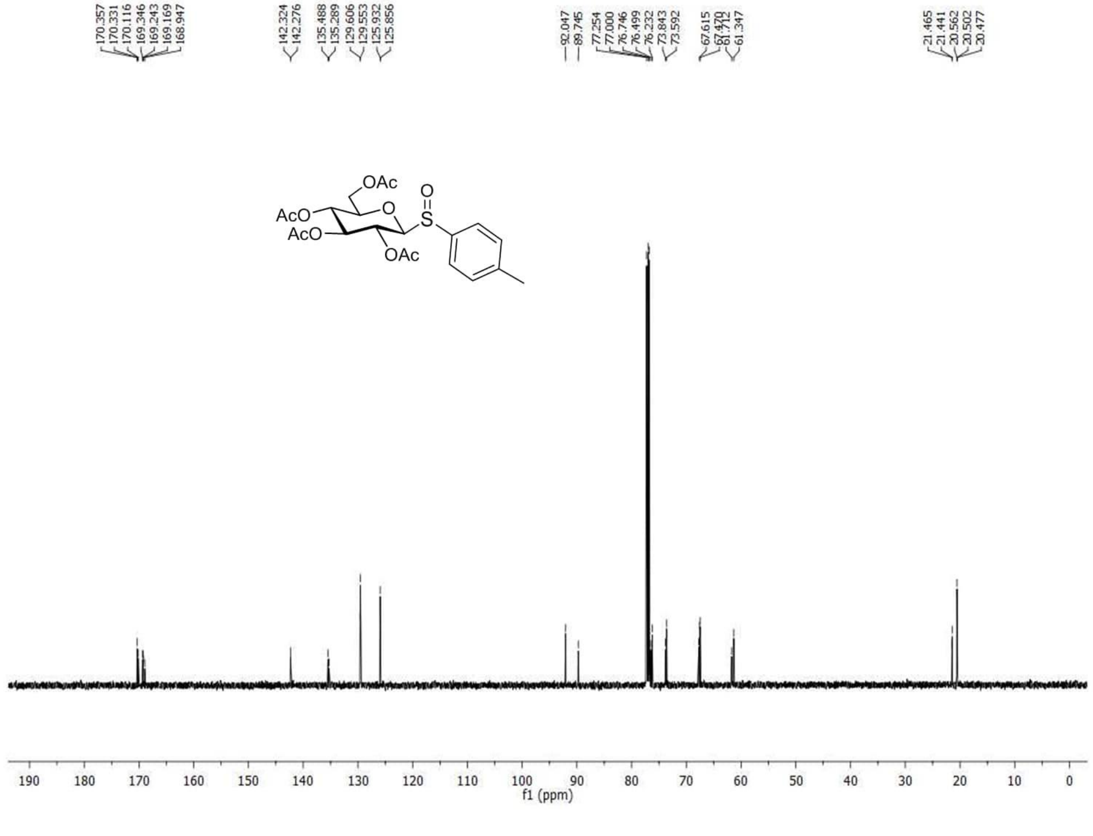

1-Deoxy-1-[(*R*/*S*)-(4-methylphenyl)sulfinyl]-2,3,4,6-tetra-*O*-benzoyl-β-D-glucopyranose (4a)

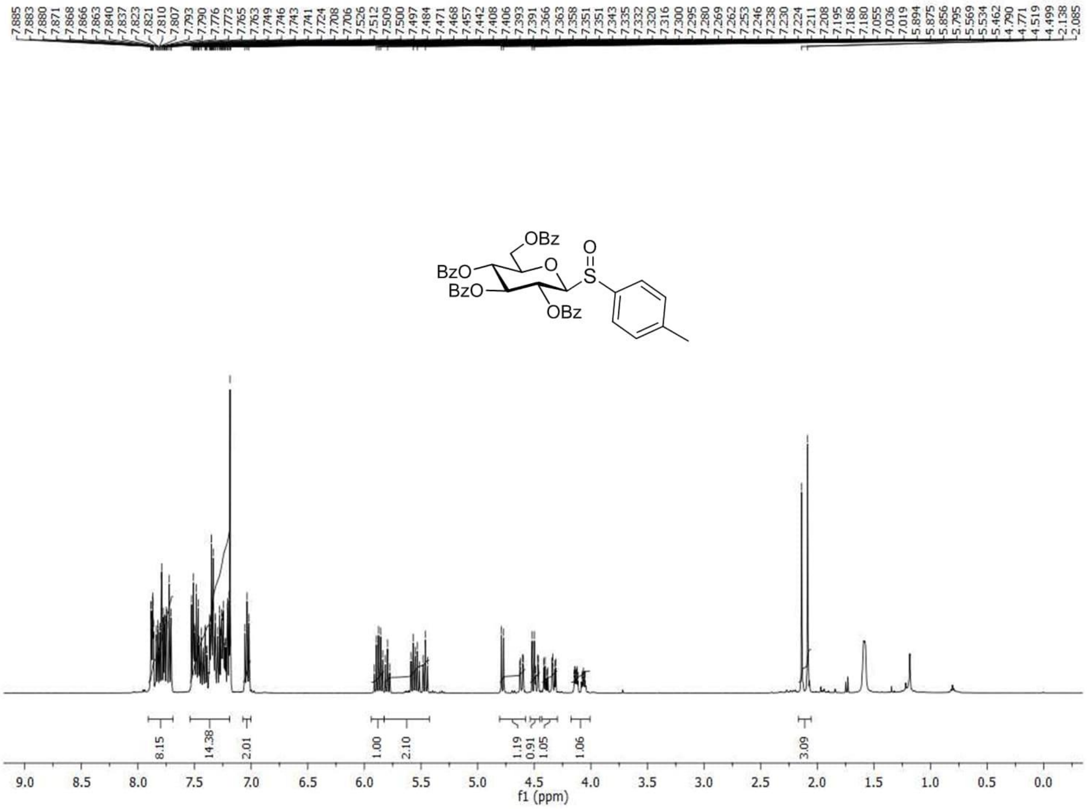

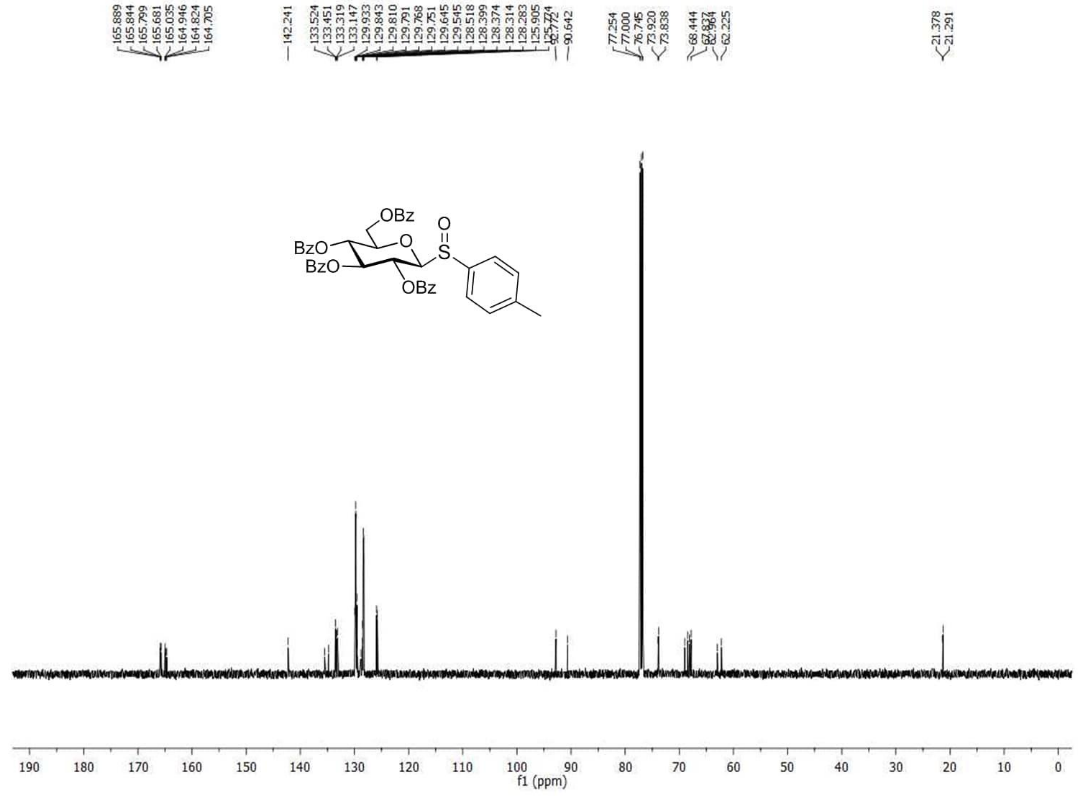

1-Deoxy-1-[(*R/S*)-(benzyl)sulfinyl]-2,3,4,6-tetra-*O*-acetyl-β-D-glucopyranose (5a)

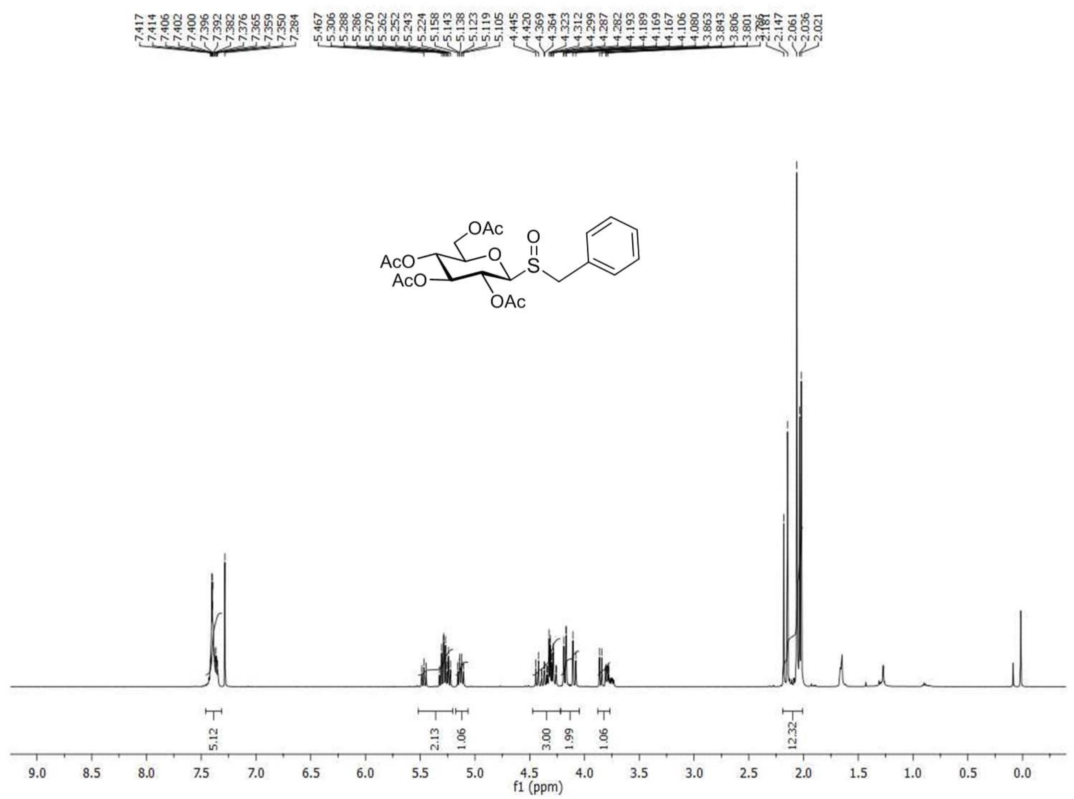

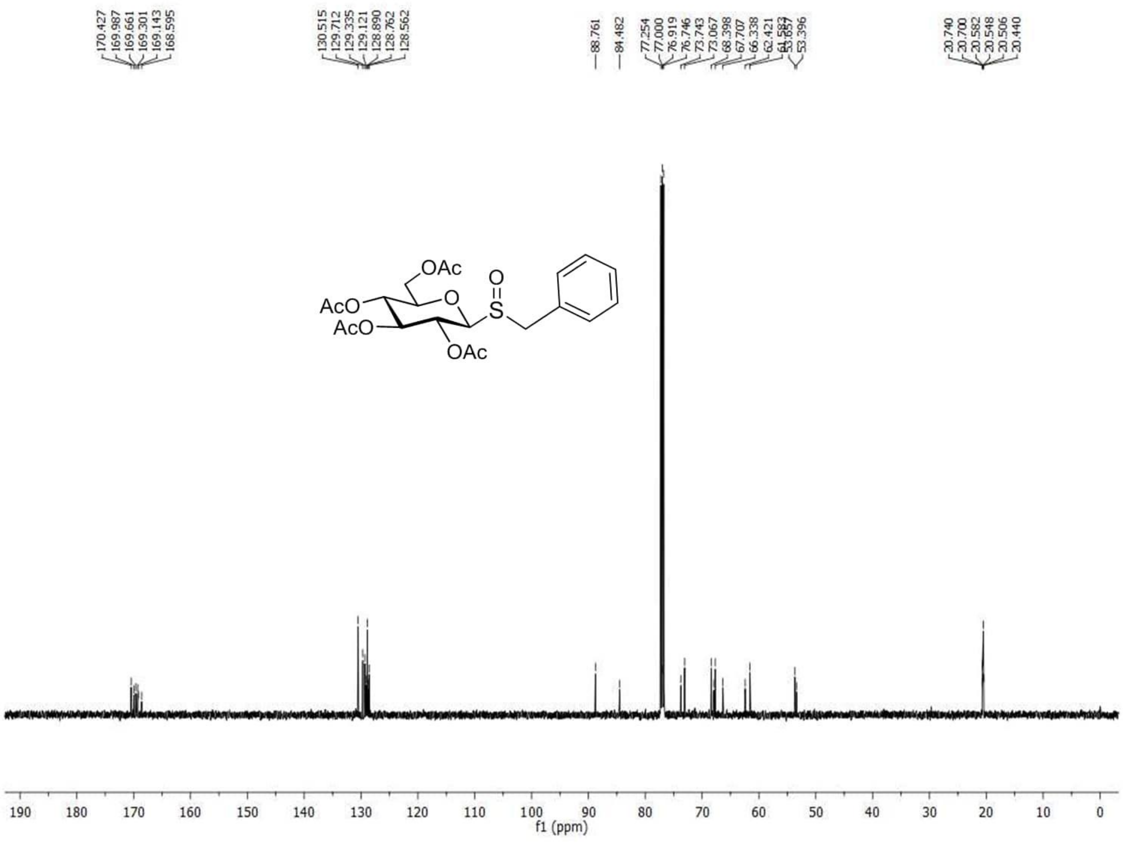

1-Deoxy-1-[(*R*/*S*)-(benzyl)sulfinyl]-2,3,4,6-tetra-*O*-benzoyl-β-D-glucopyranose (6a)

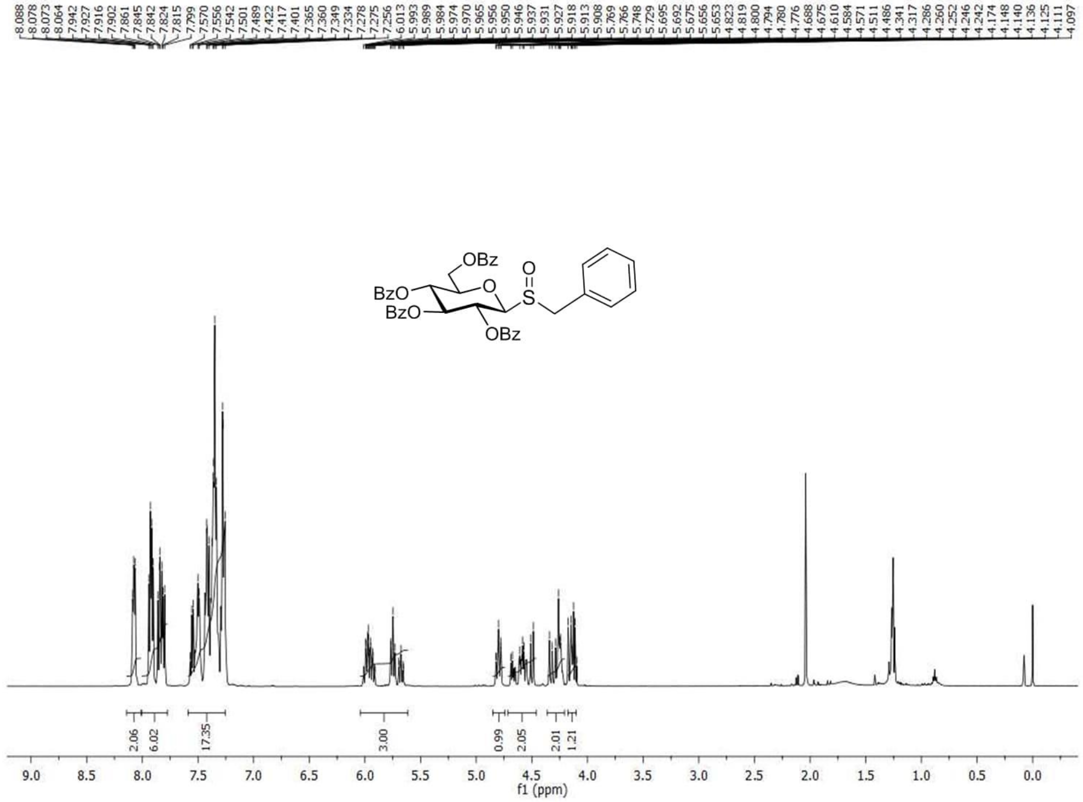

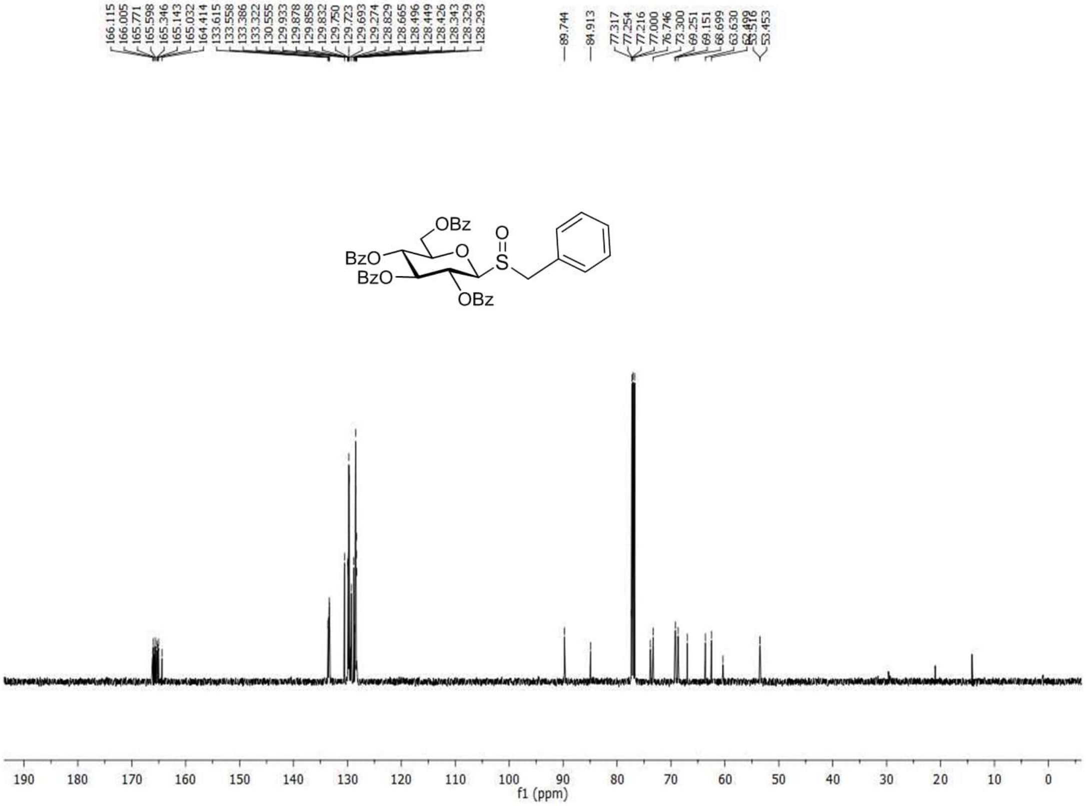

1-Deoxy-1-[(*R*/*S*)-ethylsulfinyl]-2,3,4,6-*O*-acetyl-β-D-glucopyranose (7a)

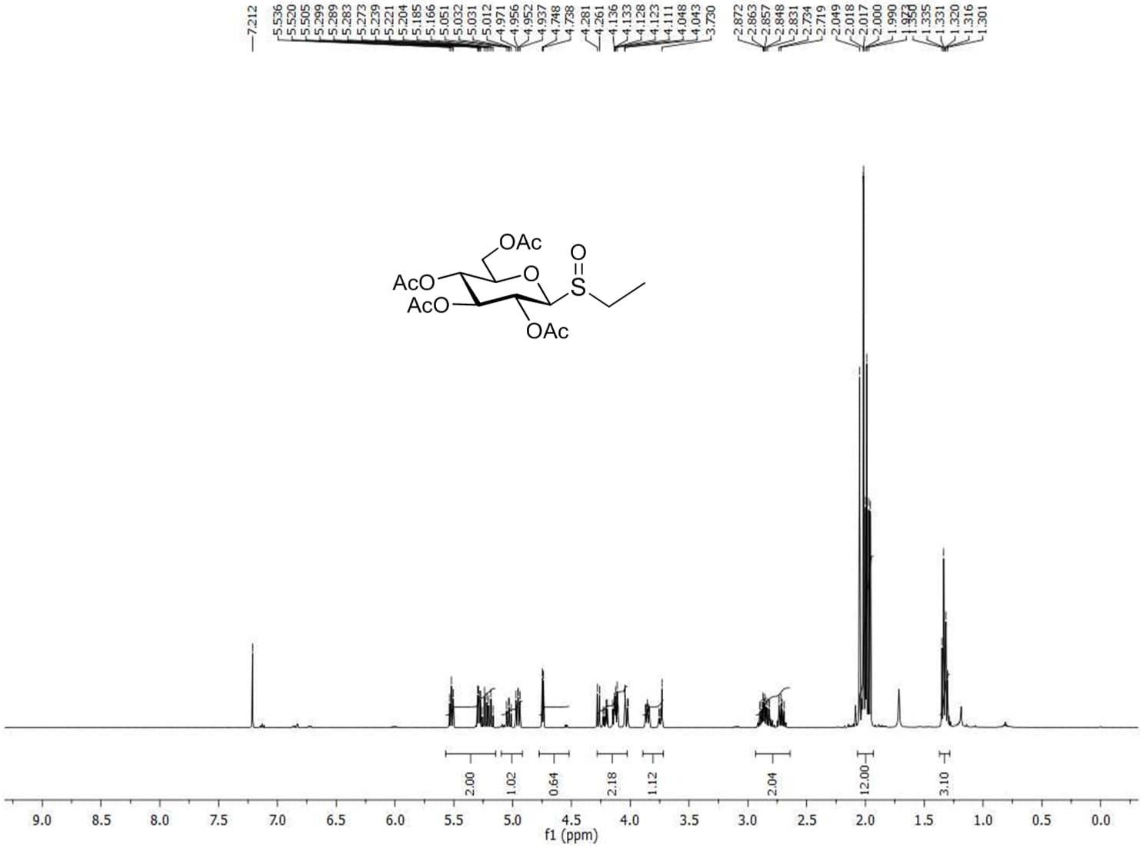

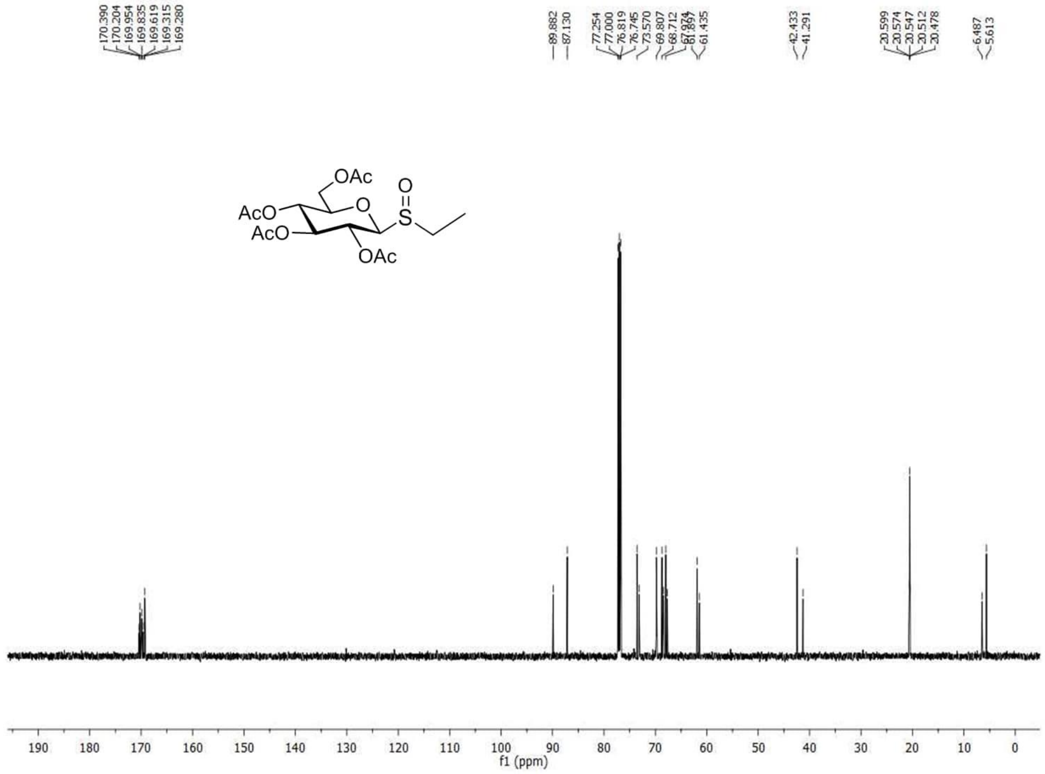

1-Deoxy-1-[(*R*/*S*)-(ethyl)sulfinyl]-2,3,4,6-tetra-*O*-benzoyl-β-D-glucopyranose (8a)

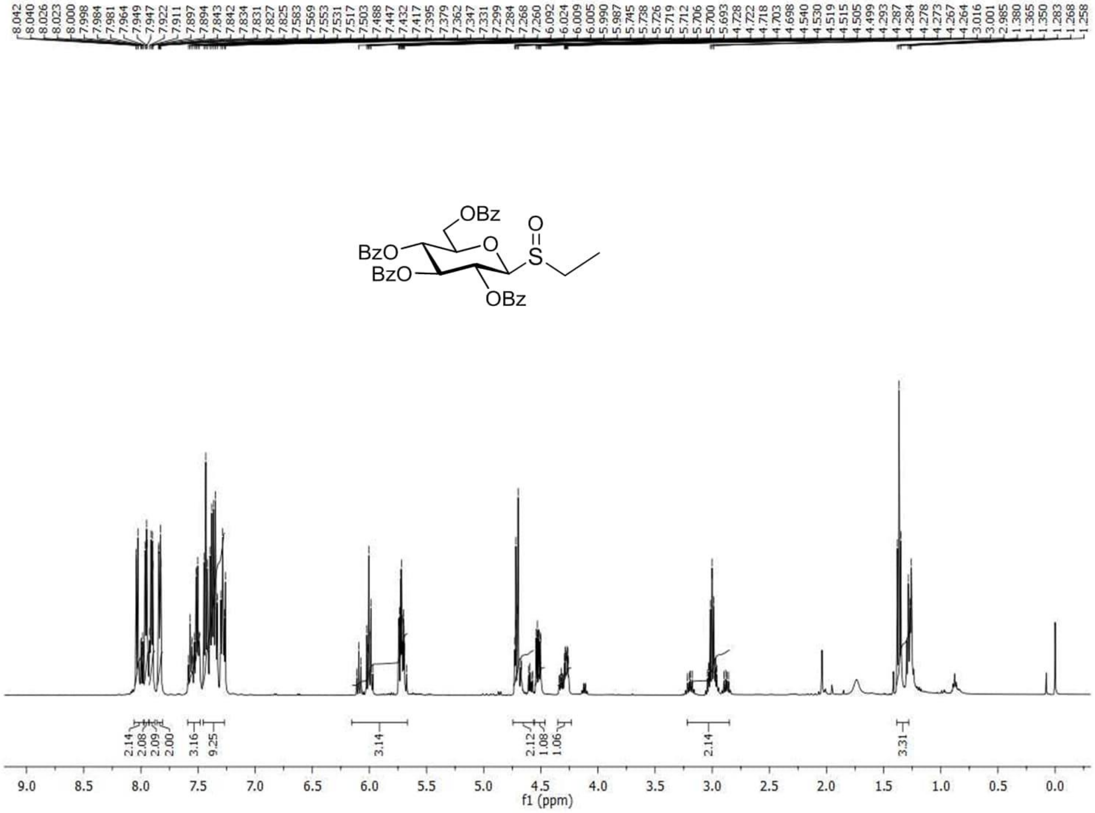

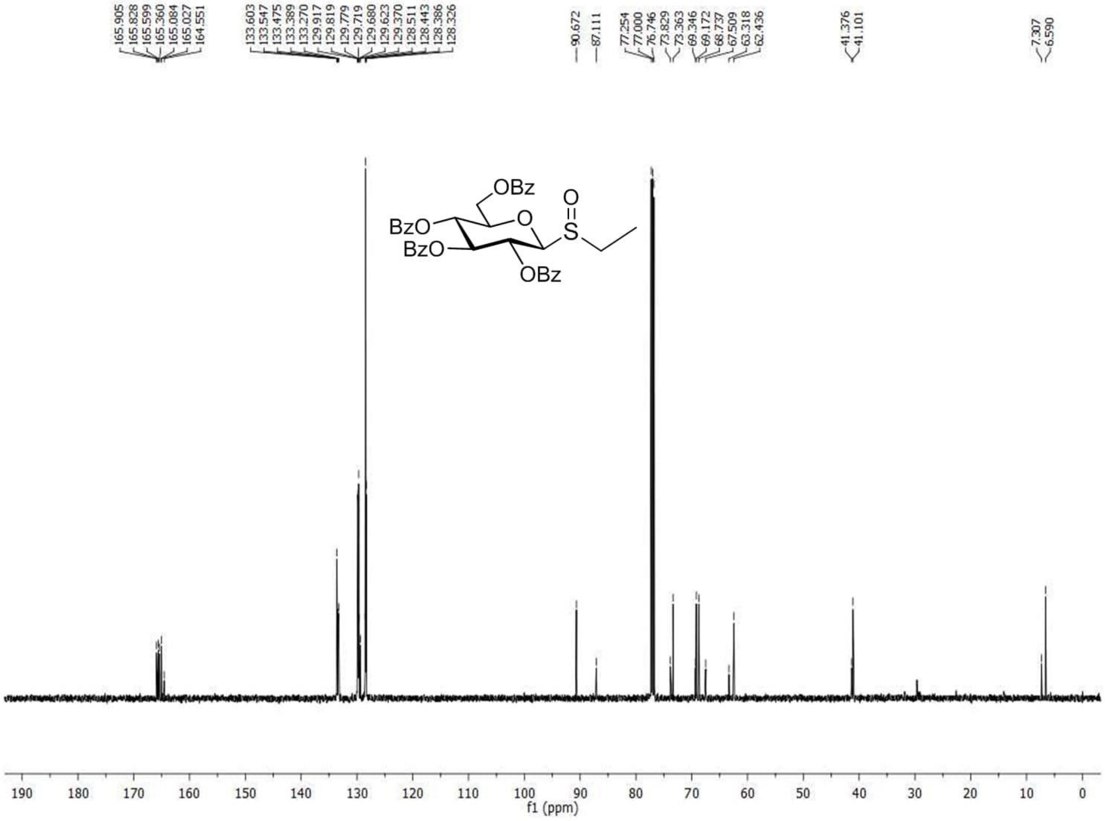

1-Deoxy-1-[(*R*/*S*)-(*n*-hexyl)sulfinyl]-2,3,4,6-tetra-*O*-acetyl-β-D-glucopyranose (9a)

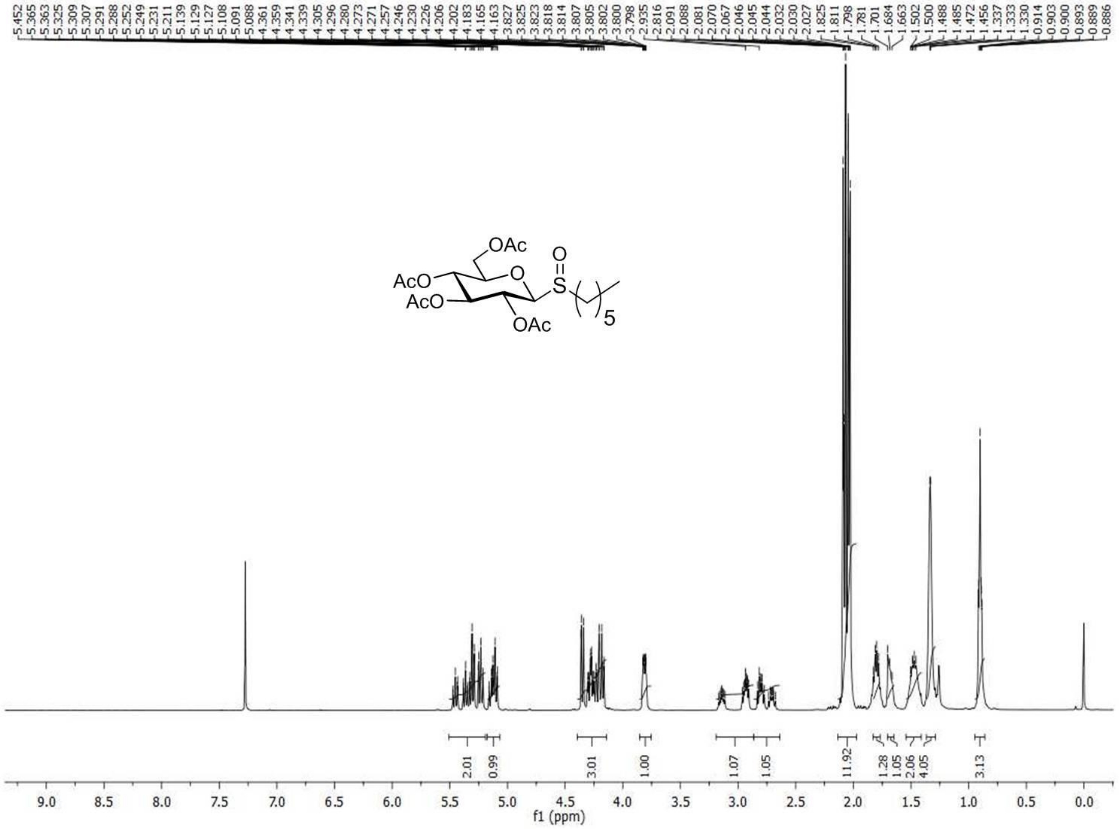

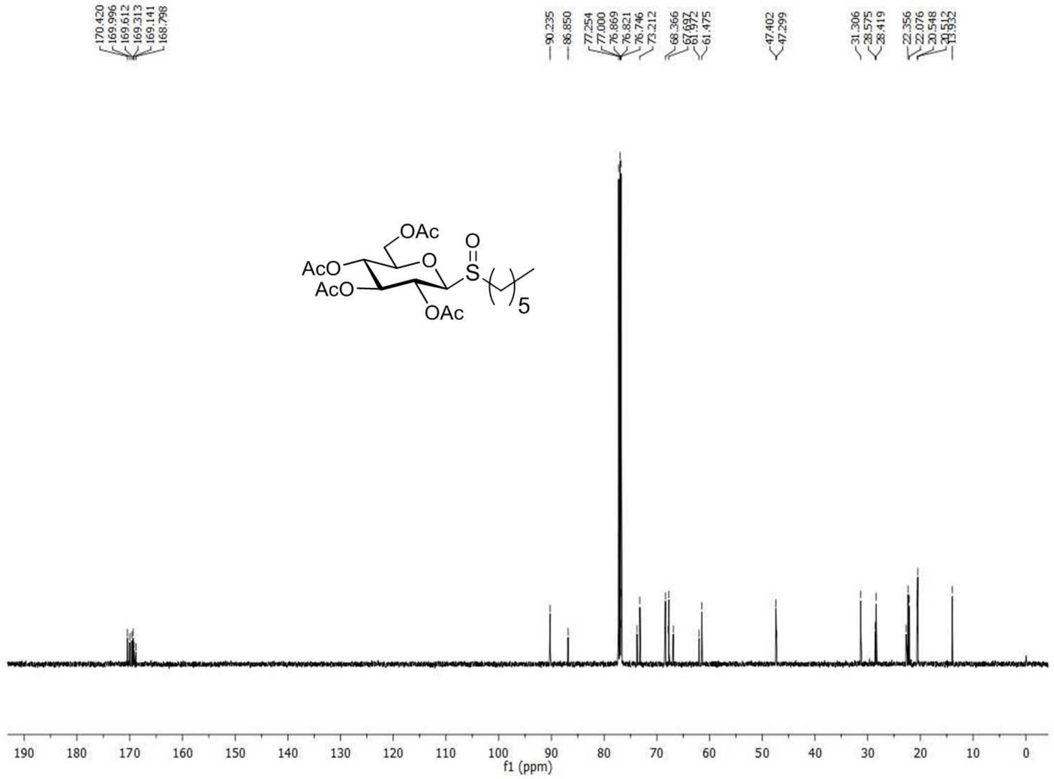

1-Deoxy-1-[(*R/S*)-(*n*-hexyl)sulfinyl]-2,3,4,6-tetra-*O*-benzoyl-β-D-glucopyranose (10a)

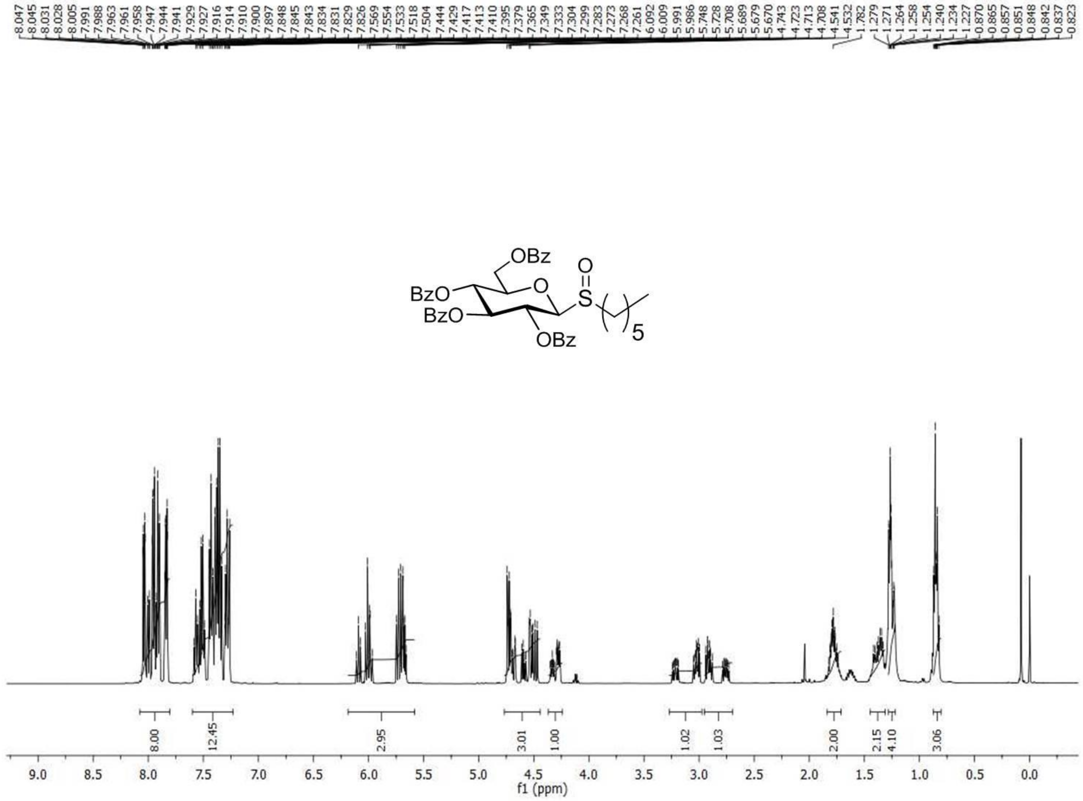

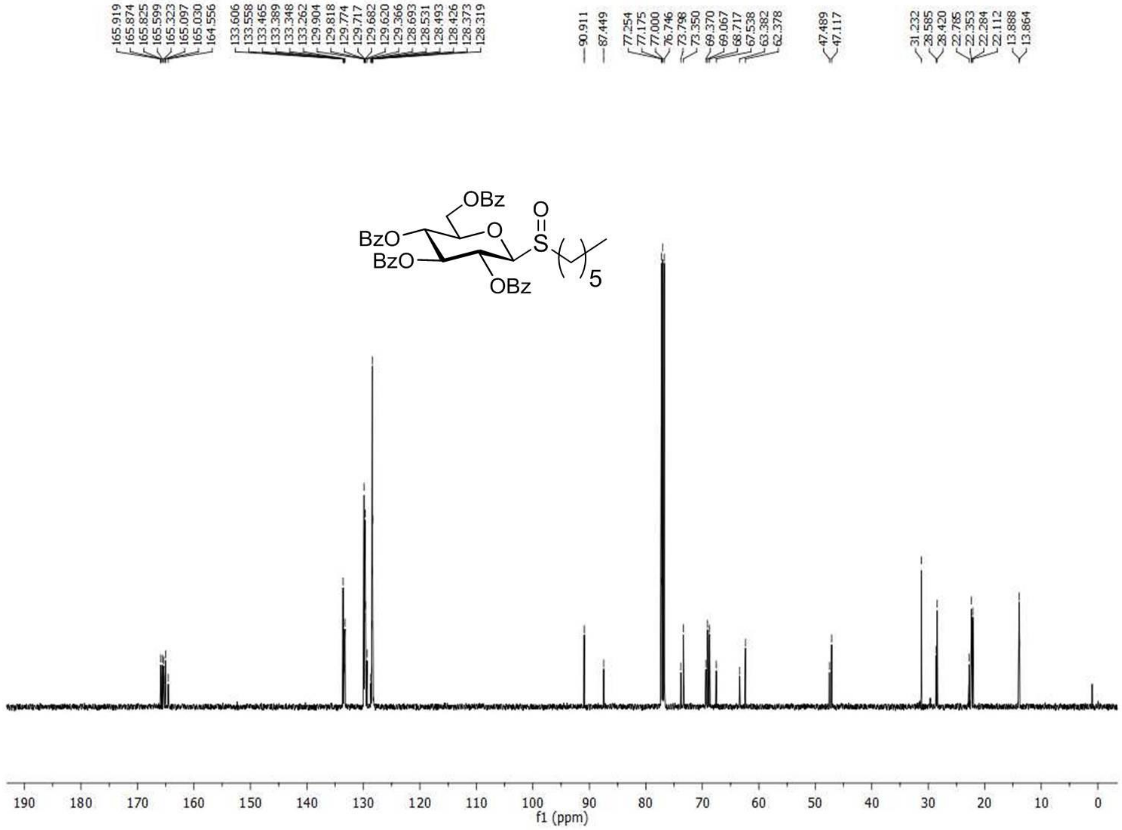

1-Deoxy-1-[(*R/S*)-(cyclohexyl)sulfinyl]-2,3,4,6-tetra-*O*-acetyl-β-D-glucopyranose (11a)

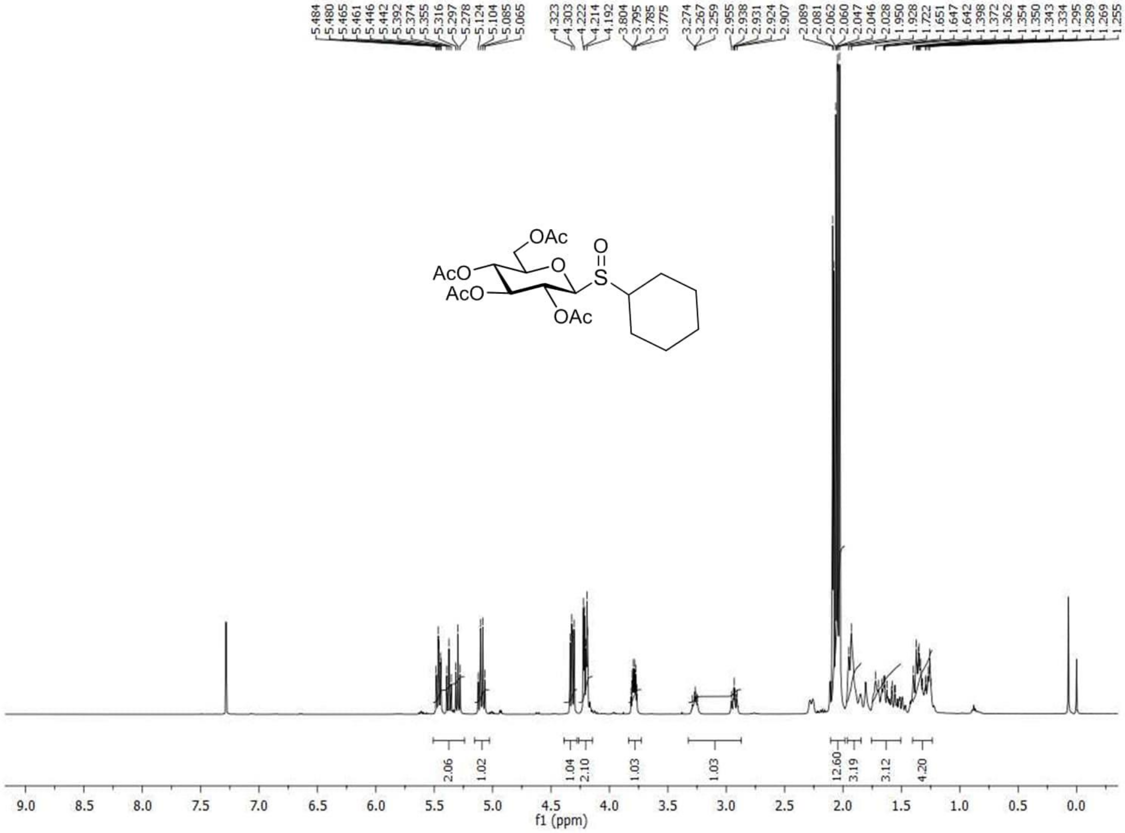

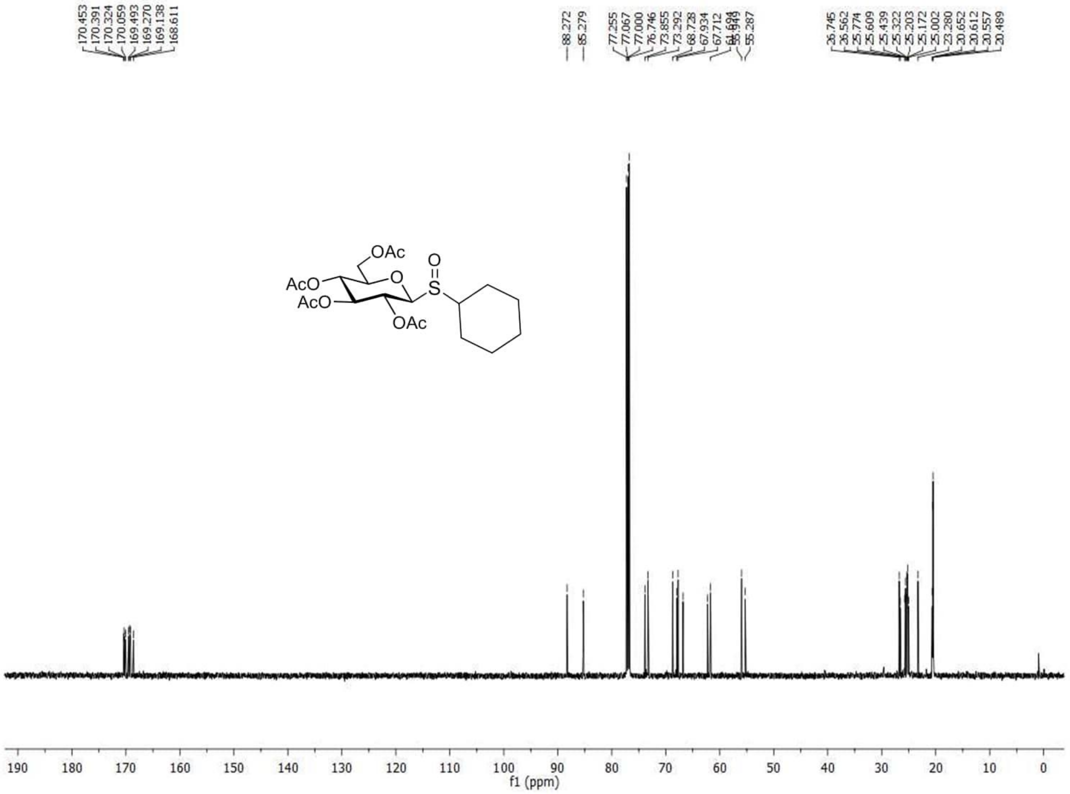

1-Deoxy-1-[(*R/S*)-(cyclohexyl)sulfinyl]-2,3,4,6-tetra-*O*-benzoyl-β-D-glucopyranose (12a)

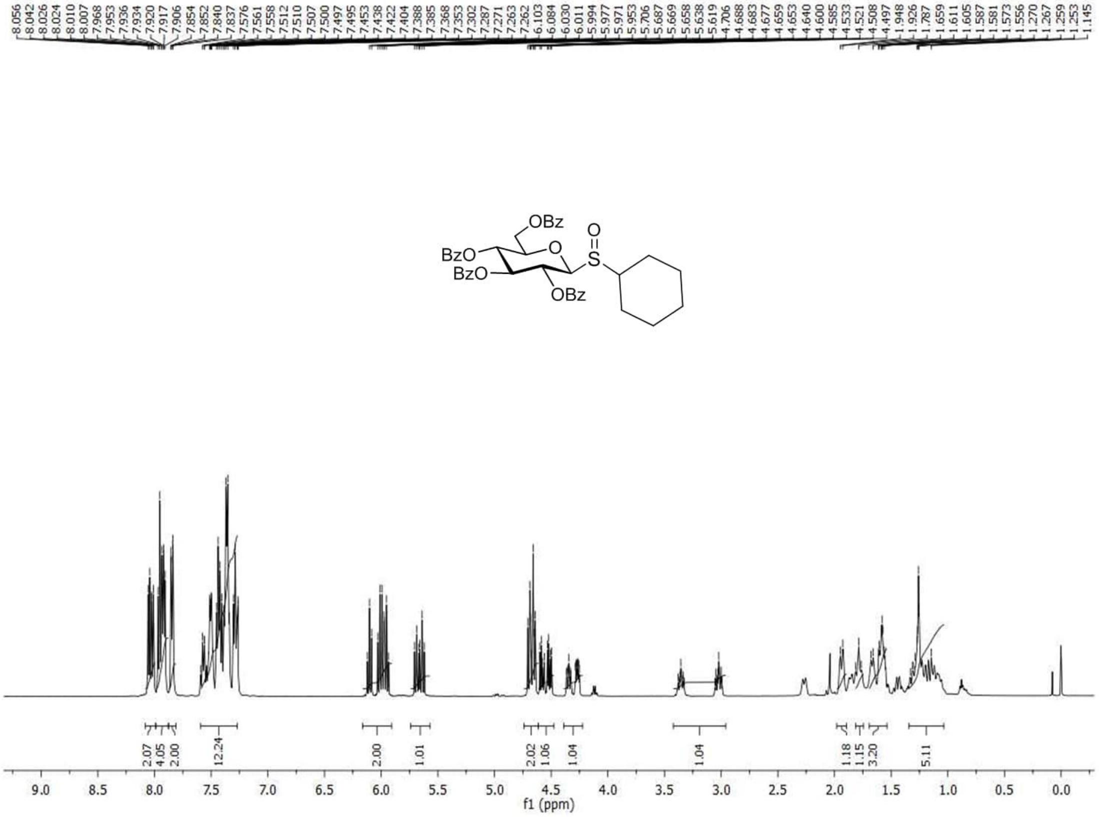

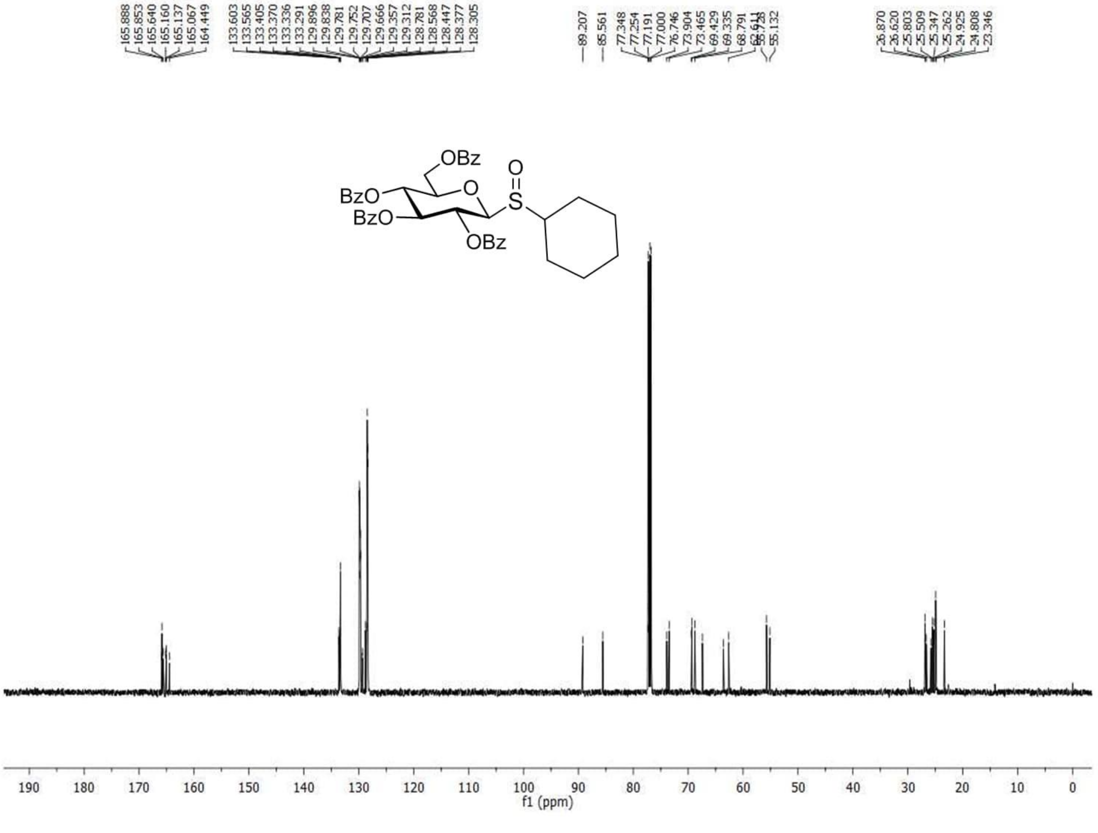

1-Deoxy-1-[(*R/S*)-(4-methylphenyl)sulfinyl]-2,3,4,6-tetra-*O*-benzyl-β-D-glucopyranose (13a)

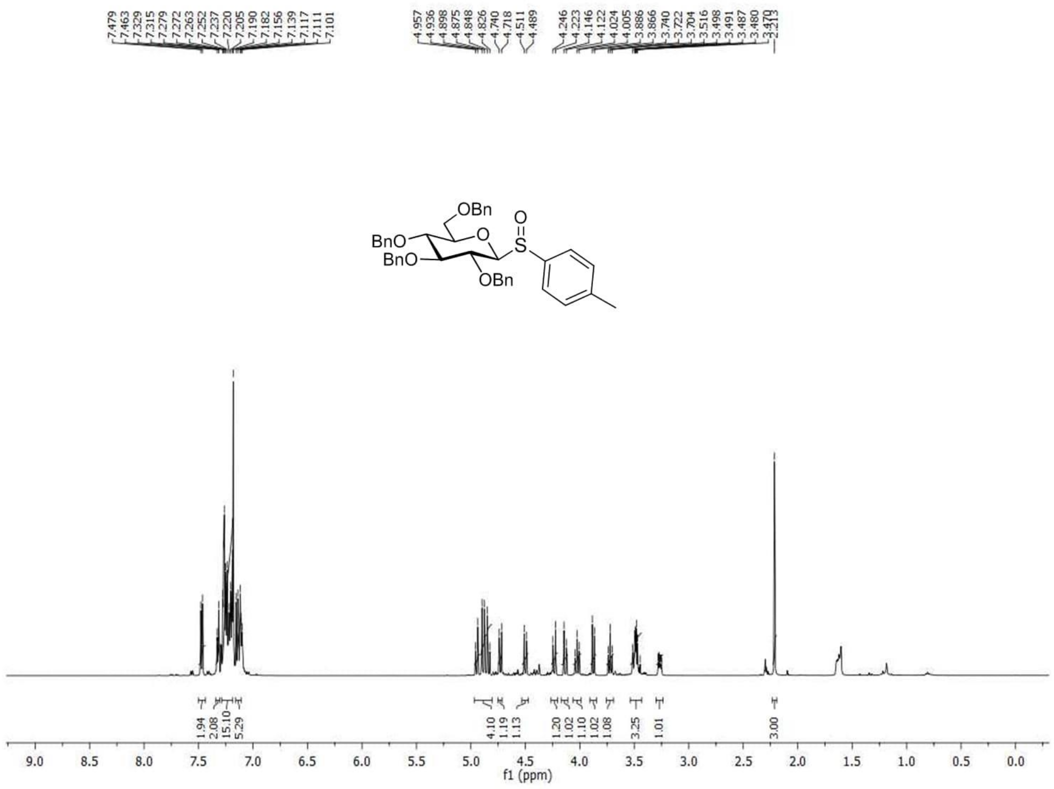

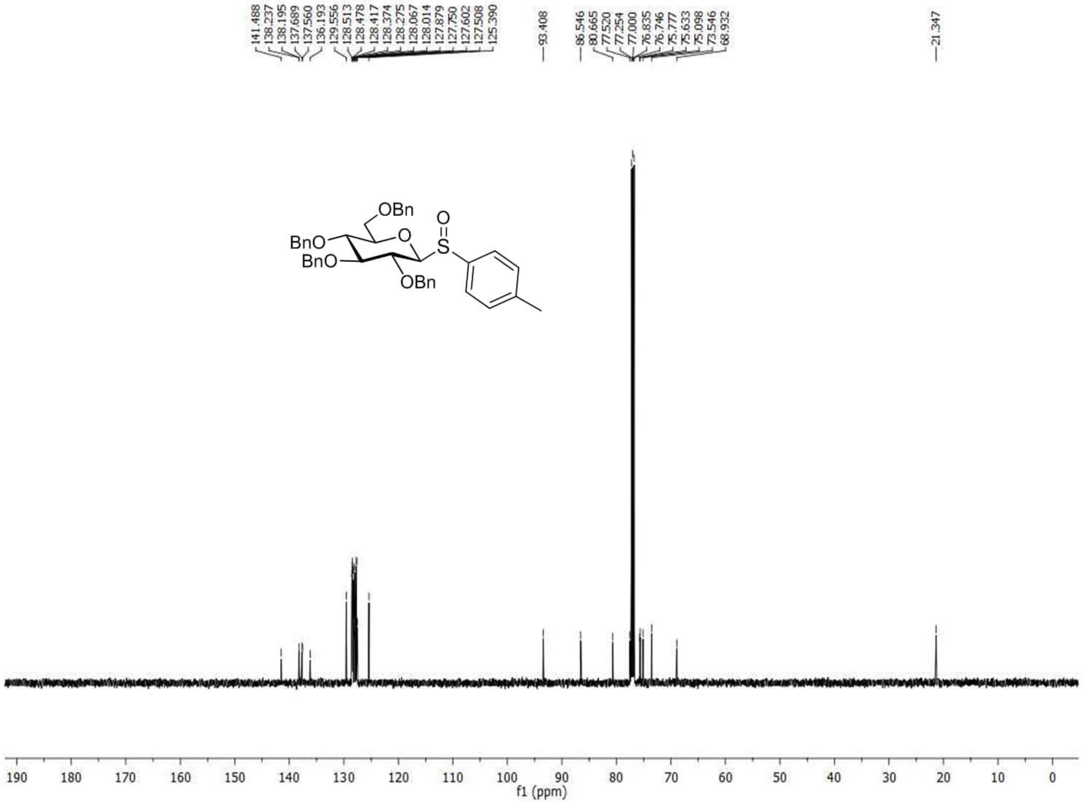

1-Deoxy-1-[(*R*/*S*)-(4-methylphenyl)sulfinyl]-2,3-di-*O*-allyl-4,6-di-*O*-acetyl-β-D-glucoopyranose (14a)

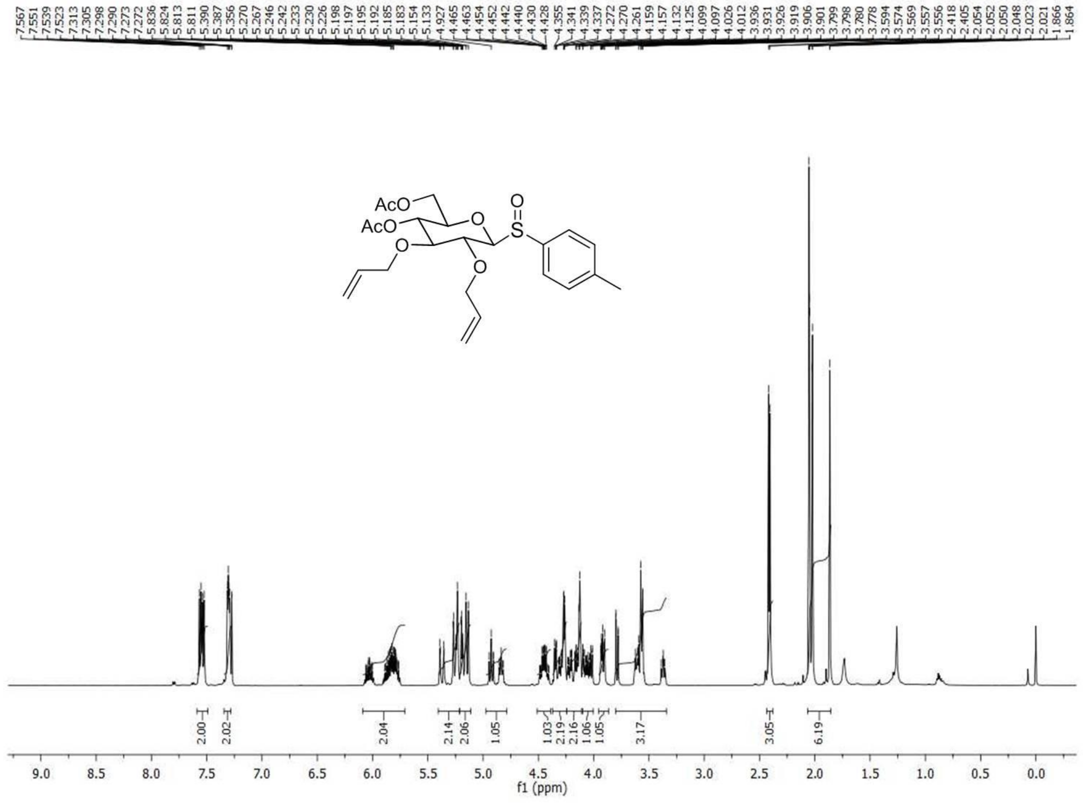

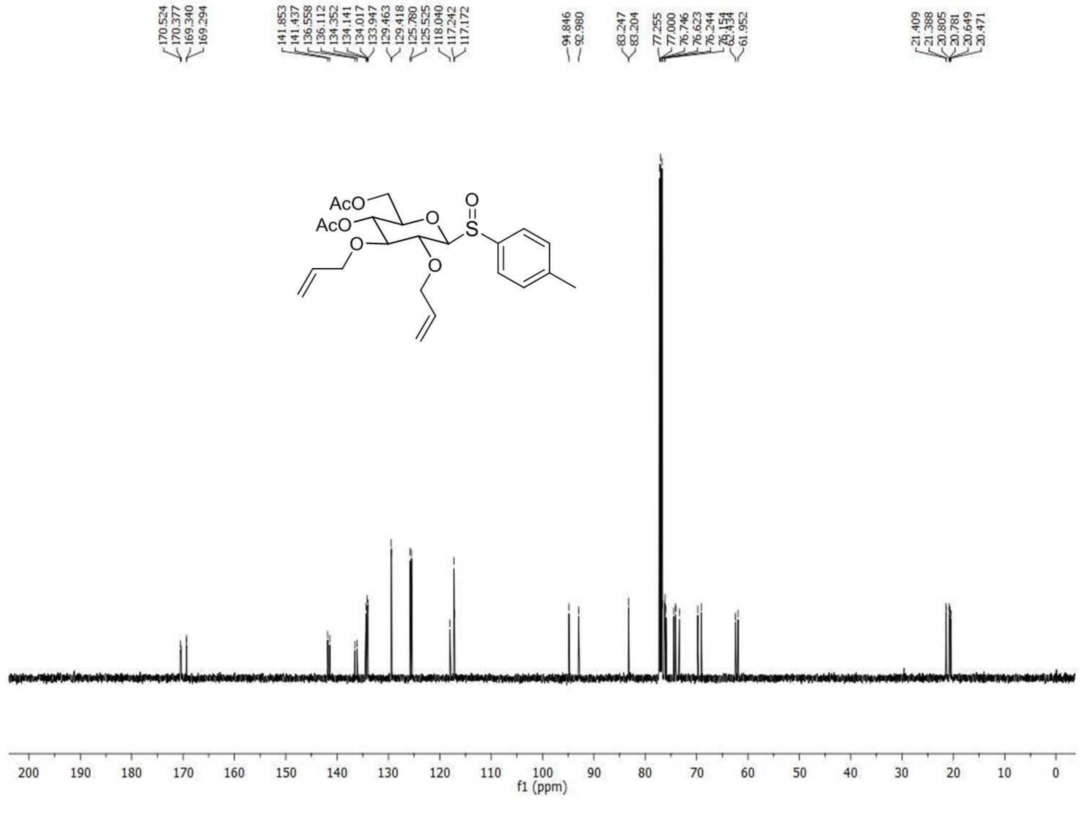

1-Deoxy-1-[(*R*/*S*)-(phenyl)sulfinyl]-2-phthalimido-3,4,6-tri-*O*-acetyl-β-D-glucoopyranose (15a)

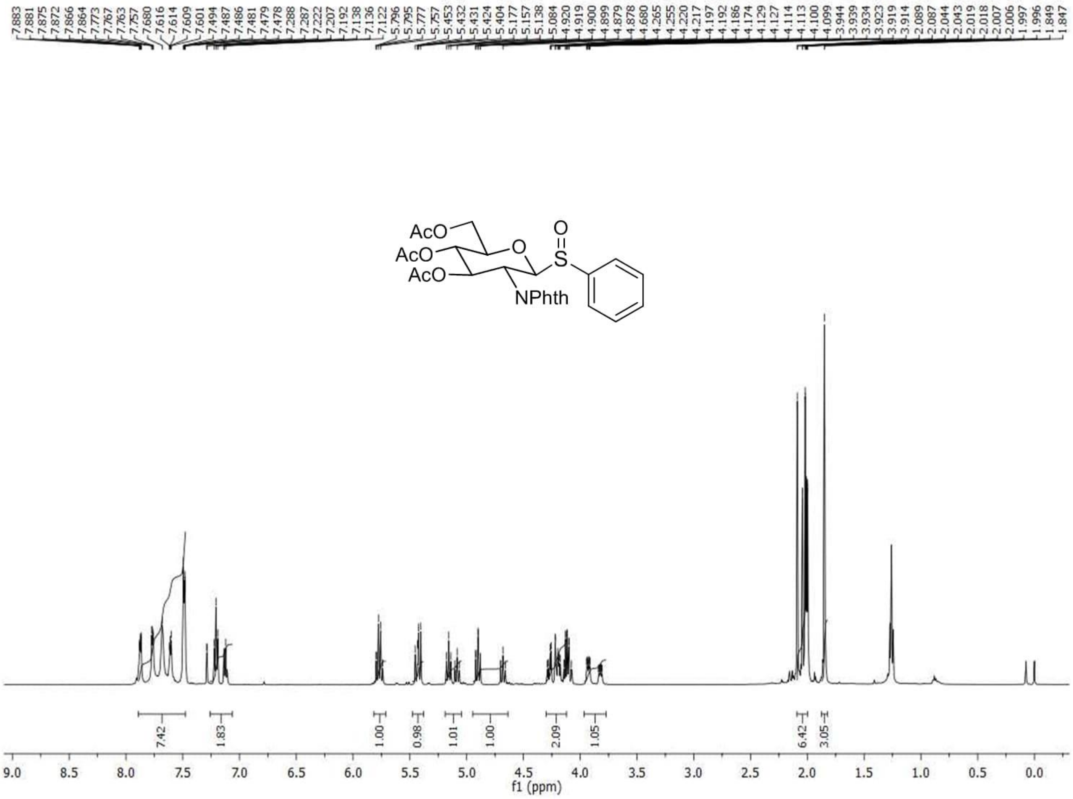

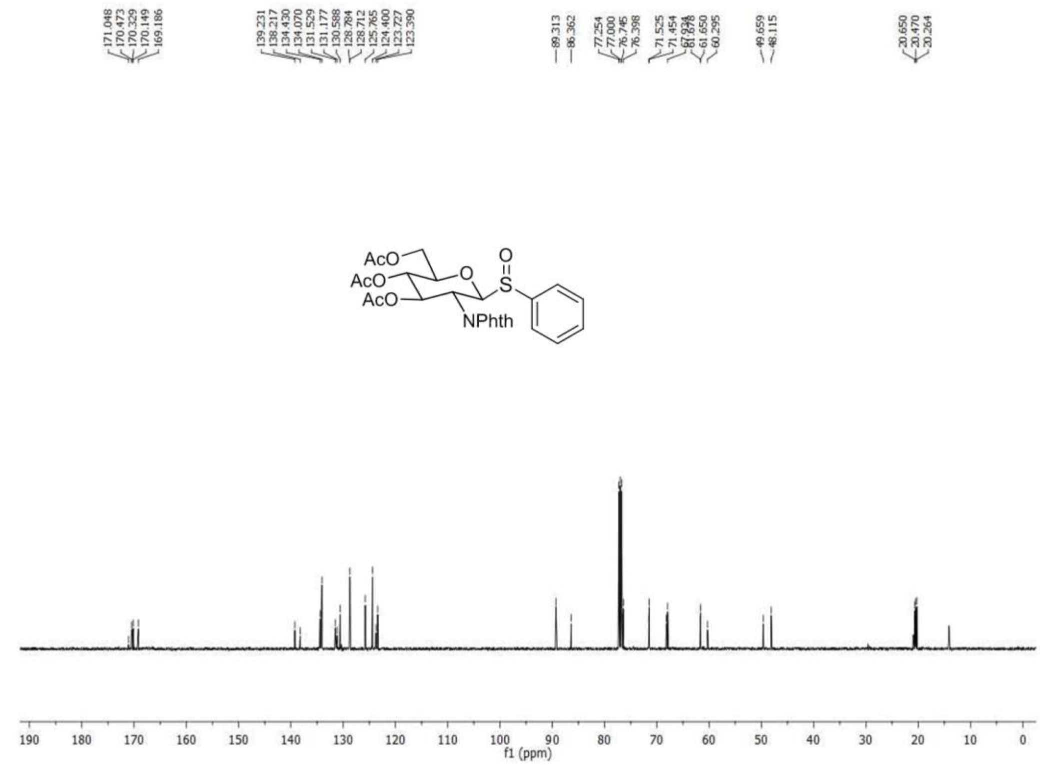

1-Deoxy-1-[(*R*/*S*)-(4-methylphenyl)sulfinyl]-2,3,4,6-tetra-*O*-acetyl-β-D-galatopyranose (16a)

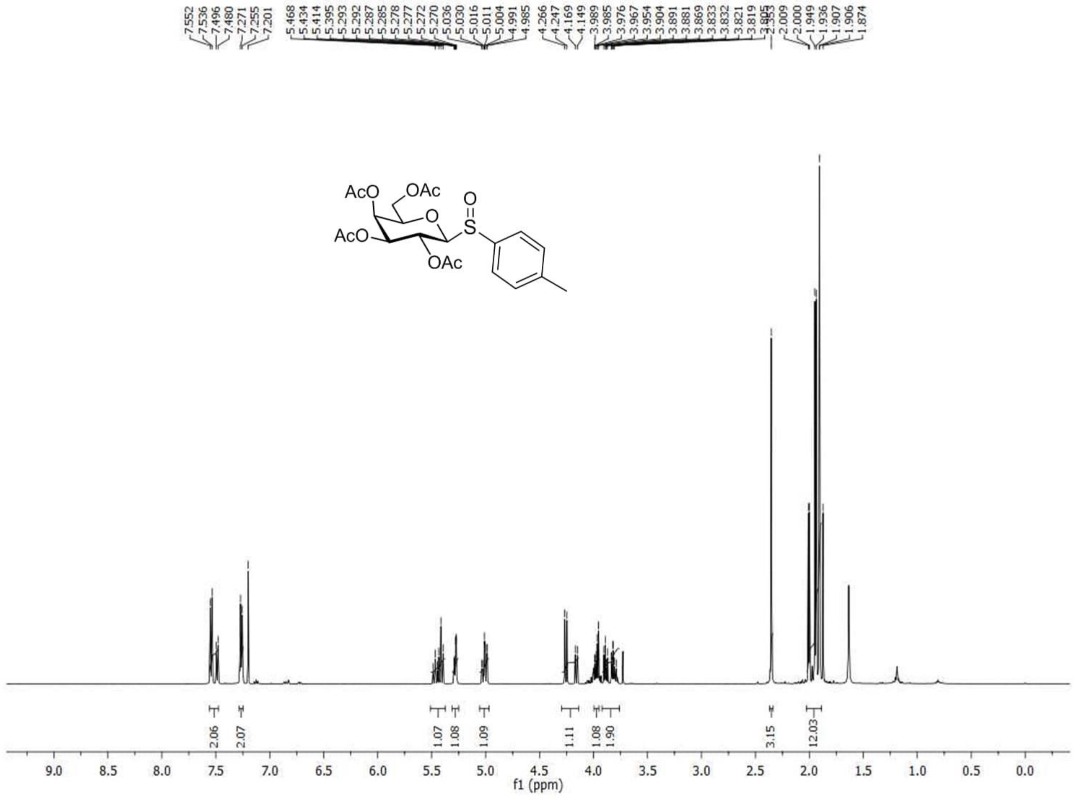

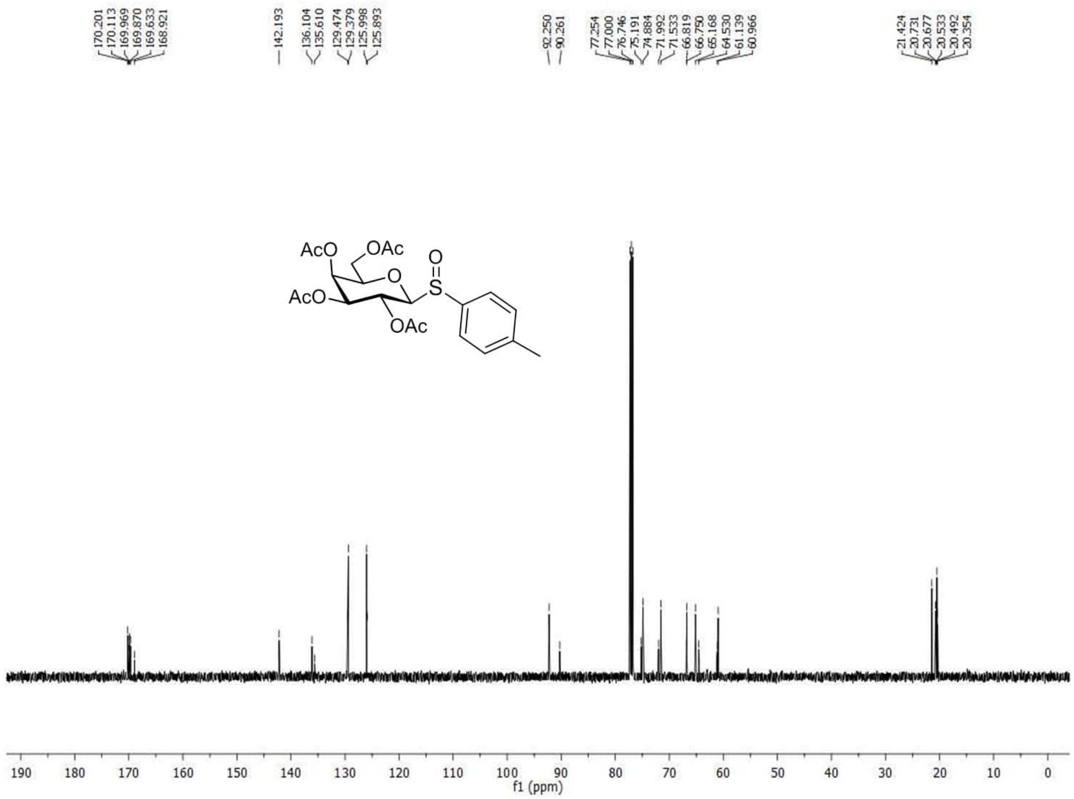

1-Deoxy-1-[(*R*/*S*)-(4-methylphenyl)sulfinyl]-2,3,4,6-tetra-*O*-benzoyl-β-D-galatopyranose (17a)

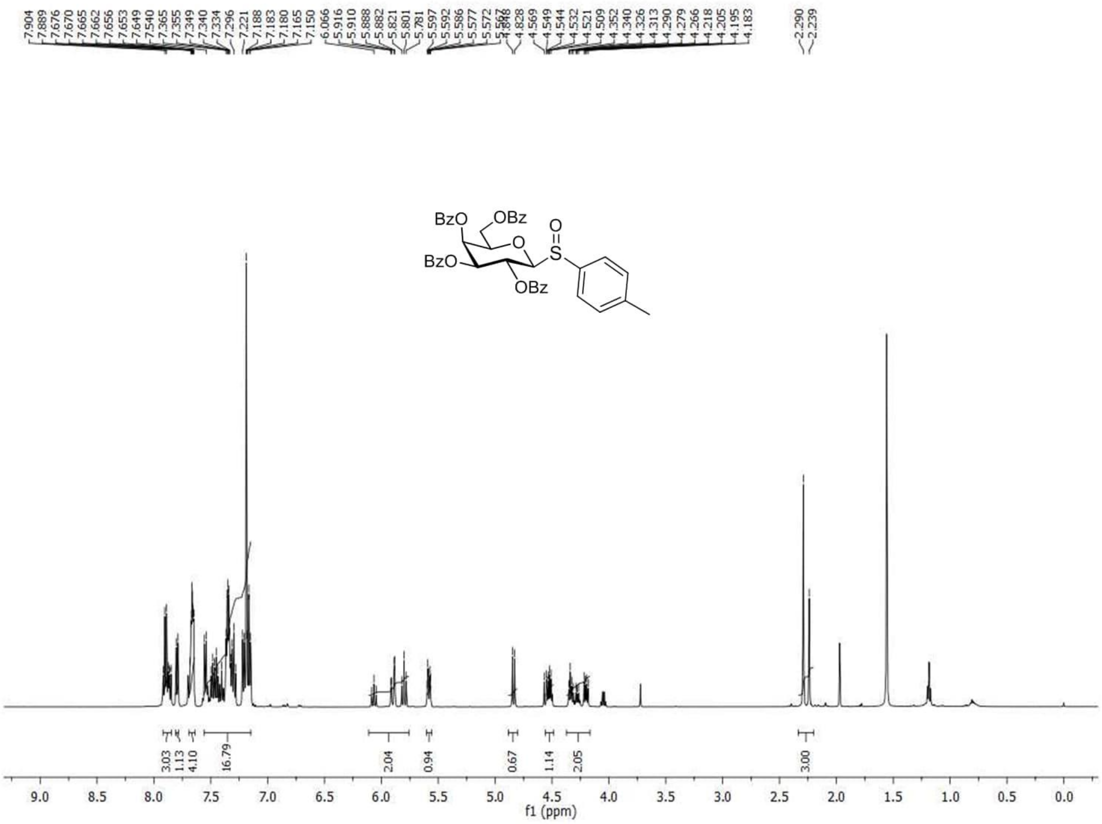

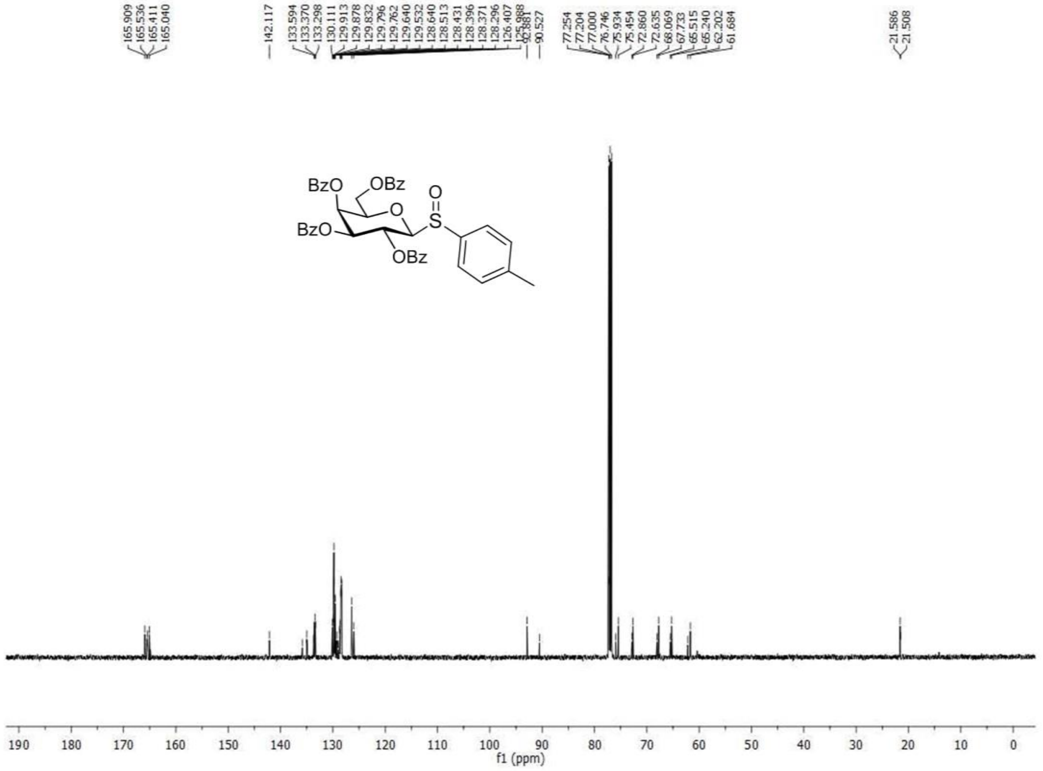

1-Deoxy-1-[(*R*/*S*)-(phenyl)sulfinyl]-2,3,4,6-tetra-*O*-acetyl-α-D-mannopyranose (18a)

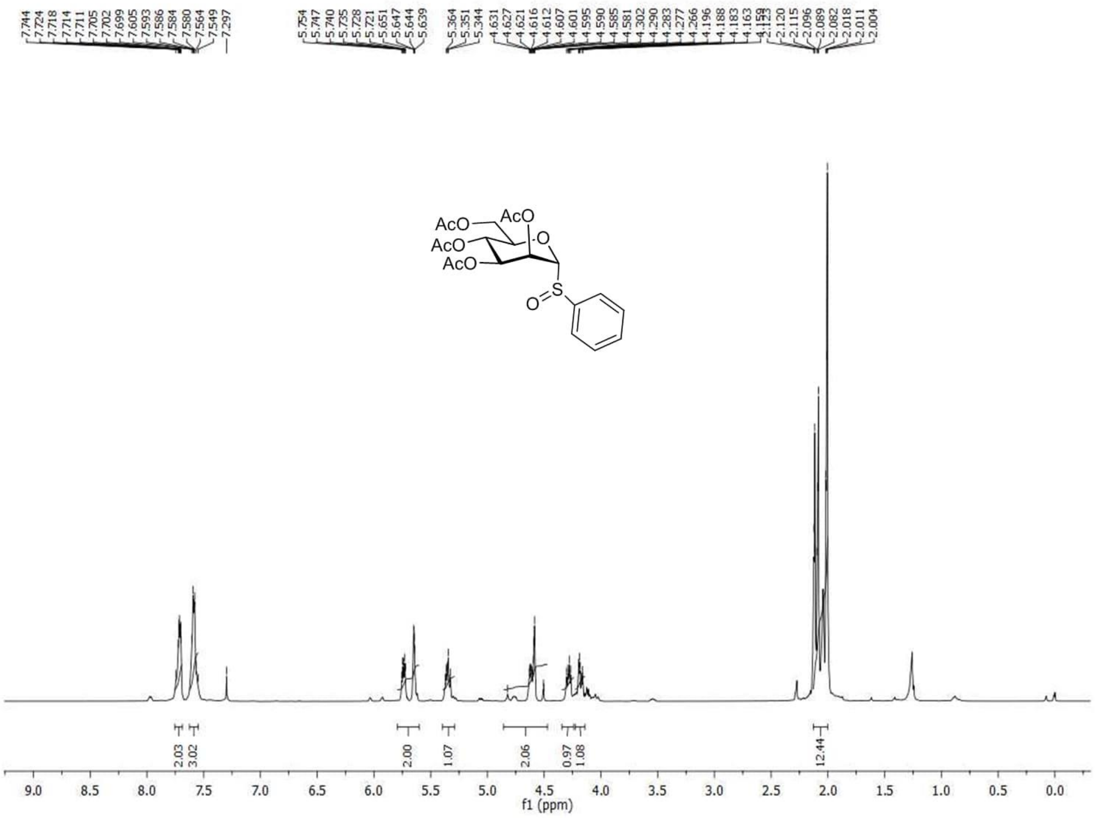

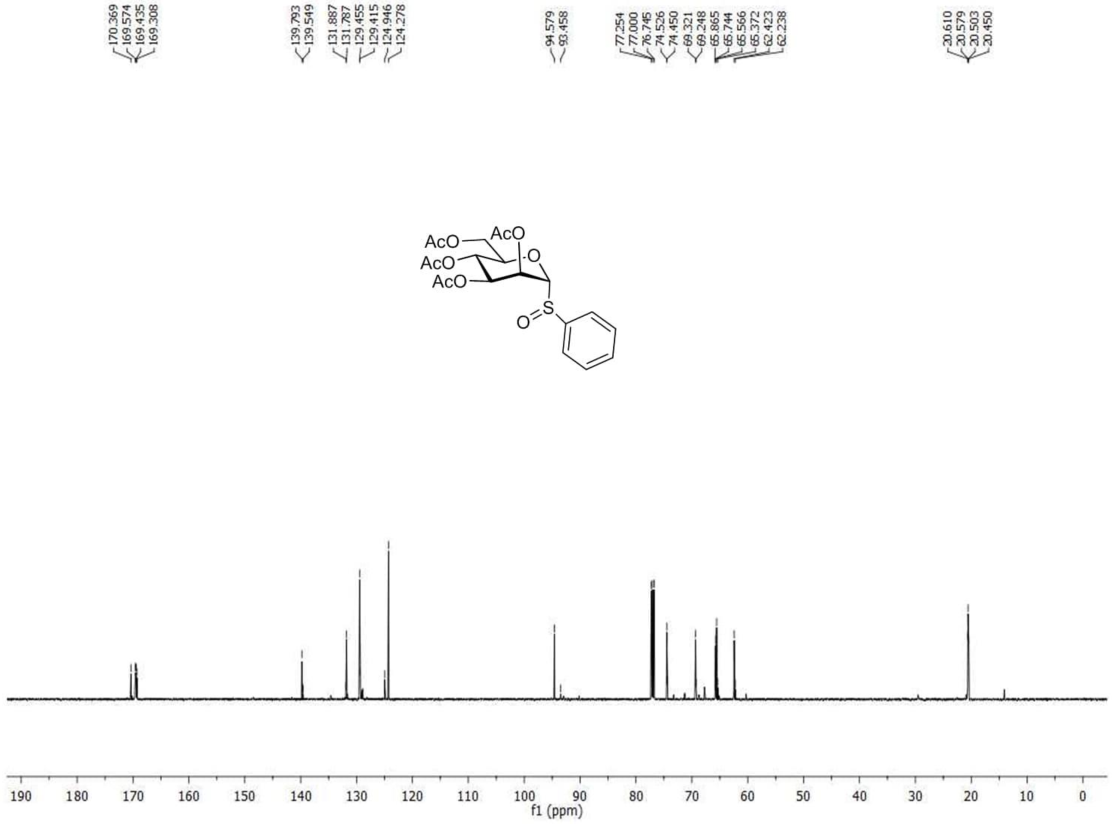

1-Deoxy-1-[(*R*/*S*)-(phenyl)sulfinyl]-2,3,4,6-tetra-*O*-benzoyl-α-D-mannopyranose (19a)

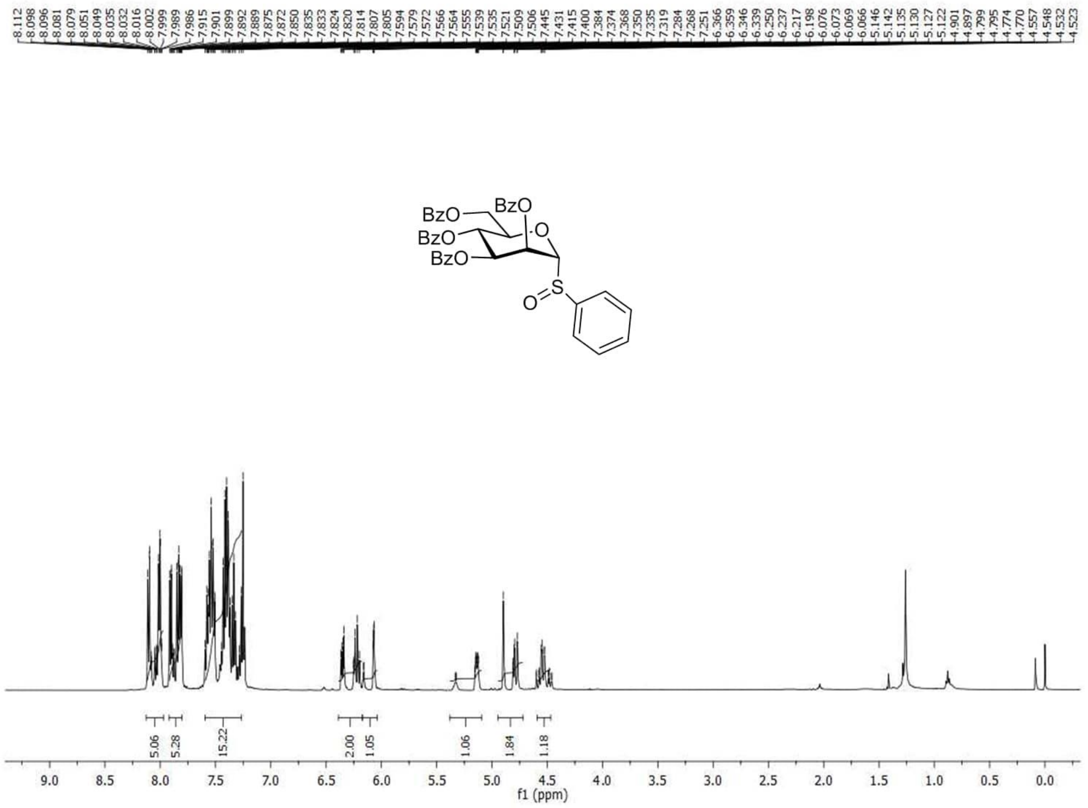

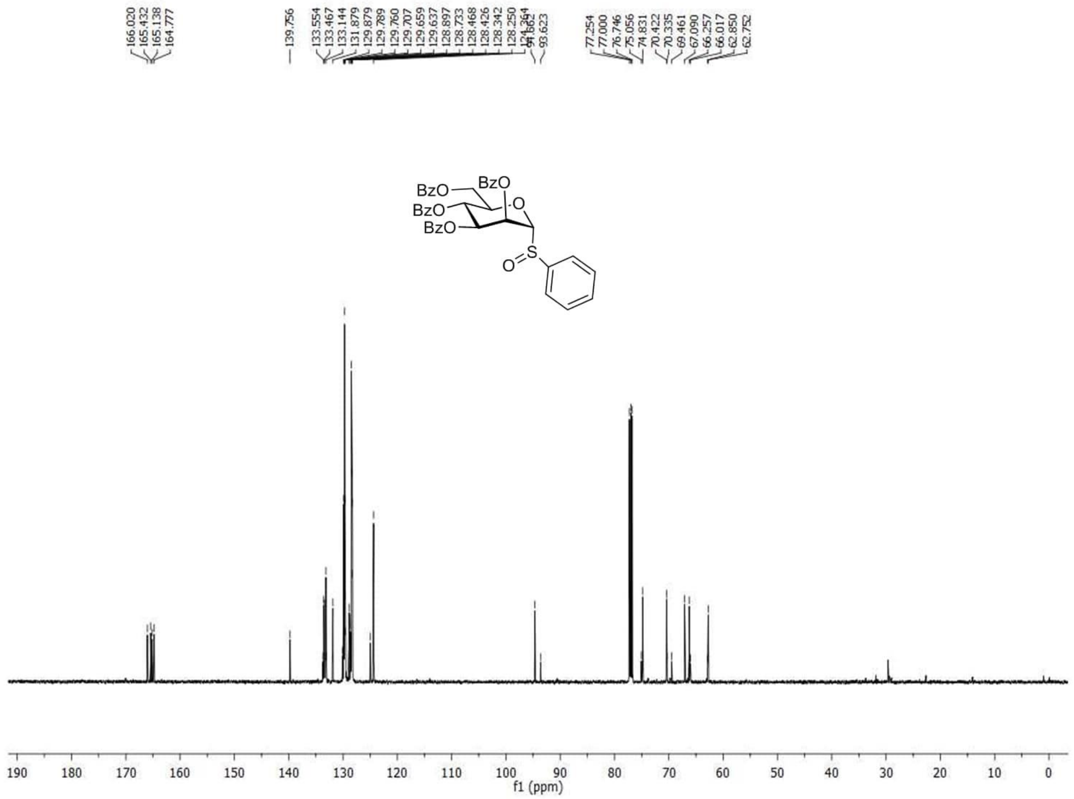

^{1}H and ^{13}C spectra of sulfones

Phenylsulfonyl 2,3,4,6-tetra-*O*-acetyl-1-deoxy-β-D-glucopyranoside (1b)

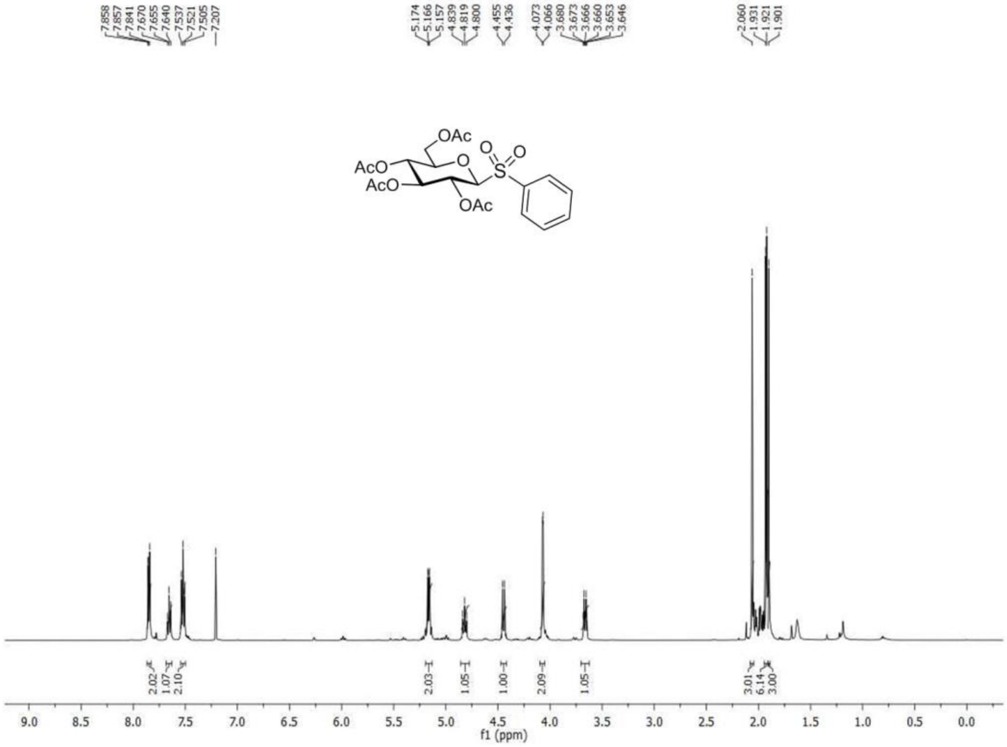

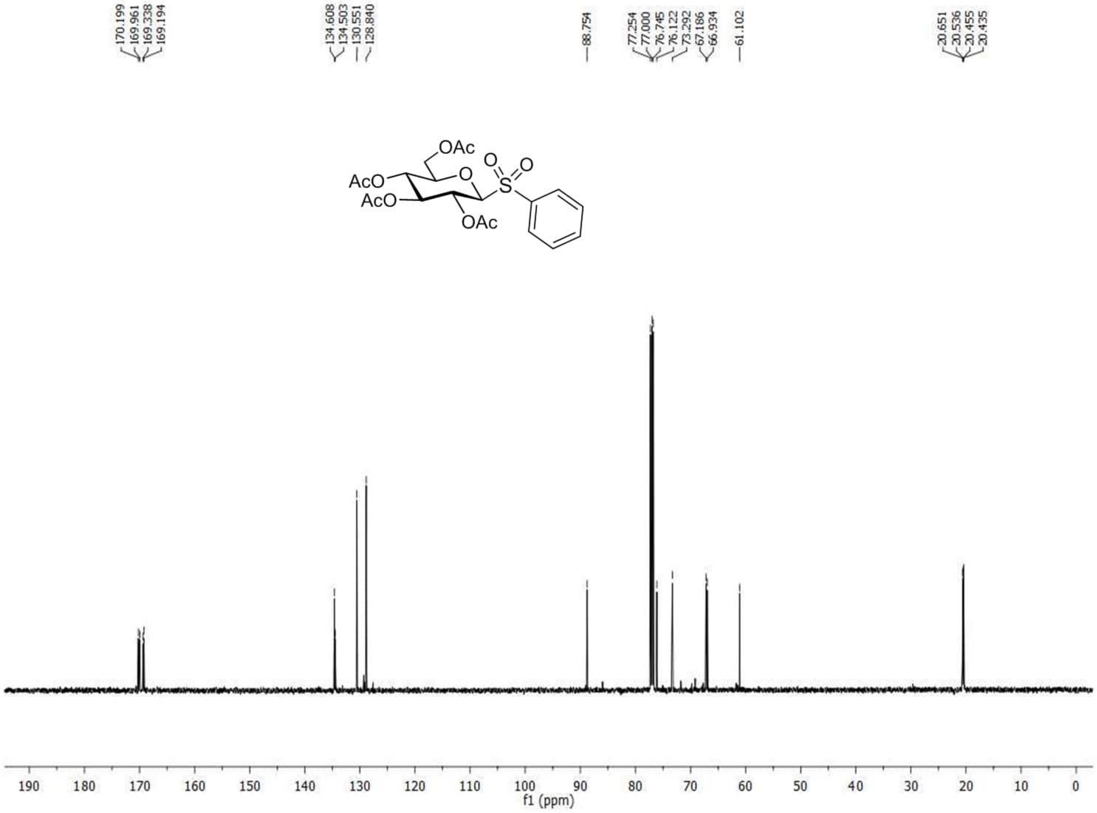

Phenylsulfonyl 2,3,4,6-tetra-*O*-benzoyl-1-deoxy-β-D-glucopyranoside (2b)

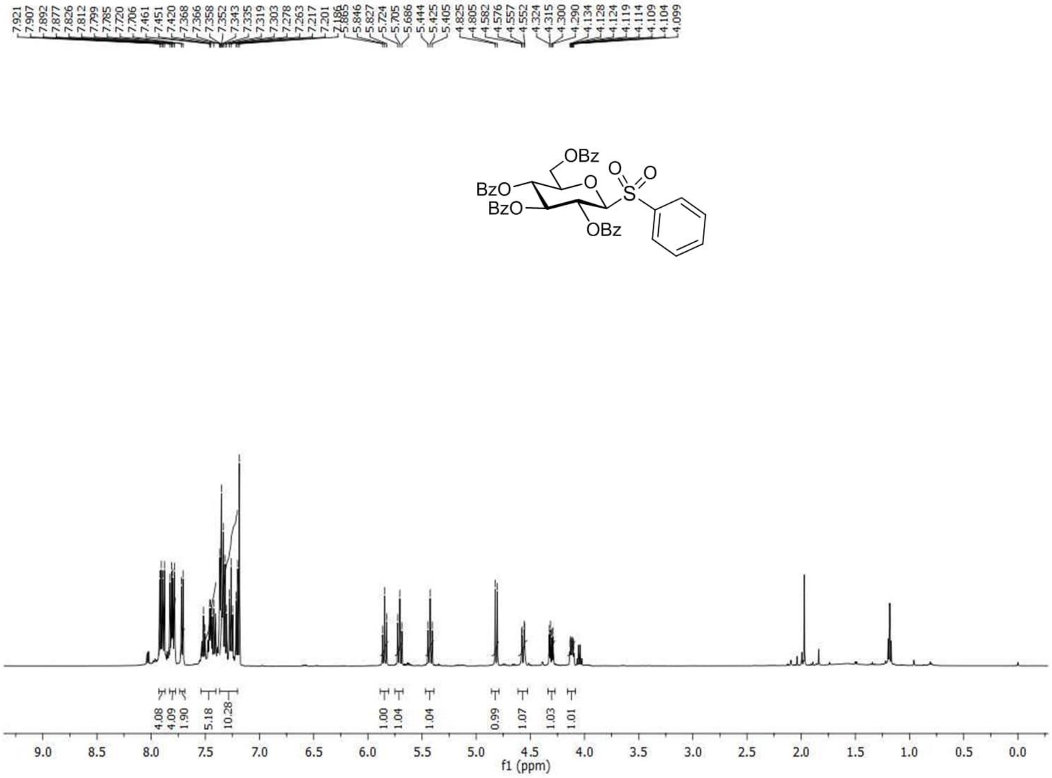

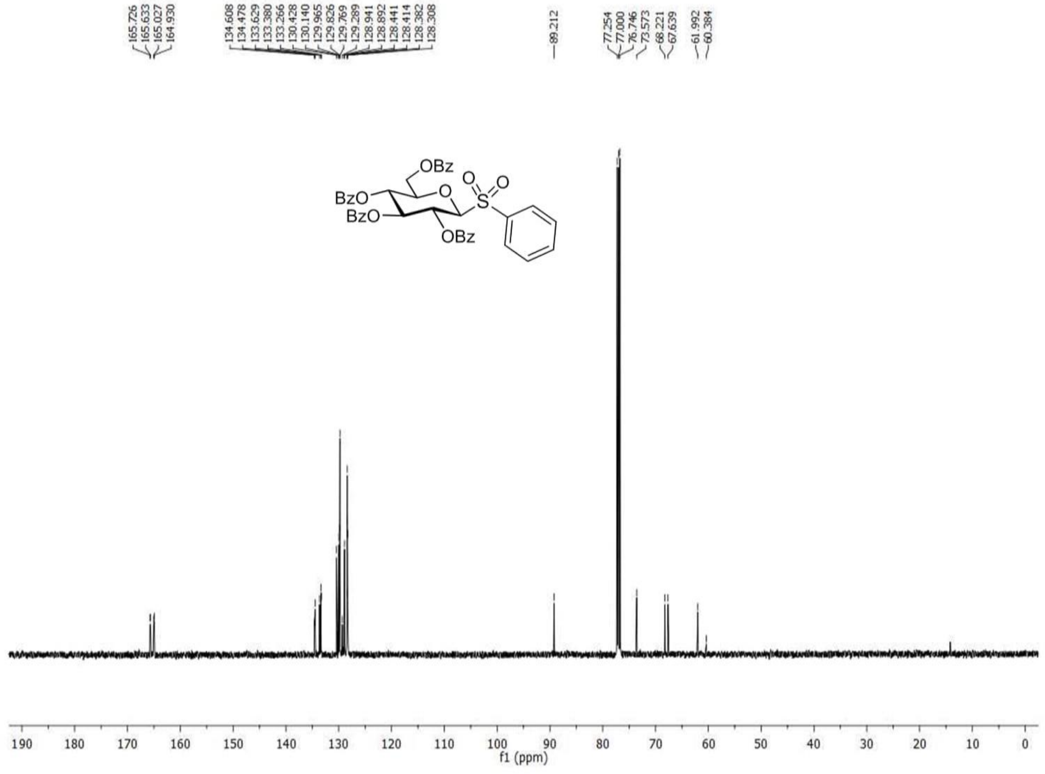

p-Methylphenylsulfonyl 2,3,4,6-tetra-O-acetyl-1-deoxy-β-D-glucopyranoside (3b)

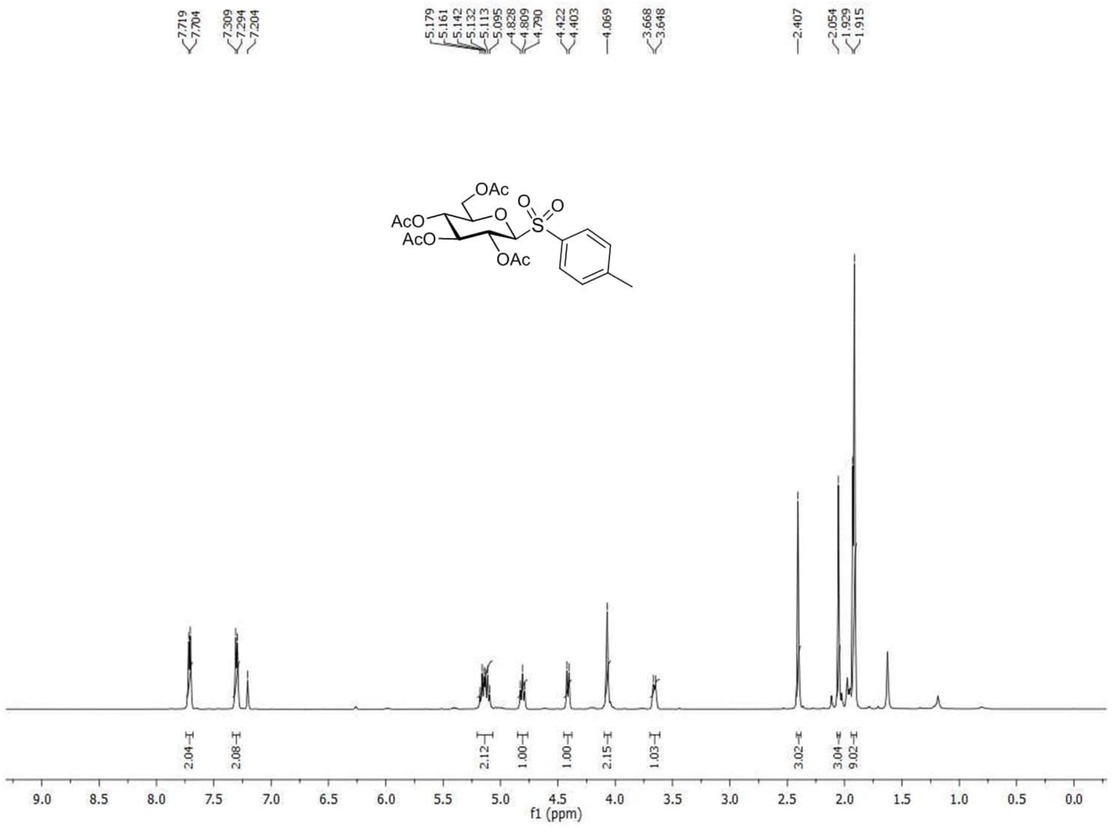

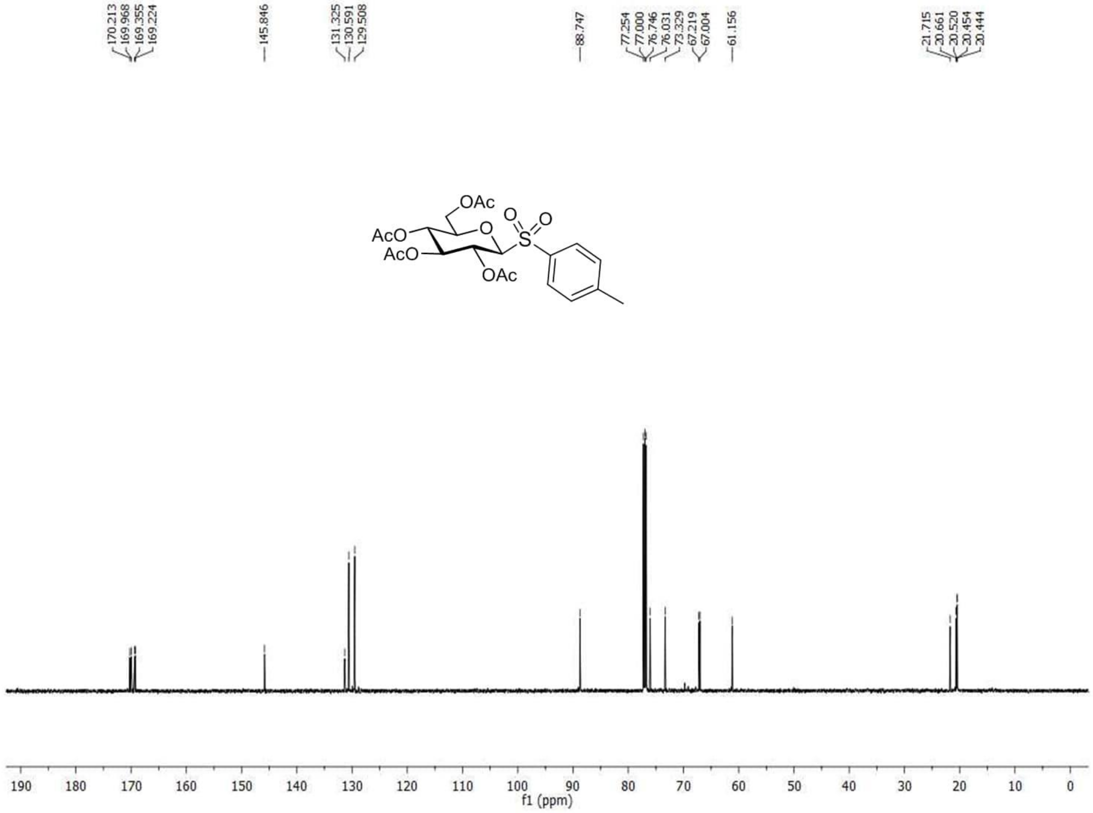

***p*-Methylphenylsulfonyl 2,3,4,6-tetra-*O*-benzoyl-1-deoxy-β-D-glucopyranoside (4b)**

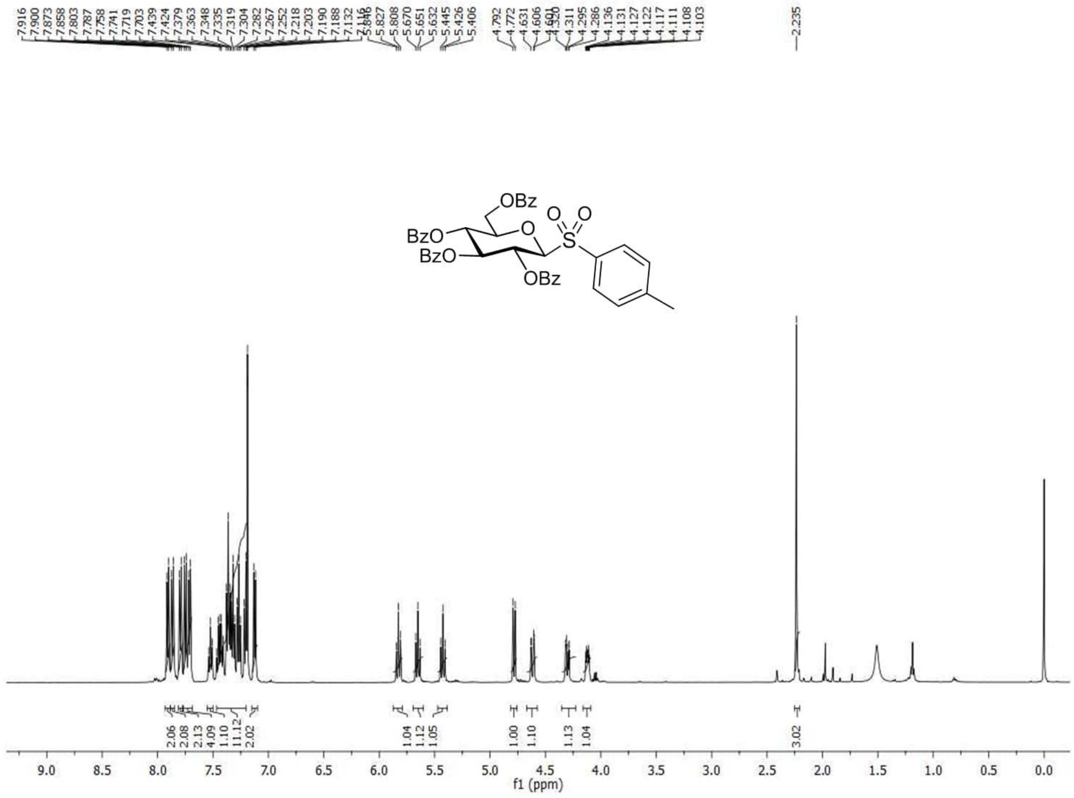

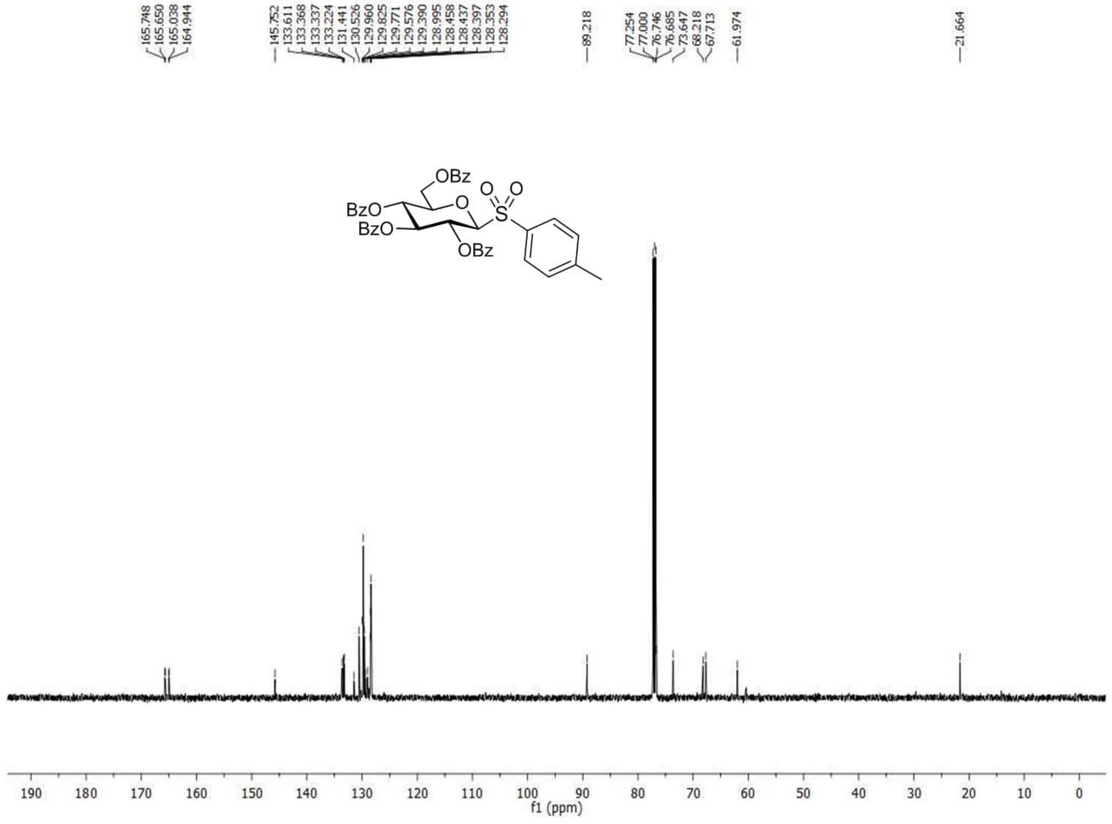

Benzylsulfonyl 2,3,4,6-tetra-*O*-acetyl-1-deoxy-β-D-glucopyranoside (5b)

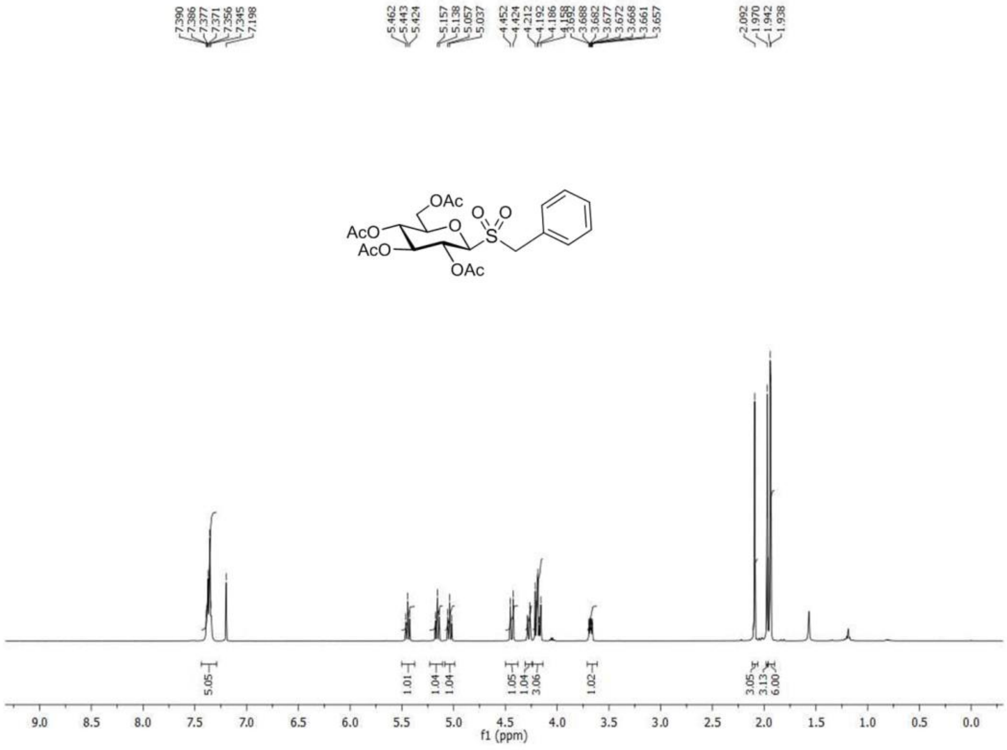

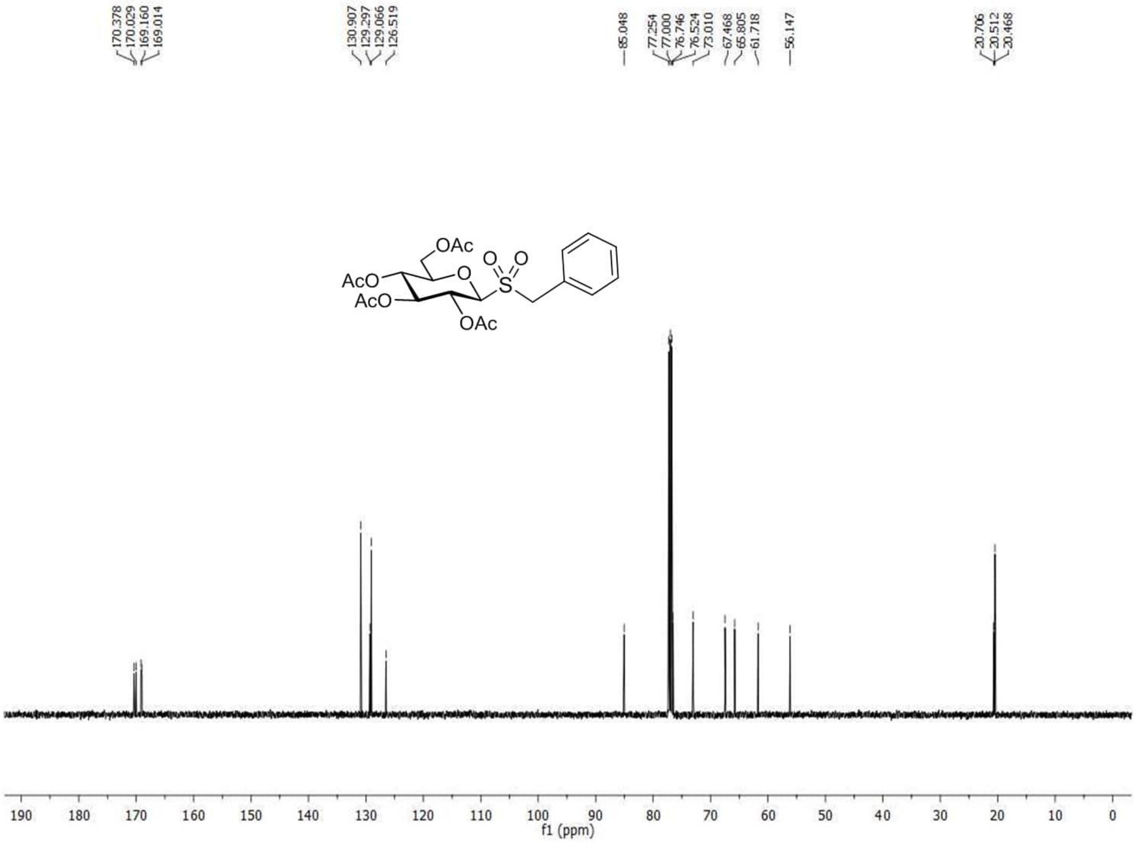

Benzylsulfonyl 2,3,4,6-tetra-*O*-benzoyl-1-deoxy-β-D-glucopyranoside (6b)

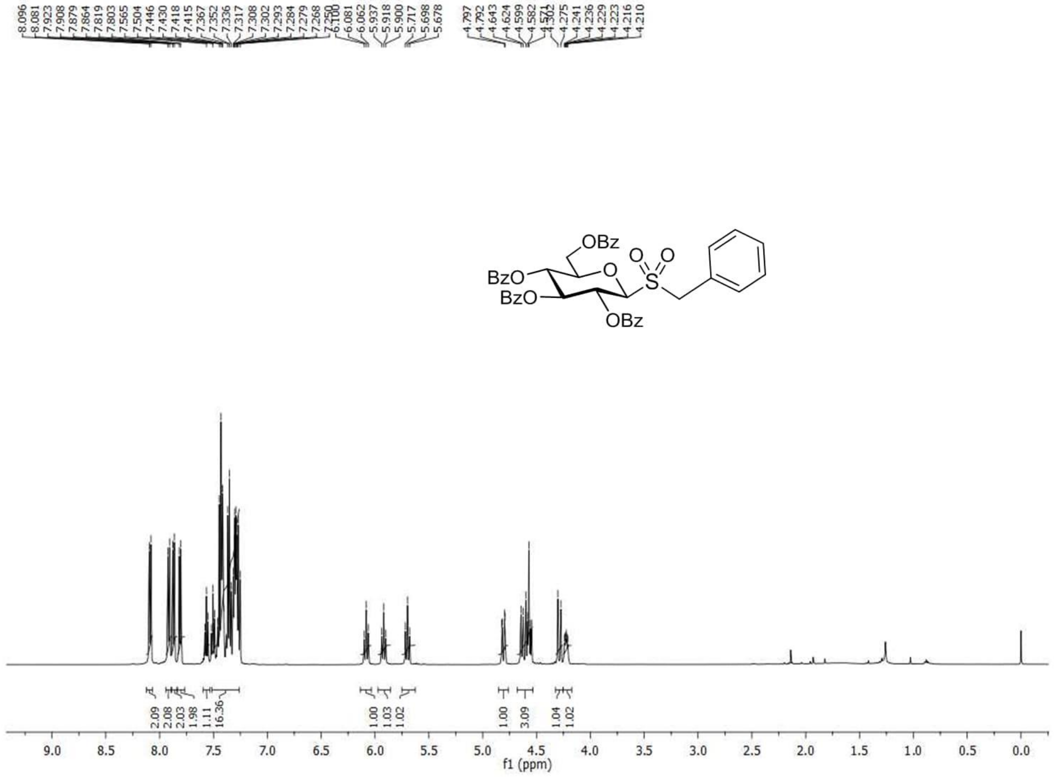

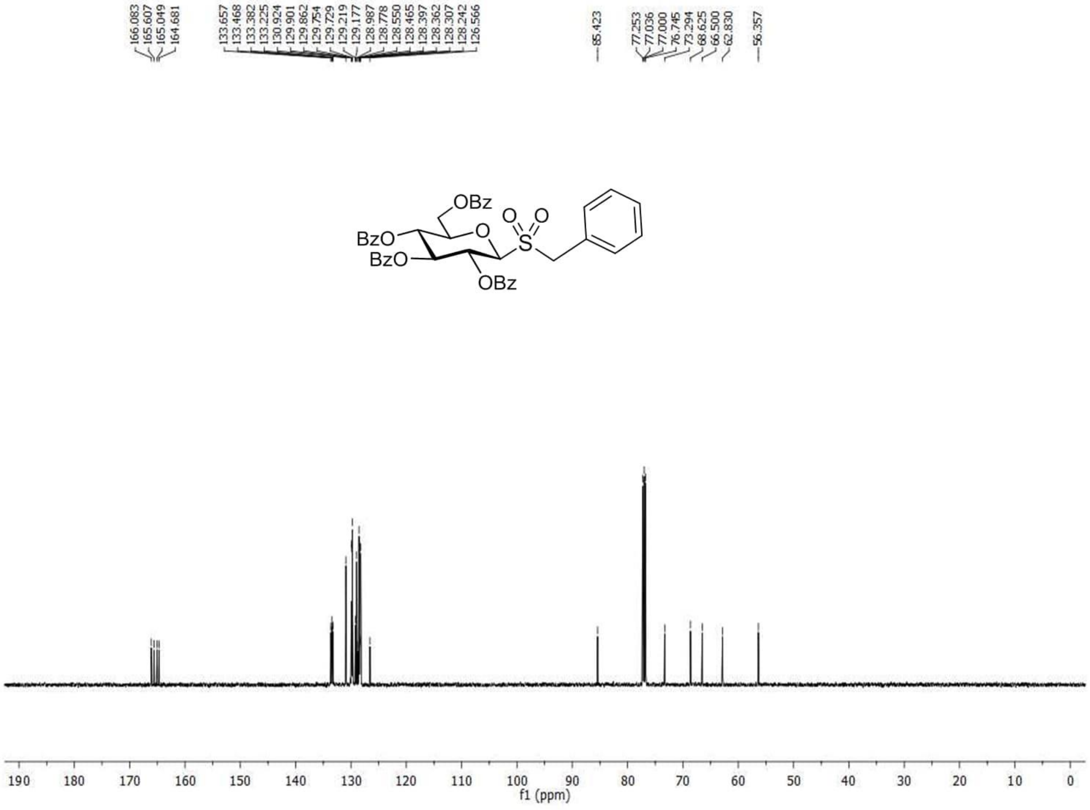

Ethylsulfonyl 2,3,4,6-tetra-*O*-acetyl-1-deoxy-β-D-glucopyranoside (7b)

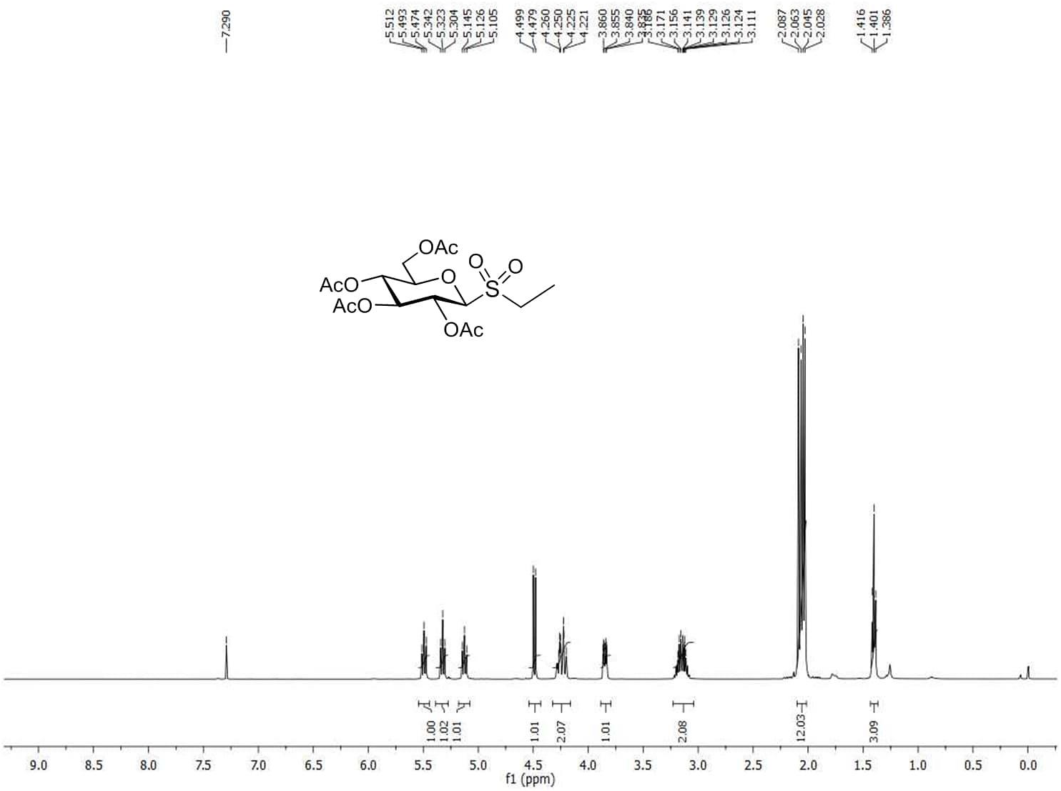

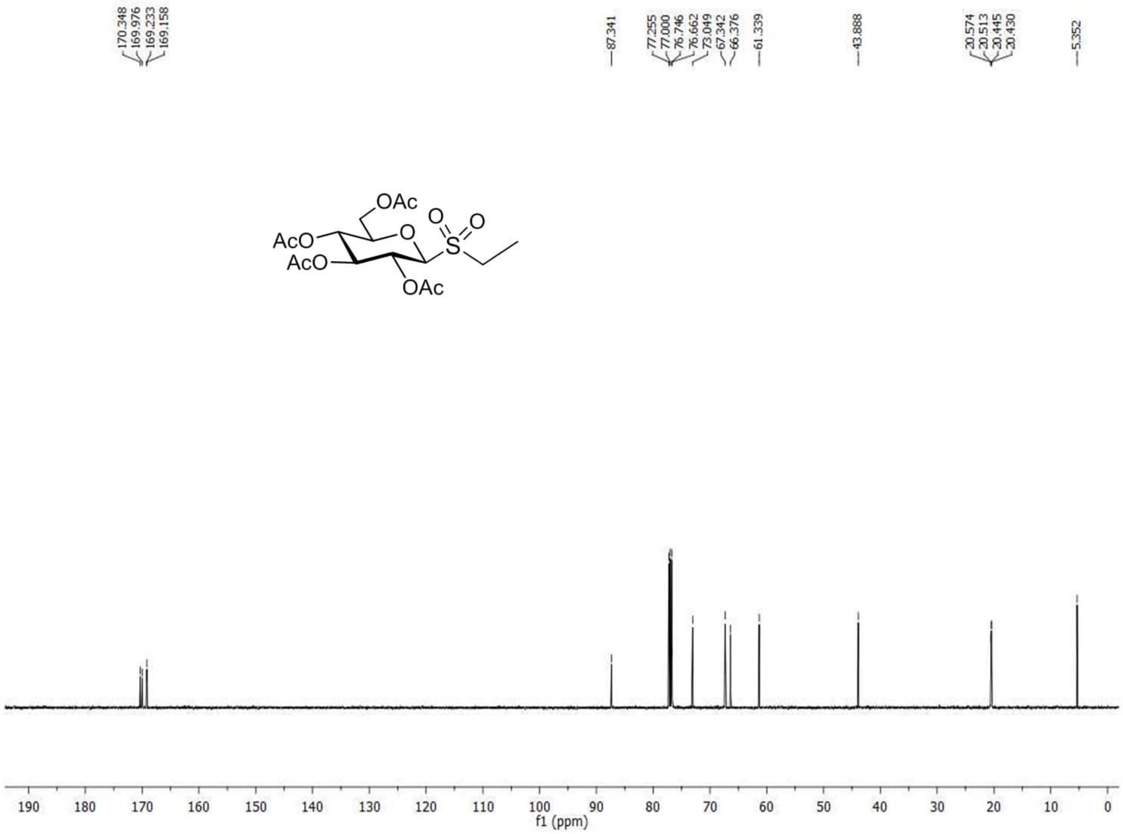

Ethylsulfonyl 2,3,4,6-tetra-*O*-benzoyl-1-deoxy-β-D-glucopyranoside (8b)

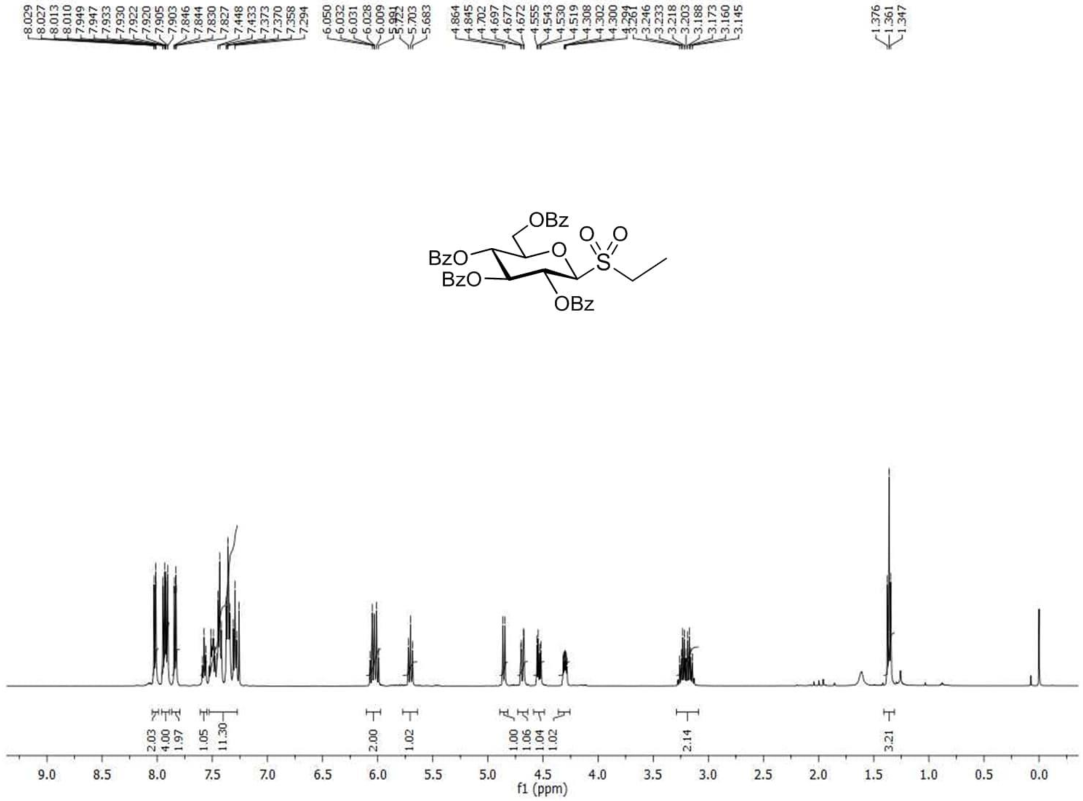

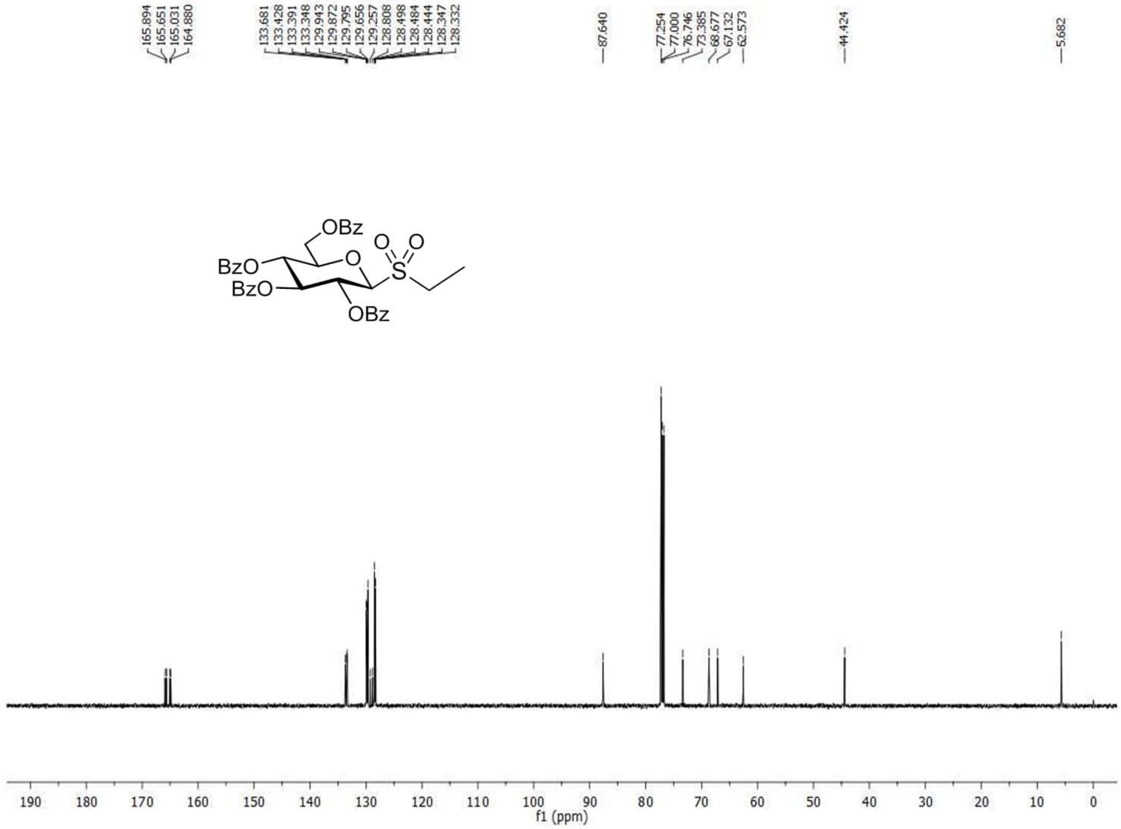

n-Hexylsulfonyl 2,3,4,6-tetra-*O*-acetyl-1-deoxy-β-D-glucopyranoside (9b)

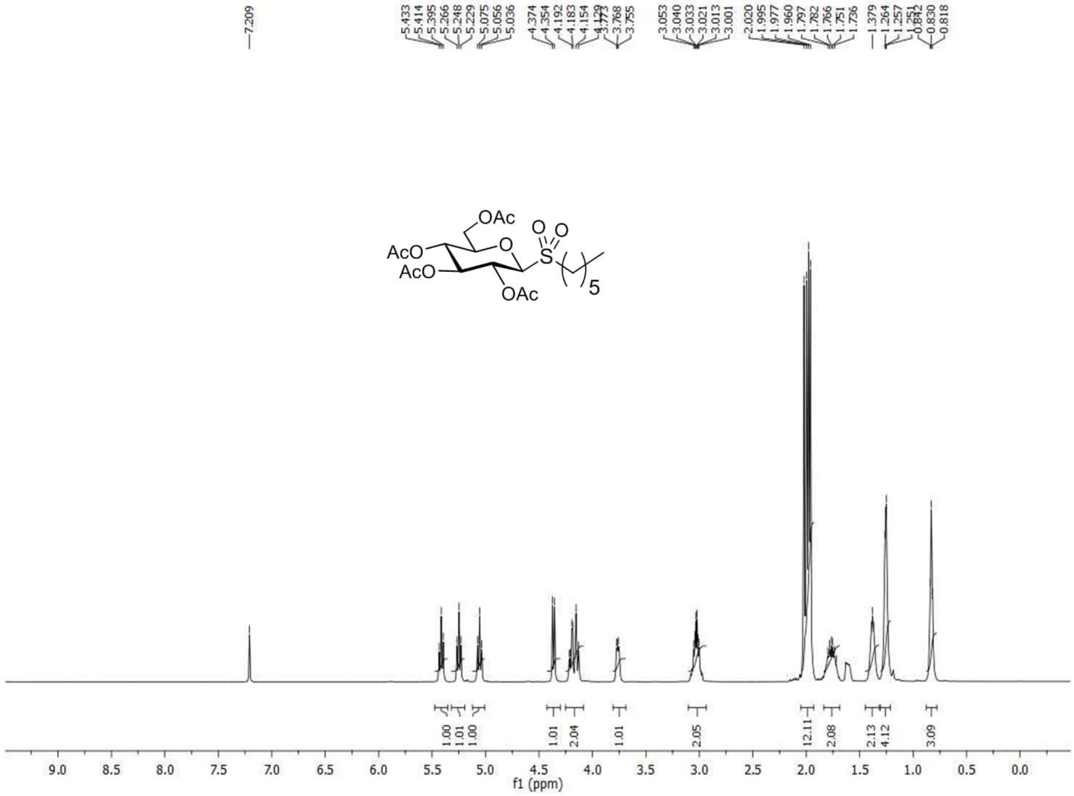

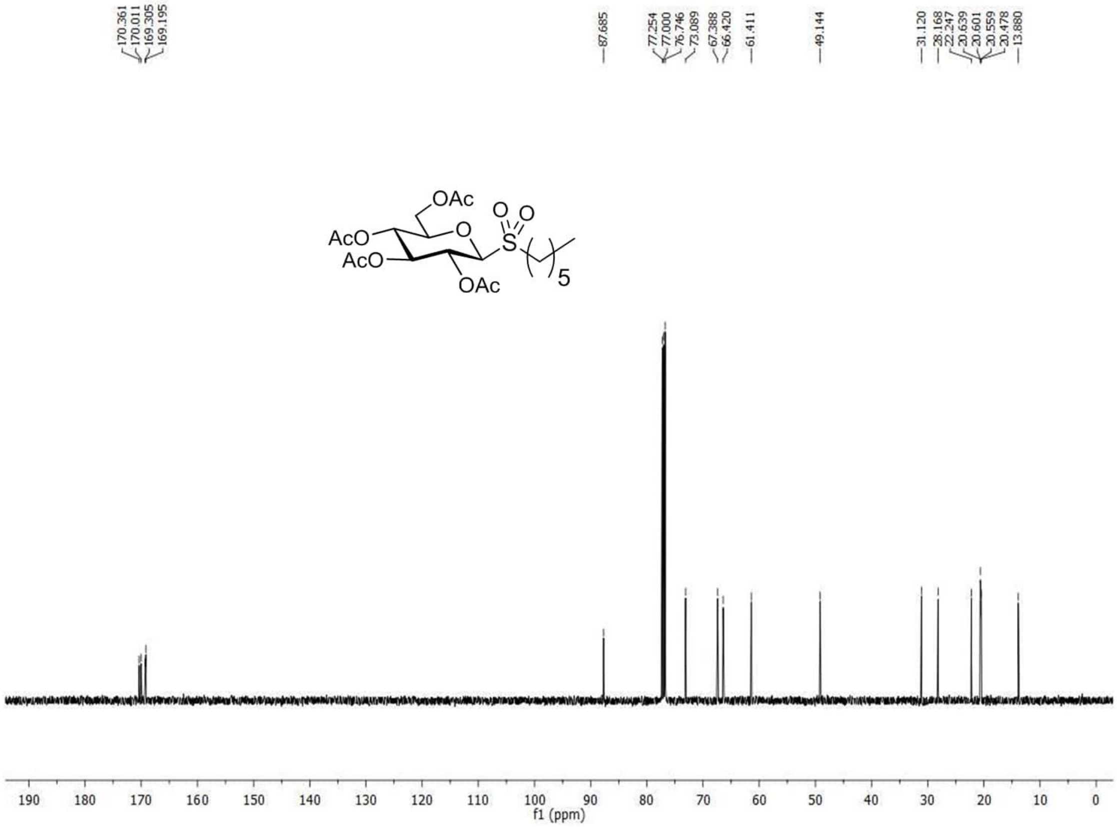

_n_-Hexylsulfonyl 2,3,4,6-tetra-_O_-benzoyl-1-deoxy-β-D-glucopyranoside (10b)

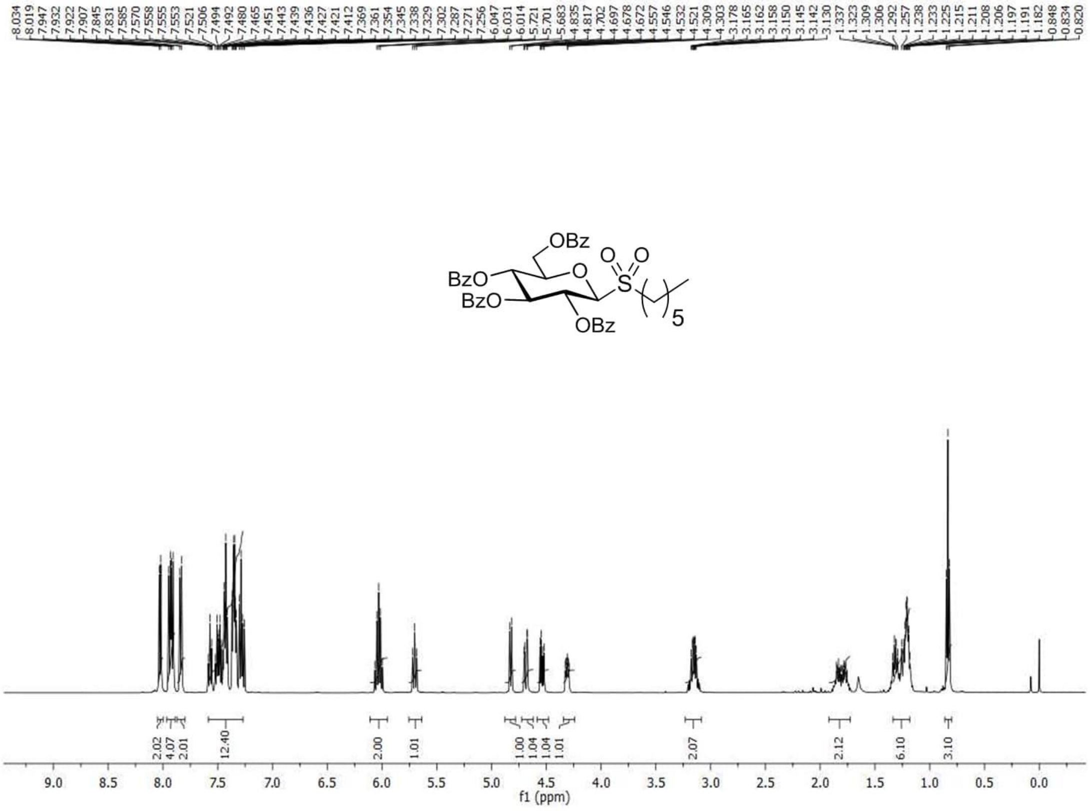

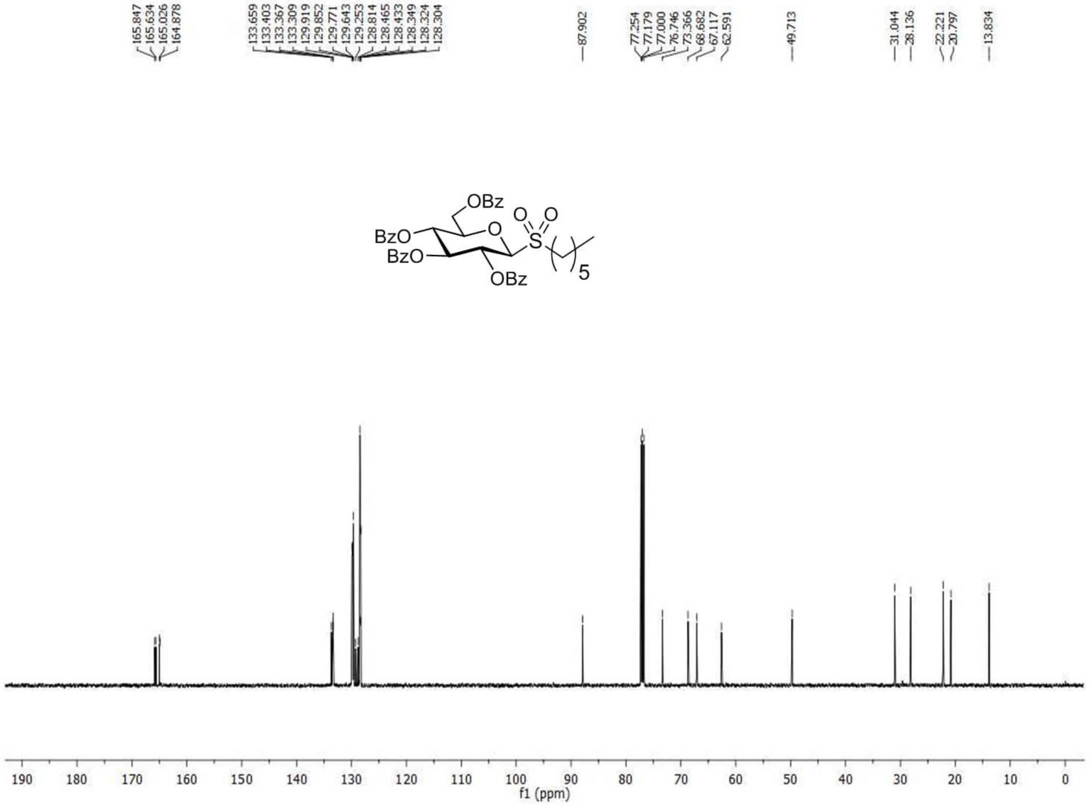

Cyclohexylsulfonyl 2,3,4,6-tetra-*O*-acetyl-1-deoxy-β-D-glucopyranoside (11b)

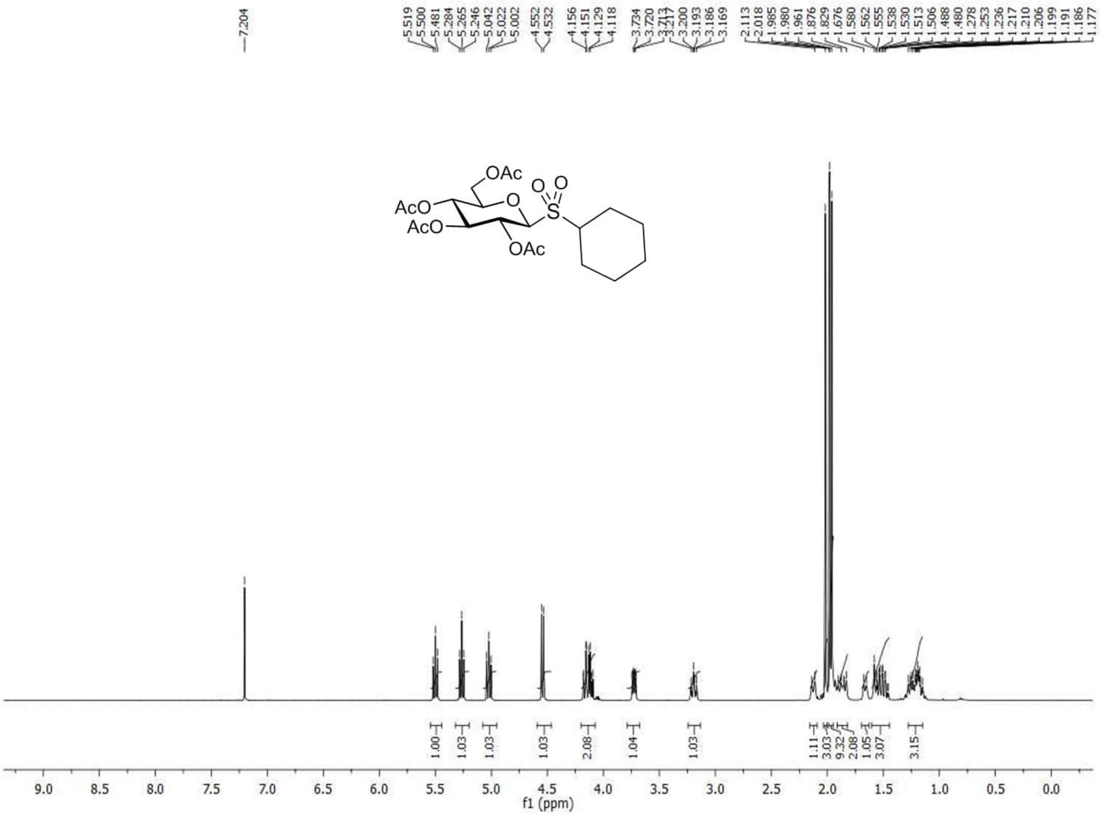

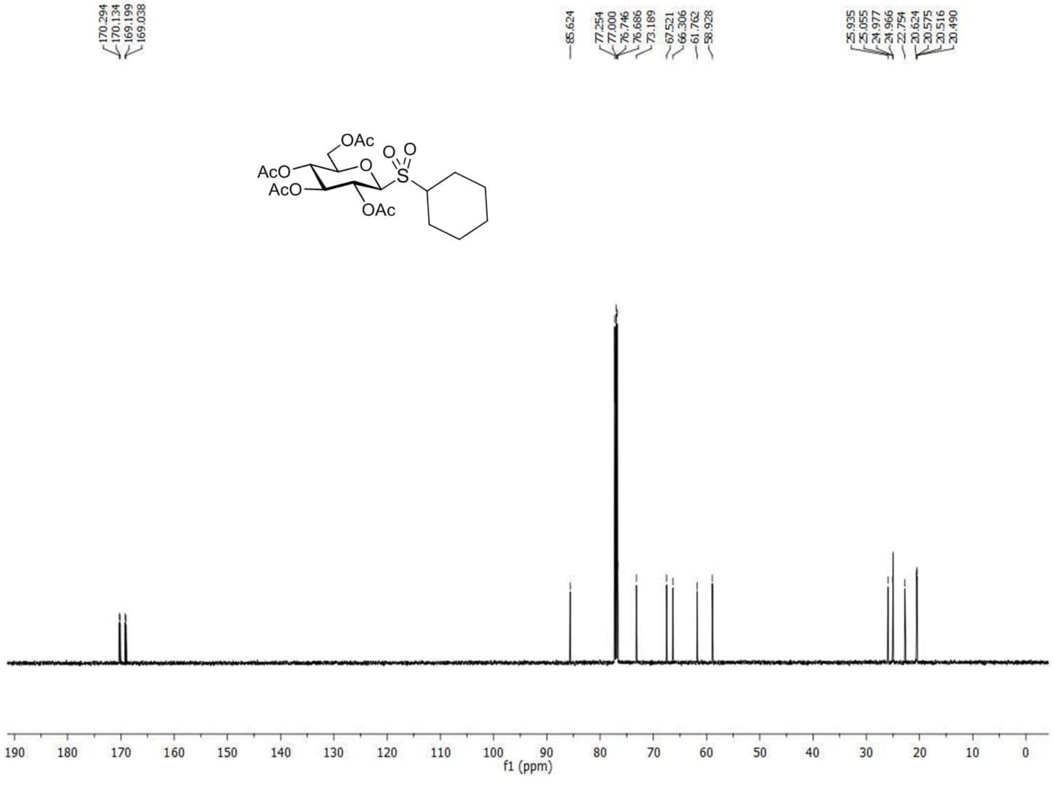

Cyclohexylsulfonyl 2,3,4,6-tetra-*O*-benzoyl-1-deoxy-β-D-glucopyranoside (12b)

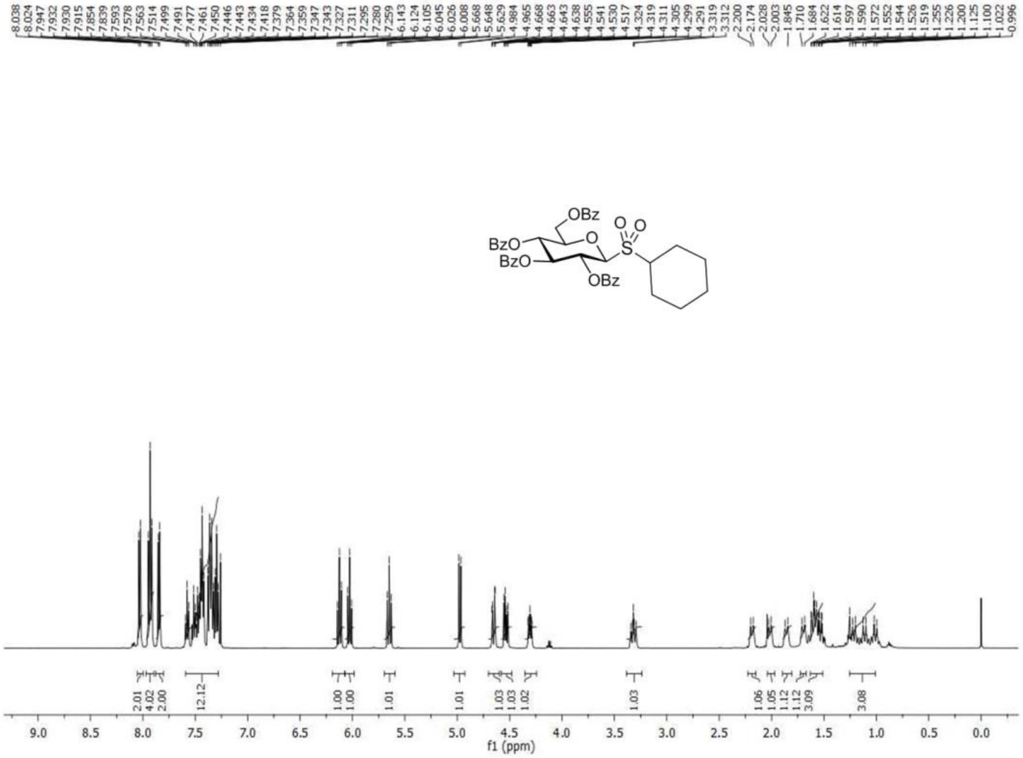

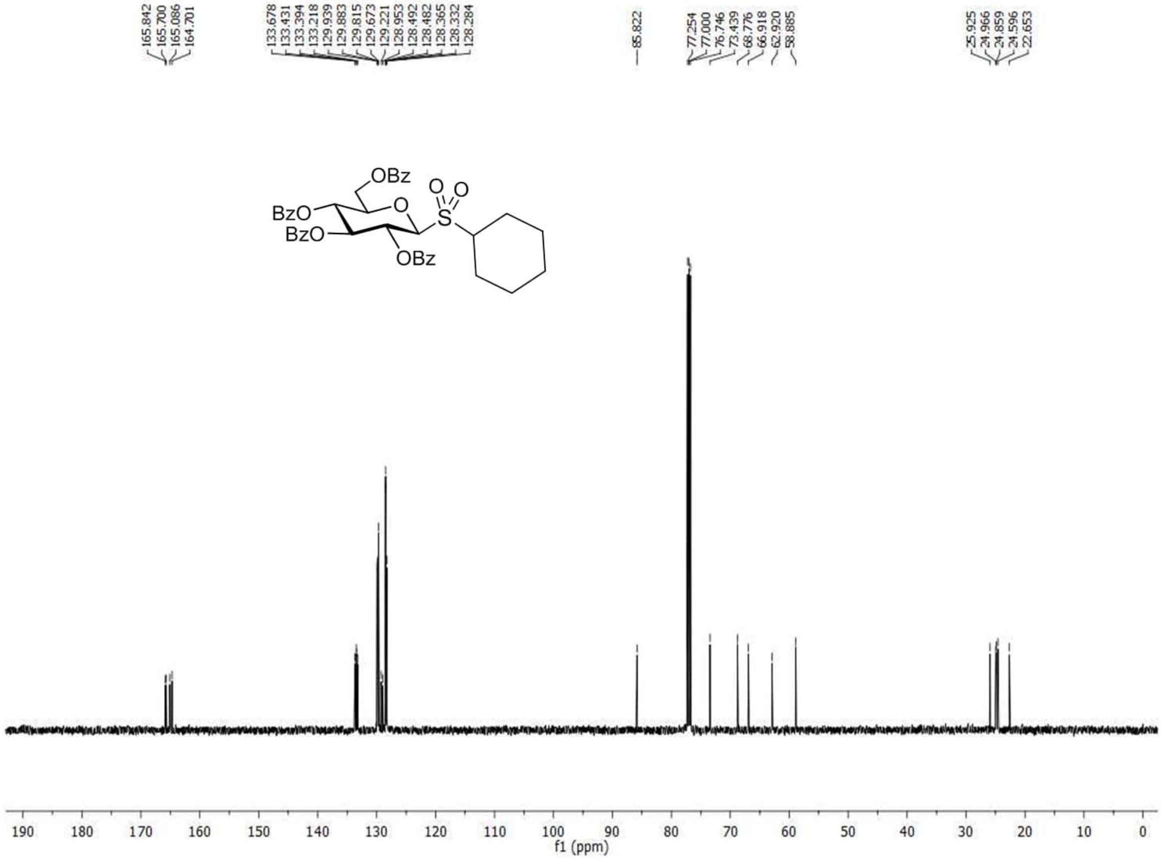

4-Methylphenylsulfonyl 2,3,4,6-tetra-*O*-benzyl-1-deoxy-β-D-glucopyranoside (13b)

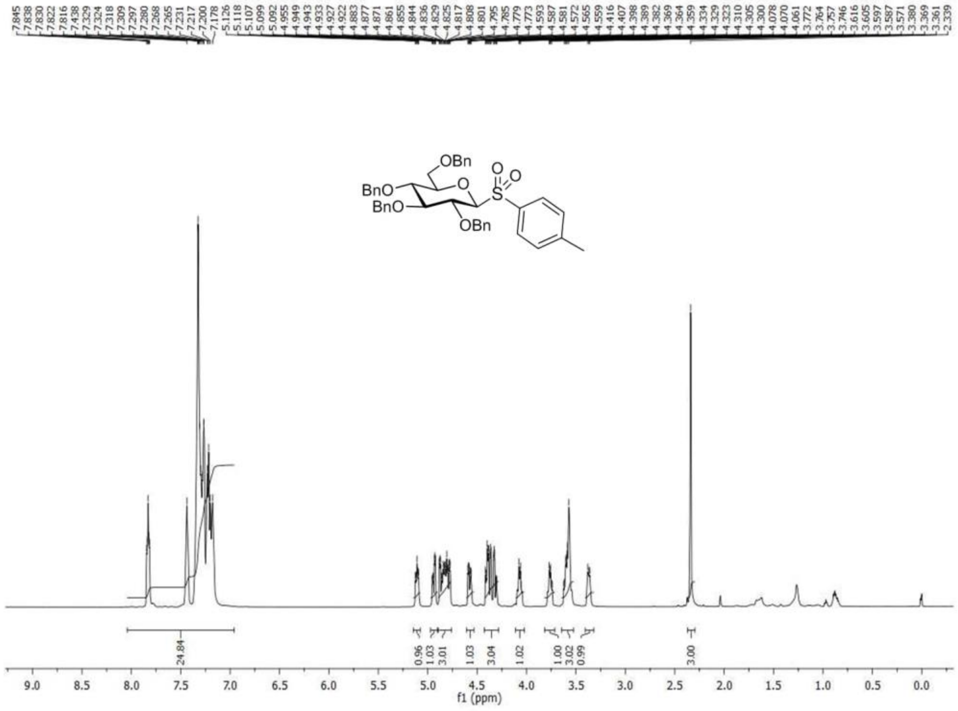

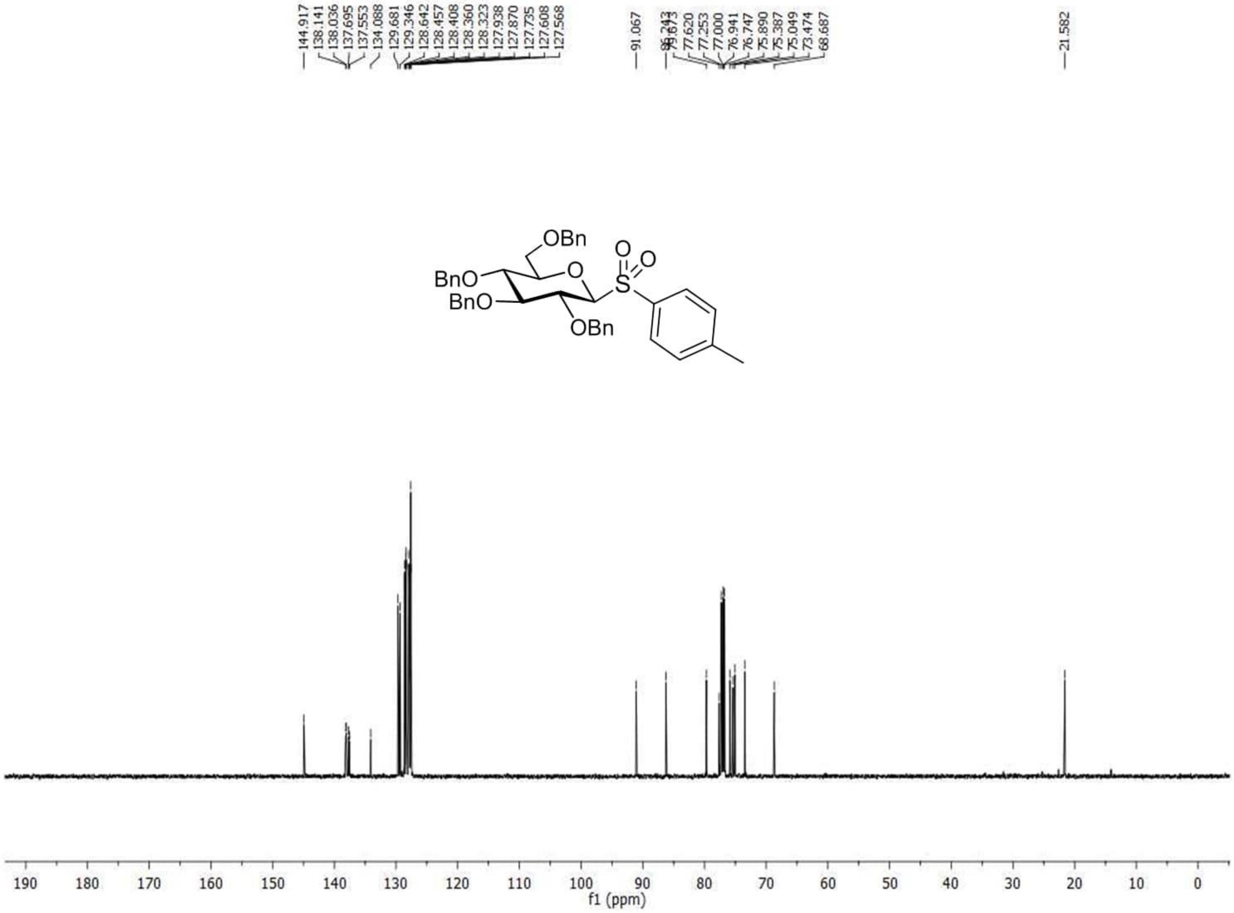

***p*-Methylphenylsulfonyl 2,3-di-*O*-allyl-4,6-di-*O*-acetyl-1-deoxy-β-D-glucopyranoside (14b)**

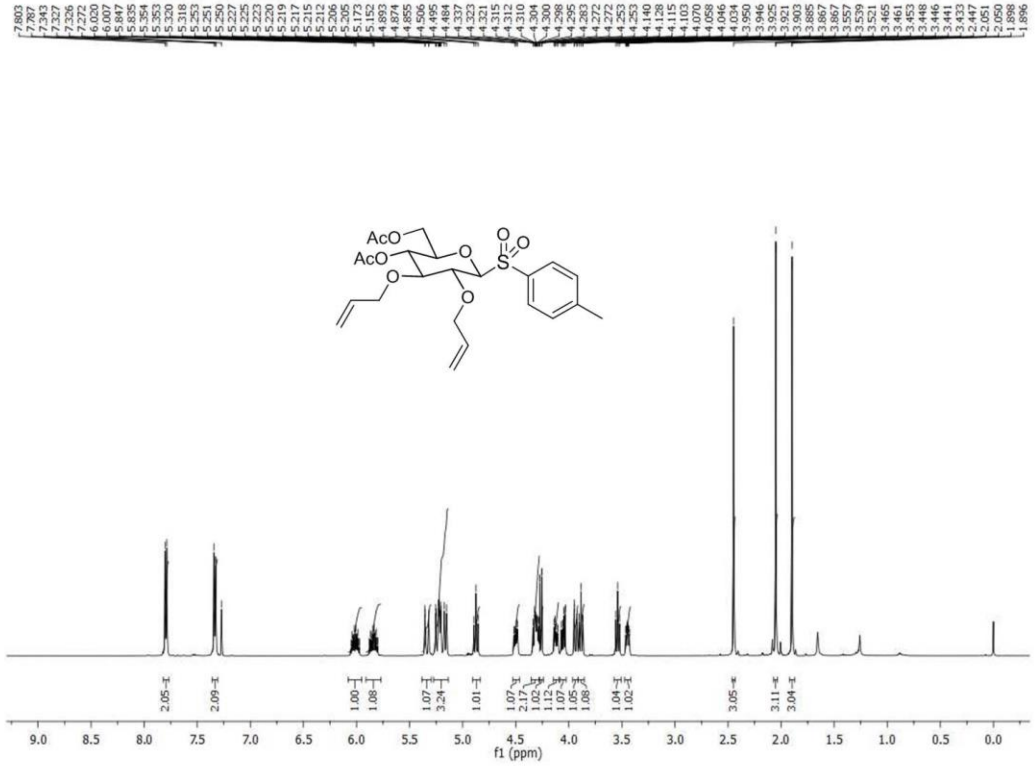

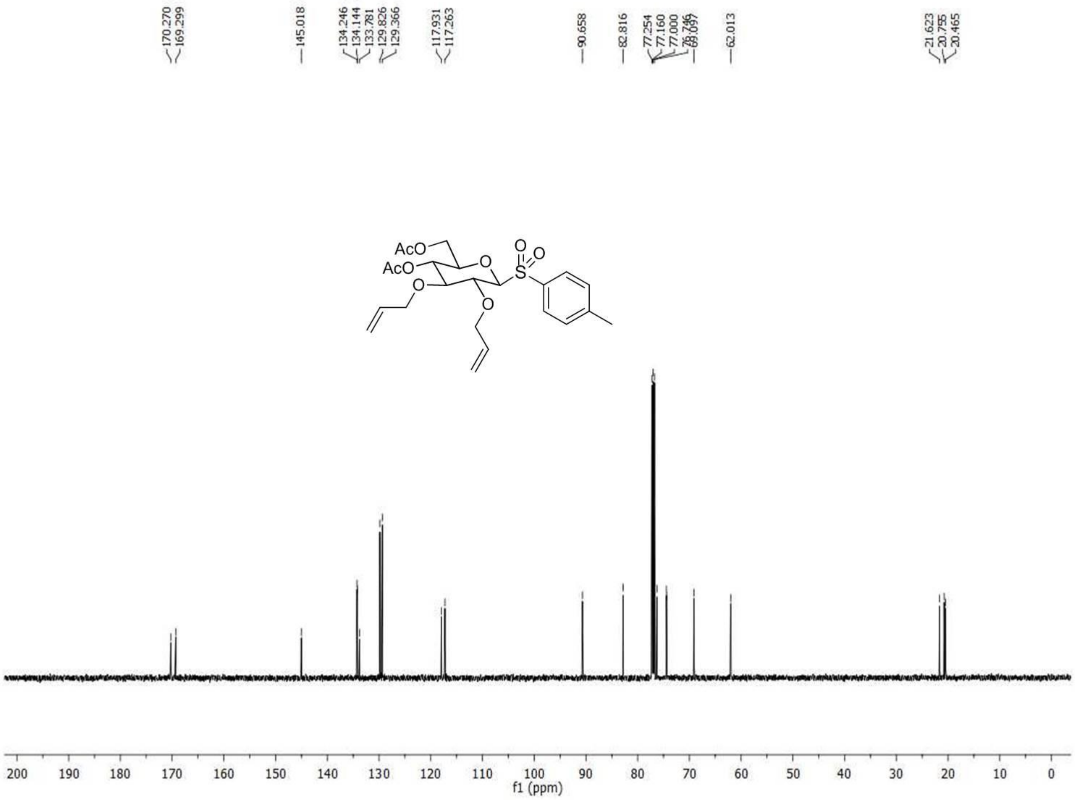

Phenylsulfonyl 3,4,6-tri-*O*-acetyl-2-*N*-phthalimido-1-deoxy-β-D-glucopyranoside(15b)

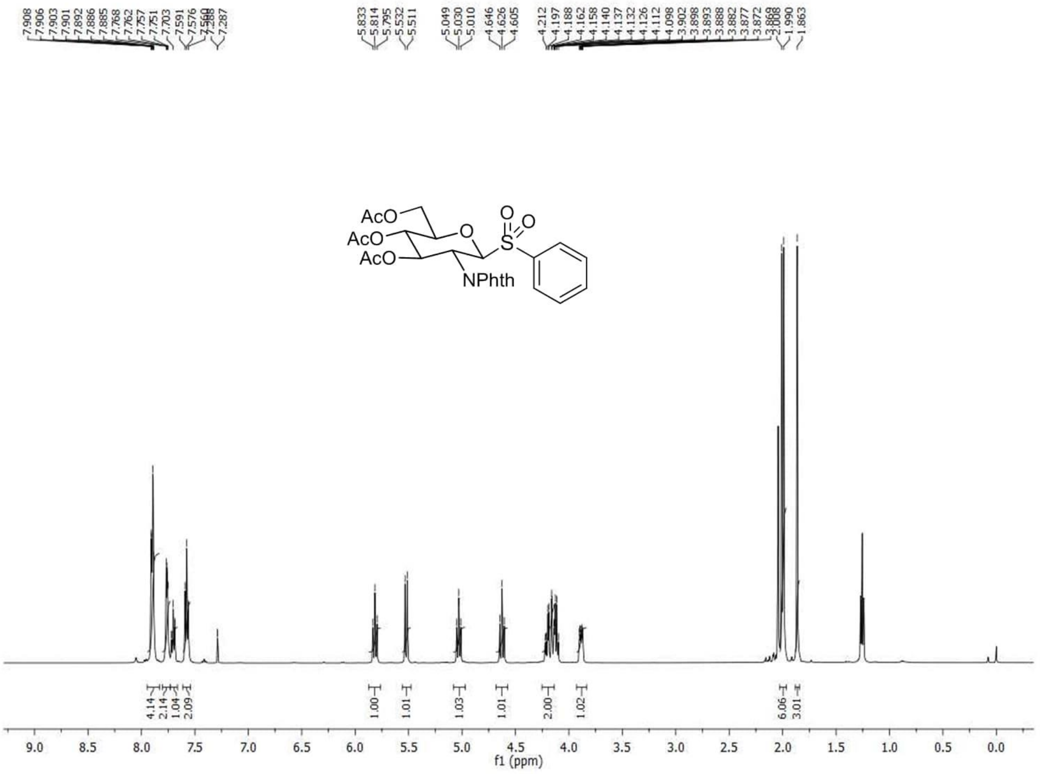

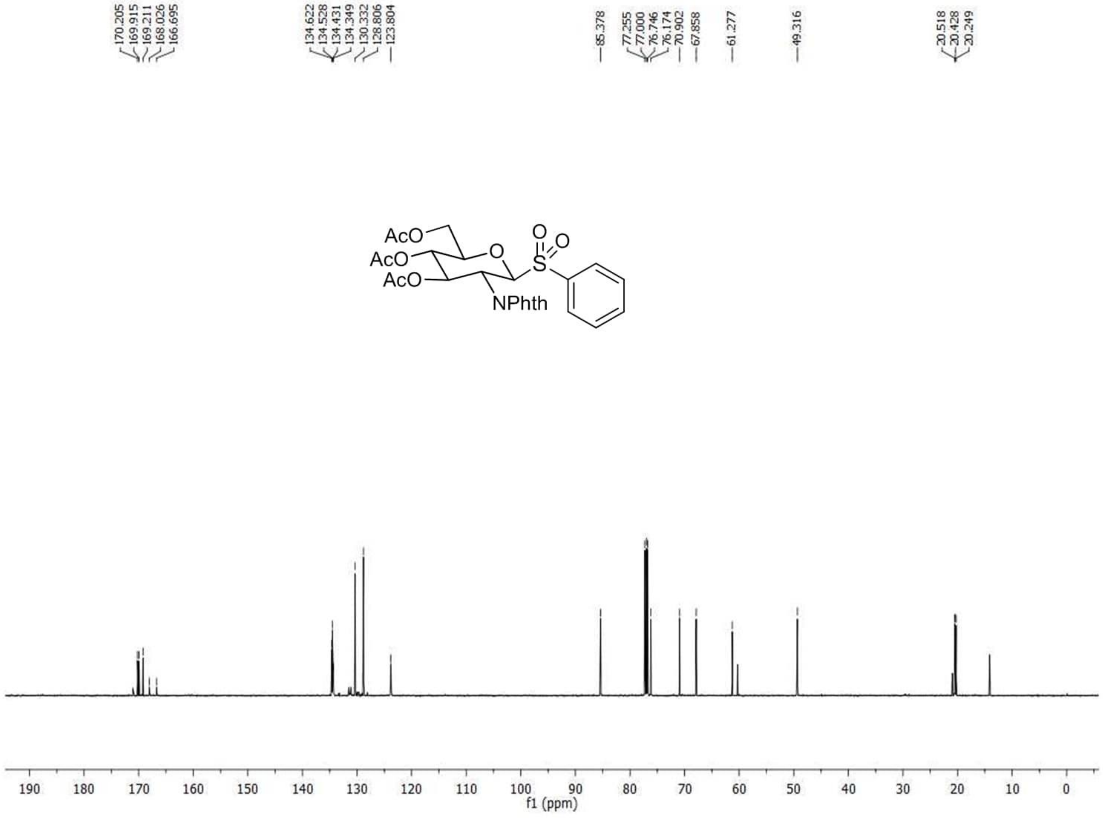

_p_-Methylphenylsulfonyl 2,3,4,6-tetra-_O_-acetyl-1-deoxy-β-D-galactopyranoside (16b)

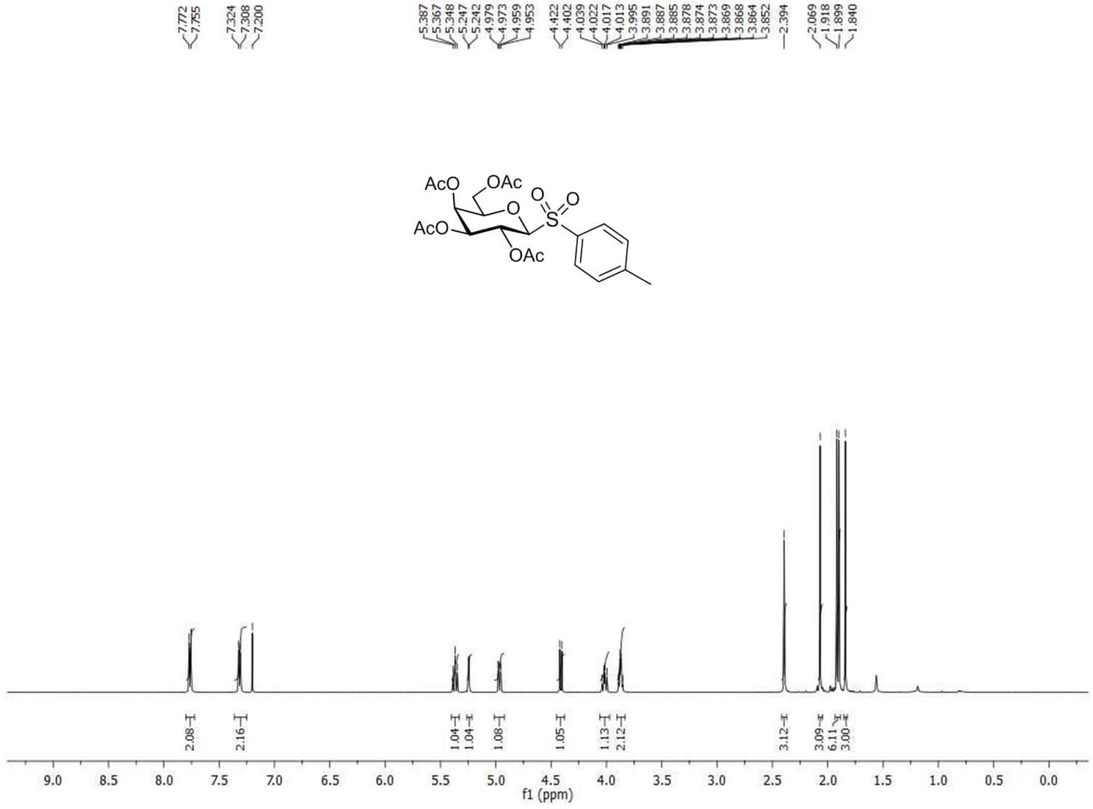

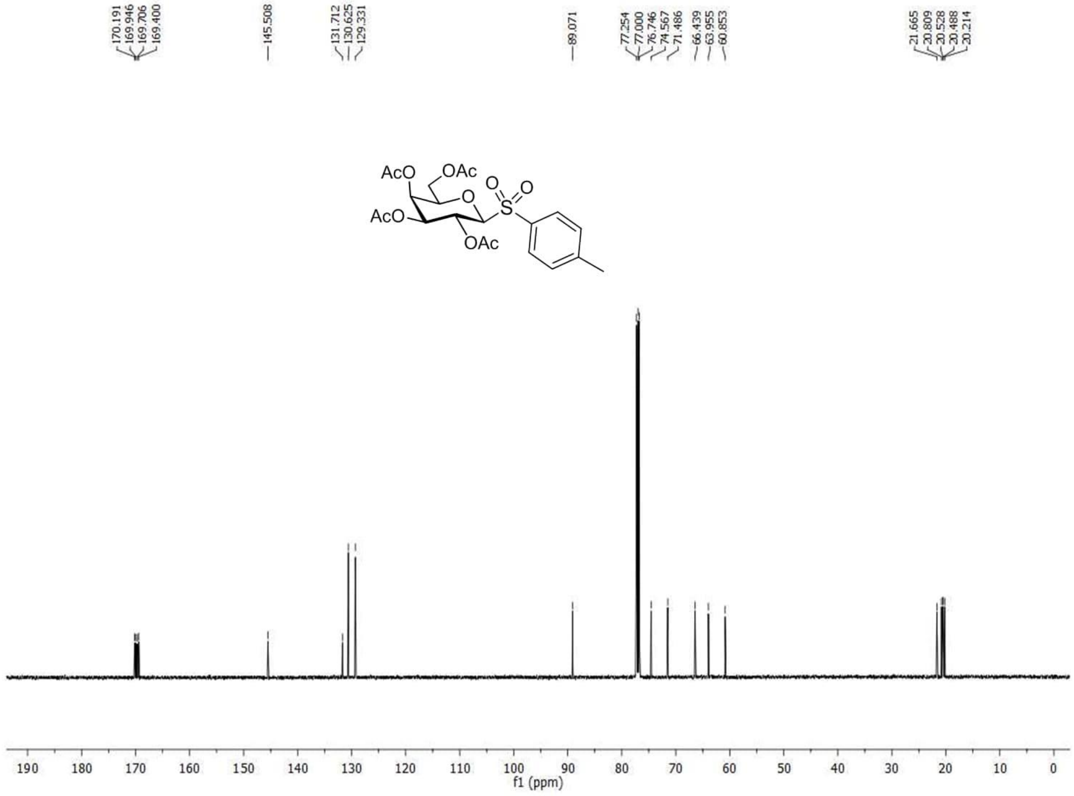

_p_-Methylphenylsulfonyl 2,3,4,6-tetra-_O_-benzoyl-1-deoxy-β-D-galactopyranoside (17b)

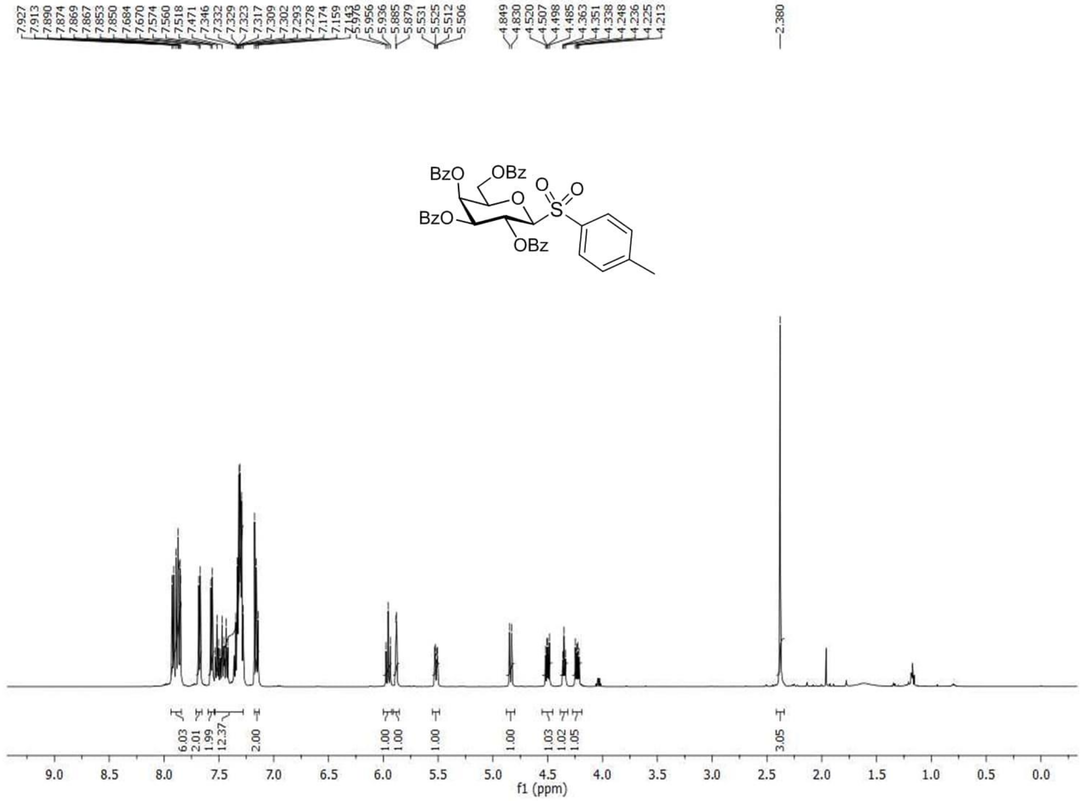

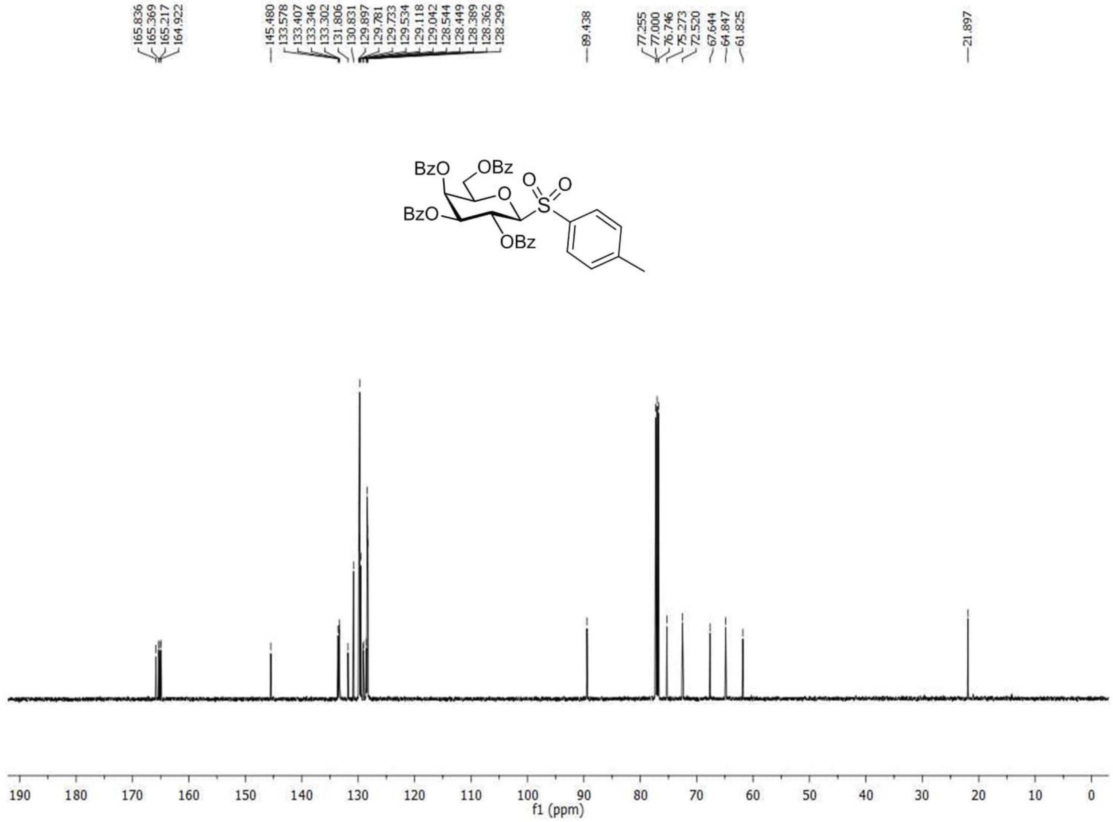

Phenylsulfonyl 2,3,4,6-tetra-*O*-acetyl-1-deoxy-α-D-mannopyranoside (18b)

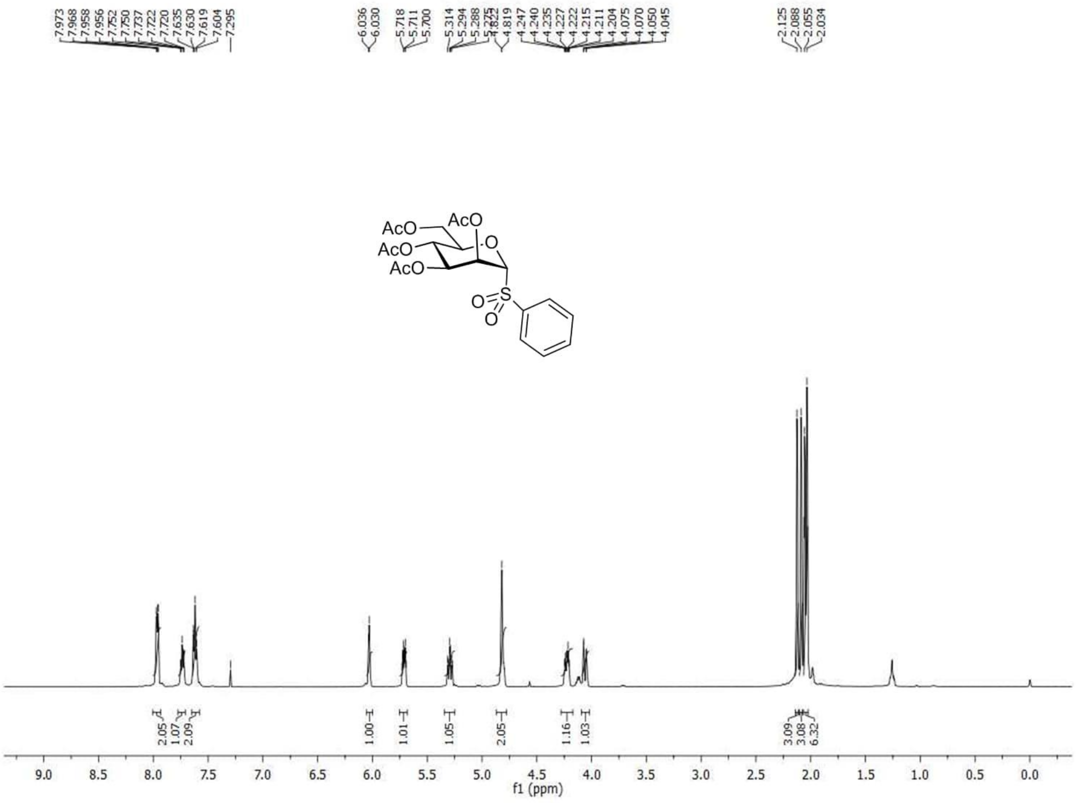

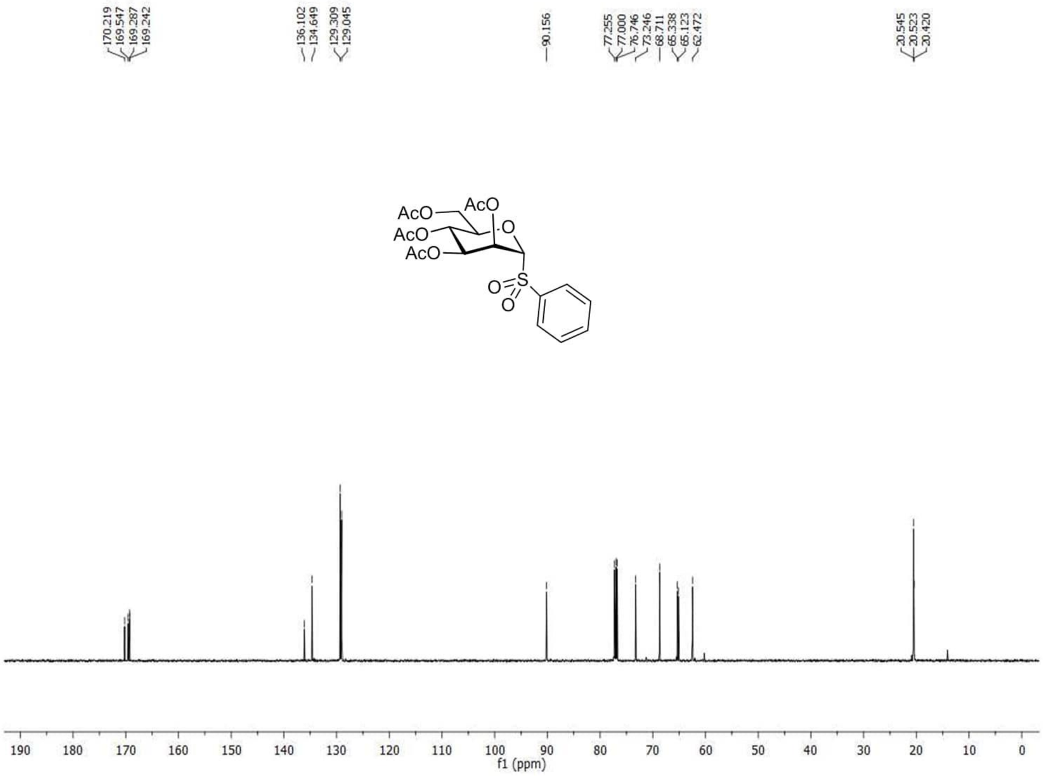

Phenylsulfonyl 2,3,4,6-tetra-*O*-benzoyl-1-deoxy-α-D-mannopyranoside (19b)

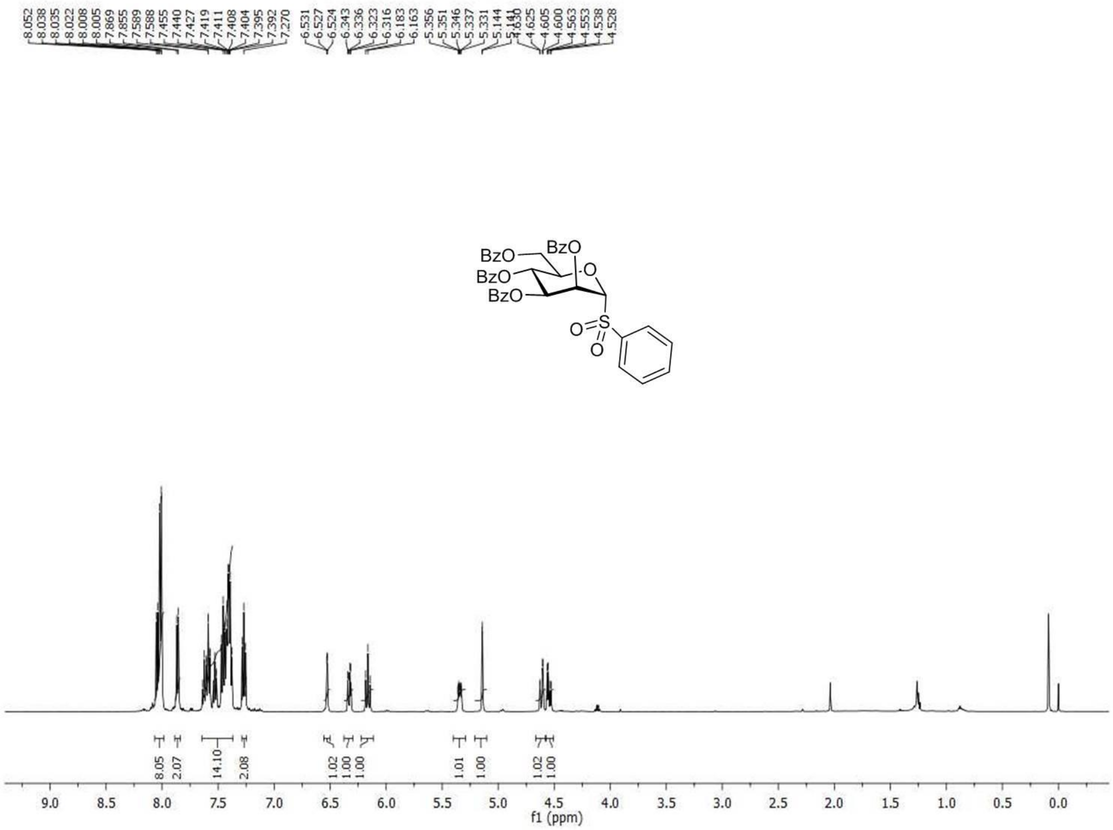

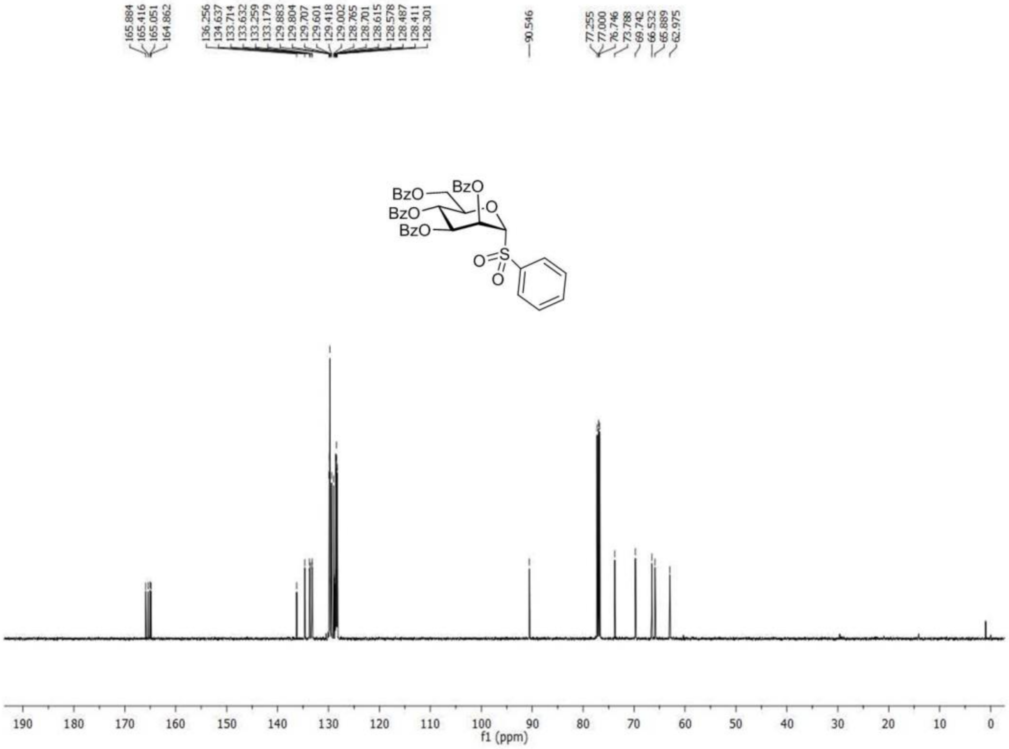

Supplement: File 1 — Experimental part and NMR spectra. [file Beilstein_J_Org_Chem-13-1139-s001.pdf]
